# Consistent and Generalizable Effective Model Hamiltonian Framework for Studying Nonadiabatic Dynamics in the Condensed Phase

**DOI:** 10.1021/acs.jctc.5c01586

**Published:** 2025-12-09

**Authors:** Zengkui Liu, Hao Zeng, Xiang Sun

**Affiliations:** † Division of Arts and Sciences, 447103 NYU Shanghai, 567 West Yangsi Road, Shanghai 200124, China; ‡ NYU-ECNU Center for Computational Chemistry at NYU Shanghai, 3663 Zhongshan Road North, Shanghai 200062, China; § Department of Chemistry, New York University, New York, New York 10003, United States; ∥ State Key Laboratory of Precision Spectroscopy, East China Normal University, Shanghai 200062, China

## Abstract

Simulating nonadiabatic dynamics in complex, condensed-phase systems presents a formidable computational challenge, demanding the development of effective model Hamiltonians that capture the essential physics of electronic and nuclear interactions. This Review charts the evolution of such models, from the foundational two-state spin-boson model to multistate Frenkel exciton models, highlighting the limitations of traditional approaches, particularly the isolated bath assumption, which neglects crucial environmental correlations. We focus on the recently developed multistate harmonic (MSH) model, a general and consistent framework for mapping information from all-atom simulations onto an effective Hamiltonian. The MSH model overcomes the shortcomings of previous models by systematically satisfying all pairwise reorganization energy constraints for a multistate system. This is achieved through a novel extension of the nuclear coordinate space, which provides a physically grounded and geometrically intuitive representation of a globally shared, correlated bath. We detail the construction of the MSH Hamiltonian and its equivalent multistate reaction coordinate (MRC) representation and discuss its applications in conjunction with various dynamical methods, including perturbative quantum master equations and semiclassical nonadiabatic dynamics approaches, as well as rate constant and time-dependent rate. The MSH/MRC models not only provide a robust platform for predictive simulations of charge and energy transfer in the condensed phase but also serve as an invaluable tool for benchmarking the accuracy of approximate quantum dynamics methods.

## Graphical abstract

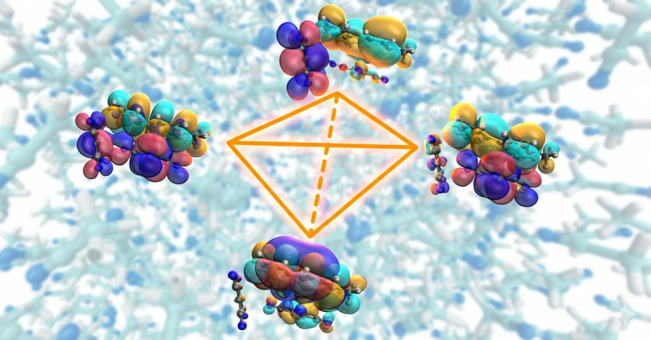

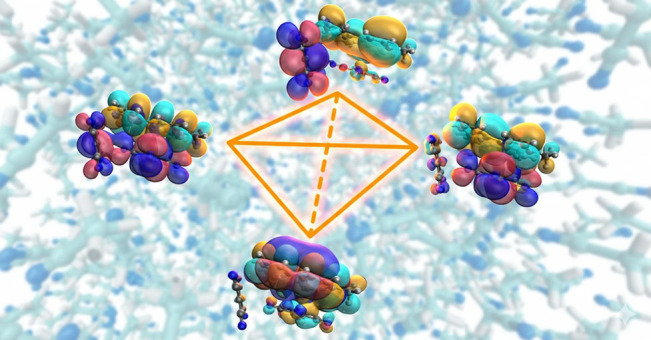

## 1 Introduction

Nonadiabatic dynamics, processes in which the Born–Oppenheimer approximation of separating electronic and nuclear motion breaks down, are fundamental to a vast variety of phenomena in chemistry,^1–9^ physics,^10–14^ and biology.^15–21^ From photoinduced charge and energy transfer in solar energy conversion materials^22–33^ to the exquisite photochemical reactions governing vision^16,34–38^ and photosynthesis,^39–44^ these processes are characterized by the interplay between multiple coupled electronic states and the motion of atomic nuclei. Simulating these quantum mechanical dynamics in complex, condensed-phase systems presents a formidable theoretical and computational challenge.^45–53^ The sheer number of coupled electronic and nuclear degrees of freedom (DOF) in a realistic system, such as a chromophore dissolved in a solvent or embedded in a protein matrix,^39,54–58^ leads to a Hilbert space whose dimensionality scales exponentially with system size. This “curse of dimensionality” renders numerically exact quantum simulations intractable for all but the simplest of systems.^59–68^ This computational barrier calls for the development of effective, yet physically grounded, model Hamiltonians that can capture the essential physics of the nonadiabatic process without the prohibitive cost of an all-atom quantum treatment.

The development of such effective Hamiltonians represents more than a mere computational convenience: it is a theoretical imperative that forces a conceptual distillation of which environmental features are critical for a given quantum process.^69–76^ The conventional and most powerful approach to constructing an effective model is to partition the entire complex system into a “system” of primary interest and a “bath” representing its environment.^77,78^ In the context of photochemical processes, the electronic DOF of the chromophore(s) are typically designated as the system, while the vast number of nuclear DOF, comprising both intramolecular vibrations and the intermolecular vibrations involving the surrounding solvent or protein scaffold, constitutes the bath. The system-bath coupling is an essential component of the model construction, as it dictates how population and coherence are exchanged between the electronic states and the system’s energy dissipation to the bath.

To make the description of the high-dimensional bath tractable, the harmonic approximation is commonly employed, where the potential energy surfaces (PESs) associated with the bath are represented by a collection of harmonic or normal modes. The underlying principle that validates this approach, particularly for condensed-phase systems, is the central limit theorem.^79–84^ In a condensed-phase environment like a liquid or a protein, a large number of weak, quasi-random interactions from the surrounding molecules collectively influence the electronic system. The cumulative effect of these many small perturbations can be well-approximated by a Gaussian statistical process, which is the defining characteristic of a harmonic bath.^85–88^ This approximation is particularly more robust in the condensed phase than in isolated gas-phase molecules, where a few specific, large-amplitude anharmonic motions, such as torsional motion, might play a dominant role in the dynamics.^89–92^ Representing the atomistic anharmonic nuclear PES in terms of a collection of harmonic modes is a profound theoretical step, enabling analytical integration of the nuclear dynamics^93,94^ and providing a platform to benchmark with numerically exact nonadiabatic quantum dynamics.^65,95,96^

The strategy of mapping a complex, all-atom anharmonic system onto a simplified model with harmonic bath modes has been exceptionally successful, most remarkably for the widely used spin-boson model,^97–100^ which describes a two-state quantum system coupled to a dissipative environment. This model, arguably the most prevalent for describing electronic transitions in the condensed phase, maps the all-atom Hamiltonian onto a two-level system bilinearly coupled to a harmonic bath. The parameters of this bath, encapsulated in a spectral density function, are not arbitrary but are systematically derived from the energy gap time correlation function (TCF) of the whole all-atom system.^100–103^ The bilinear coupling form, which is a direct product of an electronic operator and a linear nuclear operator, is a central feature of this model, arising from the assumption of linear response of the bath to the change in electronic state.^77,81,104^ This coupling is quantified by the reorganization energy, which is directly proportional to the variance of the energy gap fluctuations, and thus provides a direct link between the model parameters and the all-atom simulation data. A pictorial representation of the spin-boson model describes the system in terms of two harmonic PESs whose minima are displaced relative to each other. The magnitude of this displacement along each harmonic mode is determined by the system-bath coupling coefficients,^105,106^ providing a clear geometric picture of how the global nuclear environment reorganizes in response to an electronic transition.

Extending the successful two-state paradigm to systems involving multiple electronic states, however, presents significant conceptual and practical challenges. The primary issue lies in unambiguously determining the system-bath, or the electronic-nuclear, coupling. In most realistic multistate systems, the bath Hamiltonian cannot be uniquely defined conventionally. For instance, one could choose the ground state PES as a straightforward reference for the bath Hamiltonian, but this choice may not be appropriate for certain photoinduced processes where the ground state is not directly involved in the subsequent dynamics.^106^ This choice can also be problematic for certain quantum dynamical methods whose results depend on how the state-independent part of the nuclear DOF is defined.^107–109^ While the bilinear coupling approach is well-defined for the two-state spin-boson model, for multistate systems, the partition of the bath Hamiltonian and the system-bath coupling Hamiltonian is unclear. For example, a standard approach, as in the Frenkel exciton model for molecular aggregates, is to make a significant assumption, which is known as the isolated bath harmonic (IBH) model defined within the local bath bases.^110^ The Frenkel exciton model is foundational to the study of excitons in multichromophoric systems like photosynthetic complexes, which treats each chromophore as being embedded in its own local, independent bath.^111,112^ In the IBH framework, different chromophores do not share their bath modes, and the baths belonging to different electronic sites are treated as completely independent and noninteracting.

The IBH approach, while greatly simplifying the theoretical description, fails to describe more general and realistic situations where the environments of different electronic states are correlated. For example, if chromophores are in proximity, as they are in a structured environment, the motion of a local group of protein side chains or solvent molecules can simultaneously modulate the site energies of multiple nearby chromophores, inducing a correlated bath effect that the IBH model neglects by design.^113–117^ Furthermore, the IBH model is inadequate for describing a single chromophore that has multiple electronically excited states that are important for the dynamics, such as a bright excitonic state and multiple dark charge-transfer (CT) states, as these states must necessarily be coupled to the same, shared molecular environment. These limitations also apply to systems of multiple interacting chromophores, each with several relevant excited states. In these cases, where correlated bath effects are physically significant,^113–115,118,119^ the IBH strategy is insufficient, in part because its perspective is based on the local (excited) state of an individual chromophore site rather than the global electronic states of the entire coupled system.

A systematic and physically rigorous way of describing these heterogeneous, correlated bath effects is provided by the recently developed multistate harmonic (MSH) model.^119,120^ The MSH construction is based on a fundamentally different perspective: it treats electronic transitions between pairs of global electronic states. In this framework, the electronic-nuclear coupling is understood to be intrinsically associated with a specific electronic transition between two states, and the nuclear modes are not localized to one moiety or molecular site but rather of the global environment. Therefore, for a general *F*-state problem, the crucial all-atom information consists of the properties of the *F*(*F* – 1)/2 possible pairs of states. A physically consistent model must incorporate all of this information, such as the corresponding reorganization energy for each pair, which quantifies the energy cost for the nuclear environment to relax from the equilibrium configuration of one state to that of the other. The MSH Hamiltonian is constructed by simultaneously satisfying all *F*(*F* – 1)/2 of these all-atom reorganization energy constraints, a feature that is impossible with traditional models. This is achieved through the central innovation of the MSH model: the extension of the nuclear dimensionality to (*F* – 1) dimensions for each physical normal mode in an *F*-state problem. This dimensional extension is a physical necessity, as a standard one-dimensional bath mode lacks the degrees of freedom to encode the complex network of *F*(*F* – 1)/2 correlations. The result is a geometrically intuitive picture where the PES minima of the *F* electronic states form a polyhedron in an extended nuclear space, with the geometry of the polyhedron directly encoding the heterogeneous, correlated nature of the bath. The two key points are the globally shared bath basis and the correctly reproduced reorganization energy relationship.

More importantly, the effectiveness of any model Hamiltonian should be judged by comparing the nonadiabatic dynamics it produces against the dynamics obtained with the full, all-atom multistate Hamiltonian, which is a validation step that was often overlooked.^121–123^ An effective model must not only be computationally tractable but also physically predictive. We will show that the nonadiabatic dynamics simulated using the MSH model Hamiltonian can faithfully reproduce the result from an all-atom Hamiltonian for photoinduced charge transfer (PICT) reactions in a liquid solution, for example, the carotenoid-porphyrin-fullerene (CPC_60_) triad^119,124–133^ dissolved in explicit solvent. In stark contrast, the conventional IBH model, when applied to the same system, fails to capture the correct dynamics, demonstrating the physical importance of the heterogeneous bath effects that are rigorously included in the MSH framework.^134^

In the remainder of this Review, we will first review the fundamental models of the spin-boson model and Frenkel exciton model in Section 2, and then introduce the construction of the MSH model from all-atom input in Section 3. Furthermore, in Section 4, we will describe the multistate reaction coordinate (MRC) model,^135^ which is an exactly equivalent representation of the MSH model that offers important physical insights through the concept of a multidimensional reaction coordinate for general nonadiabatic dynamics in the condensed phase. In Section 5, we will describe recent applications of the MSH and MRC model Hamiltonians in simulating quantum dynamics and benchmarking approximate methods. In Section 6, we offer a broader discussion, including the distinction of the MSH/MRC framework from other advanced correlated bath models, and an overview of the current limitations and future extensions of the MSH/MRC framework, before providing concluding remarks in Section 7. In Appendix A, we detail the PES minima of F-state MSH model.

## 2 A Genealogy of Foundational Model Hamiltonians

### 2.1 Spin-Boson Model

We begin with the spin-boson model, a widely used framework for describing electronic transitions in a two-level system embedded in a condensed-phase environment.^97–99^ The spin-boson Hamiltonian in symmetric form is given by

1 $$Ĥ=\Gamma {\mathrm{\sigma ̂}}_{x}+\varepsilon {\mathrm{\sigma ̂}}_{z}+\overset{N}{\underset{j=1}{\sum }}\left(\frac{{\mathrm{P̂}}_{j}^{2}}{2}+\frac{1}{2}\omega_{j}^{2}{\mathrm{R̂}}_{j}^{2}-c_{j}{\mathrm{R̂}}_{j}{\mathrm{\sigma ̂}}_{z}\right)$$

where σ̂_*x*_ = |1⟩⟨2| + |2⟩⟨1| and σ̂_*z*_ = |1⟩⟨1| – |2⟩⟨2| are Pauli operators defined in the basis of the two diabatic electronic states |1⟩ and |2⟩; Γ denotes the electronic coupling coefficient; Δ*E* = −2*ε* is the reaction free energy; {*R̂*_*j*_, *P̂*_*j*_, ω_*j*_} = {*R̂*_*j*_, *P̂*_*j*_, ω_*j*_ | *j* = 1,···,*N*} are the mass-weighted coordinates, momenta, and frequencies of the *N* nuclear normal modes; and {*c*_*j*_} are the electronic–nuclear coupling coefficients.

Eq 1 can be decomposed into system, bath, and system–bath coupling Hamiltonians, *Ĥ* = *Ĥ*_sys_ + *Ĥ*_bath_ + *Ĥ*_bs_, where

2 Ĥsys=(εΓΓ−ε),Ĥbath=∑j=1N(P̂j22+12ωj2R̂j2)Ĥbs=(−∑j=1NcjR̂j00∑j=1NcjR̂j)

The bath vibrational frequencies {ω_*j*_} and electronic–nuclear coupling coefficients {*c*_*j*_} are characterized by the spectral density,^97,136^

3 $$J\left(\omega \right)=\frac{\pi }{2}\overset{N}{\underset{j=1}{\sum }}\frac{c_{j}^{2}}{\omega_{j}}\delta \left(\omega -\omega_{j}\right)$$

which can be specified either through closed-form expressions with empirical parameters,^98,111,112,137,138^ e.g., Ohmic spectral density $J\left(\omega \right)=\frac{\pi }{2}\hbar \xi \omega e^{-\omega /\omega_{c}}$ , where ξ is the Kondo parameter and ω_*c*_ is the cutoff frequency, or more realistically obtained directly from all-atom molecular dynamics (MD) simulations by Fourier transforming the TCF of energy-gap fluctuations:^100,105^

4 $$J\left(\omega \right)=\frac{\beta \omega }{4}\int_{0}^{\infty }dtC_{UU}\left(t\right)\cos\left(\omega t\right)$$

where the inverse temperature is β = 1/*k*_B_ *T* (*k*_B_ is the Boltzmann constant and *T* the temperature), and the TCF of the energy gap *U*(**R**) = *V*_1_(**R**) – *V*_2_(**R**) is

5 $$C_{UU}\left(t\right)=\left\langle U\left(t\right)U\left(0\right)\right\rangle -\langle U\rangle^{2}$$

When the spectral density is obtained from MD simulations, its discretization into *N* (*N* ≫ 1) nuclear normal modes involves solving, for each *j*-th mode (*j* = 1, 2,···,*N*), the equation^100,105^

6 $$\frac{2N\omega_{j}}{\pi C_{UU}\left(0\right)}\int_{0}^{\infty }dt\frac{C_{UU}\left(t\right)}{\omega_{j}t}\sin\left(\omega_{j}t\right)=j-\frac{1}{2}$$

Once ω_*j*_ is determined, the corresponding coupling coefficient *c*_*j*_ follows from

7 $$c_{j}=\sqrt{\frac{E_{r}}{2N}}\omega_{j},\left(j=1,2,...,N\right)$$

yielding a discrete frequency set of the entire bath corresponding to an equal partitioning of the reorganization energy.

The reorganization energy between the two electronic states is proportional to the variance of the energy gap, *C*_*UU*_(0) = ⟨*U* ^2^⟩ – ⟨*U*⟩^2^, and is equivalently expressed as

8 $$E_{r}=\frac{C_{UU}\left(0\right)}{2k_{B}T}=\frac{4}{\pi }\int_{0}^{\infty }d\omega \frac{J\left(\omega \right)}{\omega }$$

Physically, *E*_*r*_ originates from the displacement of the minima of the PESs of the two electronic states, and can be obtained by evaluating the energy difference on the same PES (either *V*_1_ or *V*_2_) at the two geometries corresponding to their respective minima.

An alternative, more intuitive, representation of the spin-boson Hamiltonian is the asymmetric form, expressed in terms of the relative displacements $\left\{R_{j}^{eq}\right\}$ between the PES minima.^105,137,139^ Assigning the equilibrium positions of state |1⟩ at the origin and shifting those of state |2⟩ accordingly for all modes, the Hamiltonian becomes

9 $$Ĥ={Ĥ}_{1}\left|1\right\rangle \left\langle 1\right|+{Ĥ}_{2}\left|2\right\rangle \left\langle 2\right|+\Gamma \left(\left|1\right\rangle \langle 2|+\left|2\right\rangle \langle 1|\right)$$

where the nuclear Hamiltonians are

$$\{\begin{array}{ll} {Ĥ}_{1} & =\overset{N}{\underset{j=1}{\sum }}\frac{{\mathrm{P̂}}_{j}^{2}}{2}+\overset{N}{\underset{j=1}{\sum }}\frac{1}{2}\omega_{j}^{2}{\mathrm{R̂}}_{j}^{2}-\Delta E,\\ {Ĥ}_{2} & =\overset{N}{\underset{j=1}{\sum }}\frac{{\mathrm{P̂}}_{j}^{2}}{2}+\overset{N}{\underset{j=1}{\sum }}\frac{1}{2}\omega_{j}^{2}{\left({\mathrm{R̂}}_{j}-R_{j}^{eq}\right)}^{2} \end{array}$$ 10

The displacement $R_{j}^{eq}$ along the *j*-th mode is related to the coupling coefficient via^105,106^

11 $$R_{j}^{eq}=\frac{2c_{j}}{\omega_{j}^{2}}=a_{j}\sqrt{E_{r}}$$

where

12 $$a_{j}=\sqrt{\frac{2}{N}}\frac{1}{\omega_{j}},\left(j=1,...,N\right)$$

The reorganization energy for the spin-boson model then reads

13 $$E_{r}=\overset{N}{\underset{j=1}{\sum }}\frac{2c_{j}^{2}}{\omega_{j}^{2}}=\overset{N}{\underset{j=1}{\sum }}\frac{1}{2}\omega_{j}^{2}{\left(R_{j}^{eq}\right)}^{2}$$

Substituting eq 11 into eq 3 yields the spectral density in terms of $\left\{R_{j}^{eq}\right\}$ :^106,120^

14 $$J\left(\omega \right)=\frac{\pi }{8}\overset{N}{\underset{j=1}{\sum }}\omega_{j}^{3}{\left(R_{j}^{eq}\right)}^{2}\delta \left(\omega -\omega_{j}\right)$$

From the above procedure for constructing the spin-boson model from realistic simulations, we emphasize that the reorganization energy is defined with respect to a *pair* of electronic states, |1⟩ and |2⟩, rather than being associated with a single electronic state (see Figure 1a). This property and the asymmetrical representation of the spin-boson model will play an important role in extending the model to the multistate scenario discussed in Section 3.

**Figure 1:**
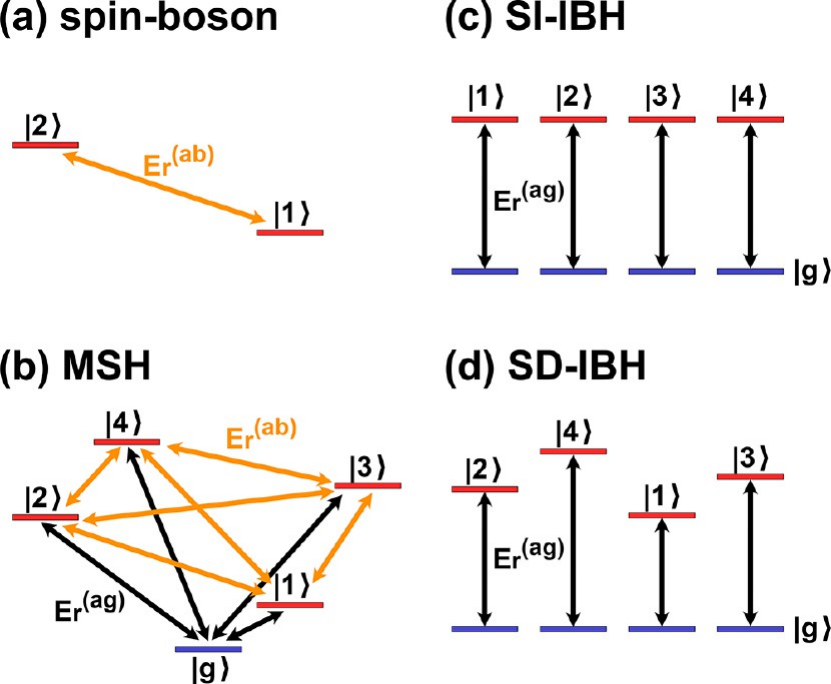
Schematic comparison of (a) spin-boson model, (b) multistate harmonic (MSH) model, (c) state-independent isolated bath harmonic (SI-IBH) model, and (d) state-dependent isolated bath harmonic (SD-IBH) model. The system-bath couplings are shown as double arrows that correspond to the reorganization energies between pairs of states, including those of the ground–excited state $E_{r}^{\left(ag\right)}$ (black) and excited–excited state $E_{r}^{\left(ab\right)}$ (orange). Note that in the spin-boson model, one of the states could be the ground state as well. In SI-IBH and SD-IBH models, the common ground state serves as a reference bath Hamiltonian, but the ground state |*g*⟩ does not have to be explicitly included as an electronic state, such as in the Frenkel exciton model.

### 2.2 Frenkel Exciton Model and the Isolated Bath Assumption

The general *F*-state Hamiltonian can be expressed in the diabatic electronic basis {|*a*⟩ | *a* = 1,···, *F*}, where |*a*⟩ corresponds to the *a*-th diabatic electronic state:

15 $$Ĥ=\left(\begin{array}{lllll} {Ĥ}_{1} & {\mathrm{V̂}}_{12} & {\mathrm{V̂}}_{13} & \cdots & {\mathrm{V̂}}_{1F}\\ {\mathrm{V̂}}_{21} & {Ĥ}_{2} & {\mathrm{V̂}}_{23} & \cdots & {\mathrm{V̂}}_{2F}\\ {\mathrm{V̂}}_{31} & {\mathrm{V̂}}_{32} & {Ĥ}_{3} & \cdots & {\mathrm{V̂}}_{3F}\\ \vdots & \vdots & \vdots & \ddots & \vdots \\ {\mathrm{V̂}}_{F1} & {\mathrm{V̂}}_{F2} & {\mathrm{V̂}}_{F3} & \cdots & {Ĥ}_{F} \end{array}\right)$$

Here, the diagonal blocks *Ĥ*_*a*_ (*a* = 1,···, *F*) are the nuclear Hamiltonians associated with the individual diabatic electronic states, and the off-diagonal blocks ${\mathrm{V̂}}_{ab}={\mathrm{V̂}}_{ba}^{†}\left(a\neq b\right)$ describe the electronic couplings between them.

A widely used multistate framework within this form is the *Frenkel exciton model*,^110^ which describes systems composed of *F* chromophoric sites. Each diabatic state |*a*⟩ represents a local electronic excitation (typically a singly excited state) on the *a*-th chromophore. A common simplifying assumption is that each chromophore interacts only with its own set of local nuclear modes, referred to as its *local bath bases*. Furthermore, if these local bath modes are statistically independent from one another, *isolated bath assumption* can be applied. This type of model, defined in the local bath bases, is called the *isolated bath harmonic* (IBH) model. For example, in a photosynthetic light-harvesting complex with *F* chromophores, each |*a*⟩ corresponds to an excitation localized on one site, and the associated bath describes intramolecular vibrations and local environmental fluctuations that modulate its energy.^121^ Note that using local bath bases does not automatically invoke the isolated bath assumption, since it is possible to write the correlated bath within the local bath bases, which will be discussed in Section 6.

Two common variants of the Frenkel exciton model are (i) the *state-independent* version in the conventional IBH form referred to as SI-IBH, in which all sites share the same local bath characteristics,^121,122,139,140^ and (ii) the more general *state-dependent* version referred to as SD-IBH, in which each site has distinct local bath parameters.^103,141–148^

In the state-dependent case, the nuclear Hamiltonian for state |*a*⟩ can be written as^143^

Ĥa=εa+∑a=1F∑j=1NP̂a,j22+Va(R̂)Va(R̂)=∑j=1N12ωa,j2(R̂a,j−ca,jωa,j2)2+∑b≠aF∑j=1N12ωb,j2R̂b,j2V̂ab=V̂ba†=Γab 16

Here, *N* is the number of local bath modes per site; the ε_*a*_ is the site energy; Γ_*ab*_ is the electronic coupling; **R̂** = {*R̂*_*a,j*_|*a* = 1,···,*F*;*j* = 1,···,*N*} and **P̂** = {*P̂*_*a,j*_|*a* = 1,···,*F*;*j* = 1,···,*N*} are the nuclear coordinates and momenta, where the coordinates and momenta of the nuclear modes coupled to the *a*-th locally excited site are *R̂*_*a*, *j*_ and *P̂*_*a*, *j*_ (*j* = 1,···, *N*). The parameters {ω_*a*, *j*_} and {*c*_*a*, *j*_} are the normal-mode frequencies of the ground state and the system–bath coupling strengths for the *a*-th site, respectively, and they are in general site-specific.

These parameters {ω_*a*, *j*_, *c*_*a*, *j*_} are determined by the site-specific spectral density,

17 $$J^{\left(ag\right)}\left(\omega \right)=\frac{\pi }{2}\overset{N}{\underset{j=1}{\sum }}\frac{c_{a,j}^{2}}{\omega_{a,j}}\delta \left(\omega -\omega_{a,j}\right)$$

which also defines the reorganization energy between the *a*-th excited state and its corresponding ground bath state (*g*):

18 $$E_{r}^{\left(ag\right)}=\frac{1}{\pi }\int_{0}^{\infty }\frac{J^{\left(ag\right)}\left(\omega \right)}{\omega }d\omega =\overset{N}{\underset{j=1}{\sum }}\frac{c_{a,j}^{2}}{2\omega_{a,j}^{2}}$$

The state-dependent system–bath coupling coefficients can then be expressed as

19 $$c_{a,j}=\sqrt{\frac{2E_{r}^{\left(ag\right)}}{N}}\omega_{a,j},\left(j=1,...,N\right)$$

Alternatively, the SD-IBH model in eqs 15 and 16 can also be written as the sum of the system, the bath, and the system-bath coupling parts:

20 Ĥ=Ĥsys+Ĥbath+ĤbsĤsys=∑a=1F(εa+Er(ag))|a⟩⟨a|+∑a≠bFΓab|a⟩⟨b|Ĥbath=∑a=1F∑j=1NP̂a,j22+12ωa,j2R̂a,j2Ĥbs=−∑a=1F∑j=1Nca,jR̂a,j|a⟩⟨a|

It is clear that the ground bath state characterized by *Ĥ*_bath_ does not correspond to an explicit electronic state, i.e., |*g*⟩, but *Ĥ*_bath_ serves as a reference to determine the system-bath coupling and reorganization energy between a singly excited state on a certain chromophore and the ground bath state.

In the state-independent Frenkel exciton (SI-IBH) model,^139,149,150^ all sites share the same spectral density profile, implying identical vibrational frequencies {ω_*j*_} and coupling coefficients {*c*_*j*_}. The *a*-th state’s PES is given by

21 $$V_{a}\left(\mathrm{R̂}\right)=\overset{N}{\underset{j=1}{\sum }}\frac{1}{2}\omega_{j}^{2}{\left({\mathrm{R̂}}_{a,j}-\frac{c_{j}}{\omega_{j}^{2}}\right)}^{2}+\overset{F}{\underset{b\neq a}{\sum }}\overset{N}{\underset{j=1}{\sum }}\frac{1}{2}\omega_{j}^{2}{\mathrm{R̂}}_{b,j}^{2}$$

For example, the standard Frenkel exciton model of Fenna–Metthews–Olson (FMO) photosynthetic light harvesting complex^111,114,121,143–147,151–175^ employs a Debye spectral density:^121^

22 $$J\left(\omega \right)=\frac{2\lambda \omega_{c}\omega }{\omega^{2}+\omega_{c}^{2}}$$

with the reorganization energy parameter λ = 35 cm^–1^ and the characteristic frequency ω_*c*_ = 106.14 cm^–1^ that corresponds to a bath correlation time τ_*c*_ = 50 fs.^176^

As illustrated schematically in Figure 1, Frenkel exciton (or IBH) models contain only locally excited states, each coupled to its own distinct set of local bath DOF. The electronic–nuclear coupling strengths may be identical across sites (state-independent case) or site-specific (state-dependent case), as indicated by the varying double-arrow lengths in Figure 1c,d, respectively. Importantly, in these models the system–bath couplings describe the nuclear reorganization between a locally excited state and its corresponding bath (or ground) state, rather than between different locally excited states.

Despite their wide use, Frenkel exciton and IBH models have several limitations when applied to nonadiabatic processes in the condensed phase. First, the isolated bath assumption may break down when chromophores are in close proximity, leading to correlated baths, or when multiple electronic states belong to the same molecule, in which case the bath is shared. Second, although the SD-IBH model can represent a certain degree of bath heterogeneity, it remains limited to describing the reorganization between a locally excited state and the ground bath state of the same molecular site, rather than between different sites. Third, the electronic states in IBH models are associated with individual molecular sites in a multichromophoric system, representing local rather than global states of the entire aggregate, which creates difficulties in describing states that involve multiple sites, such as CT or charge-separated states.

## 3 The Multistate Harmonic (MSH) Model: A Unified Framework for Heterogeneous Environment

To address the limitations inherent in the IBH model, particularly its inability to account for correlated environmental effects and its restrictive focus on local excitations, the MSH model was proposed in 2021.^120^ The MSH model offers a comprehensive and physically rigorous framework that generalizes the two-state spin-boson paradigm to an arbitrary number of electronic states. It systematically incorporates the complex network of interactions present in a realistic condensed-phase environment by ensuring that the model Hamiltonian is consistent with all pairwise reorganization energies derived from all-atom simulations. This is its defining feature and the key to its power.

The central challenge in constructing a general multistate model is to correctly capture the nuclear reorganization associated with every possible electronic transition. For an *F*-state system, there are *F*(*F* – 1)/2 such transitions, each with its own characteristic reorganization energy, $E_{r}^{\left(ab\right)}$ , which quantifies the energy cost for the environment to restructure as the system switches from electronic state |*a*⟩ to |*b*⟩. A standard model, which assigns a single, one-dimensional bath coordinate to each physical vibration, lacks the degrees of freedom to satisfy all these constraints simultaneously. The IBH model sidesteps this issue by only considering reorganization relative to a single reference (the ground state), but this is a physically incomplete picture.

The MSH model overcomes this fundamental limitation through a novel and geometrically intuitive solution: it extends the dimensionality of the nuclear coordinate space.^106,120^ For a system with *F* electronic states and *N* physical normal modes (e.g., the effective modes extracted from all-atom simulations of the entire system), the MSH model posits that each physical mode requires an (*F* – 1)-dimensional nuclear coordinate space to describe its coupling to the electronic system. The *F*(*F* – 1)/2 different reorganization energy restrictions are simultaneously satisfied in the MSH model as shown in Figure 1b. This dimensional extension is not a mathematical abstraction but a physical necessity to encode the full matrix of pairwise environmental correlations. The result is an effective Hamiltonian that is both general and rigorously connected to the underlying atomistic representation of a multistate system.

Let us first present the general MSH Hamiltonian before dissecting its construction with a more intuitive example. The MSH Hamiltonian adopts the same general electronic Hamiltonian form as eq 15, where the nuclear Hamiltonians for the *F* electronic states are given by^120^

{Ĥ1=∑a=1F−1∑j=1NP̂a,j22+∑j=1N12ωj2[R̂1,j2+R̂2,j2+⋯+R̂F−1,j2]+ε1,Ĥ2=∑a=1F−1∑j=1NP̂a,j22+∑j=1N12ωj2[(R̂1,j−Sj(12))2+R̂2,j2+⋯+R̂F−1,j2]+ε2,Ĥ3=∑a=1F−1∑j=1NP̂a,j22+∑j=1N12ωj2[(R̂1,j−Sj(13))2+(R̂2,j−Sj(23))2+⋯+R̂F−1,j2]+ε3,···ĤF=∑a=1F−1∑j=1NP̂a,j22+∑j=1N12ωj2[(R̂1,j−Sj(1F))2+(R̂2,j−Sj(2F))2+⋯+(R̂F−1,j−Sj(F−1,F))2]+εF 23

Here, {ε_*X*_|X = 1,···, *F*} are the potential energy minima of the *F* electronic states’s PESs, and the {ω_*j*_|*j* = 1,···,*N*} are the frequencies of the *N* physical normal modes of the entire system. The mass-weighted nuclear coordinates are {*R̂*_*a,j*_|*j* = 1,···,*N*;*a* = 1,···,*F* – 1} and the corresponding momenta are {*P̂*_*a,j*_|*j* = 1,···,*N*;*a* = 1,···,*F* – 1}, where the index *j* runs over the *N* physical modes, and the index *a* runs from 1 to *F* – 1, representing the extended nuclear dimensions. The total number of nuclear DOF is thus *N*_*n*_ = (*F* – 1) × *N*. The terms $\left\{S_{j}^{\left(aX\right)}|1\leq a<X\leq F\right\}$ are the displacement parameters that define the equilibrium geometry of each electronic state’s PES in this extended space, e.g., $S_{j}^{\left(aX\right)}$ denotes the equilibrium shift components for the *X*-th state’s PES along the *j*-th mode in the *a*-th nuclear subspace.

To understand the logic behind this seemingly complex structure, let us consider the simplest nontrivial case: a three-state system (*F* = 3). The goal is to construct a model that correctly reproduces the three pairwise reorganization energies, $E_{r}^{\left(12\right)}$ , $E_{r}^{\left(13\right)}$ , and $E_{r}^{\left(23\right)}$ , obtained from all-atom simulations.^106^ A conventional model with a single bath coordinate *R̂*_*j*_ for each physical mode *j* would define the PES for each state *X* as $V_{X}=\frac{1}{2}\overset{N}{\underset{j=1}{\sum }}\omega_{j}^{2}{\left({\mathrm{R̂}}_{j}-d_{j,X}\right)}^{2}+\varepsilon_{X}$ . If we set the minimum of state |1⟩ at the origin (*d*_*j*,1_ = 0), we can use the displacements *d*_*j*,2_ and *d*_*j*,3_ to match $E_{r}^{\left(12\right)}$ and $E_{r}^{\left(13\right)}$ , respectively. However, the reorganization energy between states |2⟩ and |3⟩, $E_{r}^{\left(23\right)}=\frac{1}{2}\overset{N}{\underset{j=1}{\sum }}\omega_{j}^{2}{\left(d_{j,2}-d_{j,3}\right)}^{2}$ , is then fixed by the geometry of the first two displacements. It is not an independent parameter and cannot be adjusted to match the value from an all-atom simulation, revealing the conventional model’s fundamental insufficiency. The underlying issue is the nuclear dimensions are not enough to represent each physical normal mode in a 3-state case. This motivated us to revisit how two-state spin-boson model was constructed: the displacement $R_{j}^{eq}$ in eq 11 of the asymmetric PESs in eq 10 actually reflect the reorganization between two states. In the two-state case, there is only one possible pair of states, thus the spin-boson model has only one set of normal modes defined in 1-dimensional nuclear space, as shown in Figure 2a.

**Figure 2:**
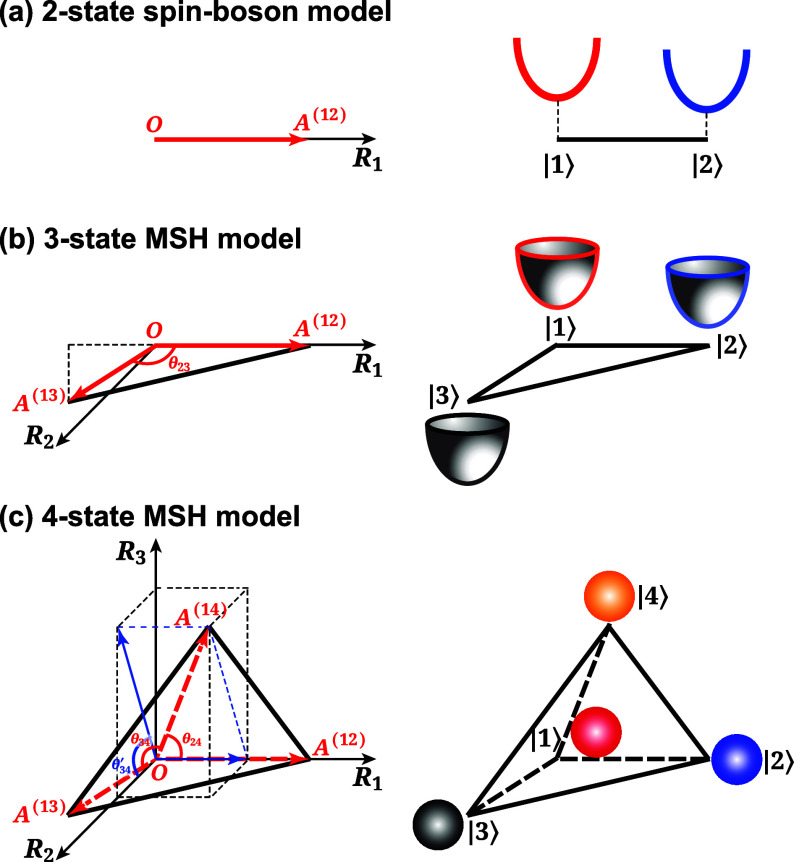
Geometric representation of the potential energy surface (PES) minima positions in two-state (spin-boson) (a), three-state (b), and four-state (c) MSH models. For each physical mode *j*, the PES minima of the electronic states (*O*,*A* ^(12)^), (*O*,*A* ^(12)^,*A* ^(13)^), and (*O*,*A* ^(12)^,*A* ^(13)^,*A* ^(14)^) are located in a one, two, and three-dimensional extended nuclear space spanned by coordinates *R̂*_1_, (*R̂*_1_,*R̂*_2_), and (*R̂*_1_,*R̂*_2_,*R̂*_3_), for the two-state, three-state, and four-state systems, respectively. The distances between the PES minima of states |*a*⟩ and |*b*⟩ are proportional to the square root of the corresponding reorganization energies, $\sqrt{E_{r}^{\left(ab\right)}}$ . The geometry of the resulting line, triangle, and tetrahedron is uniquely determined by the *F*(*F* – 1)/2 reorganization energies obtained with all-atom simulations, allowing the MSH model to satisfy all pairwise constraints simultaneously.

The MSH model solves this by expanding the nuclear space. For *F* = 3, we need an (*F* – 1) = 2-dimensional space for each physical mode *j*, spanned by coordinates *R̂*_1,*j*_,*R̂*_2,*j*_. The PES minima for the three electronic states are now verticies in this 2D space, as illustrated in Figure 2b. We can place the minimum of state |1⟩ at the origin, (0,0), without loss of generality. The minimum of state |2⟩ is then displaced along the first dimension, at a position $\left(S_{j}^{\left(12\right)},0\right)$ . Finally, the minimum of state |3⟩ is placed at a general position $\left(S_{j}^{\left(13\right)},S_{j}^{\left(23\right)}\right)$ .

The nuclear Hamiltonians for this 3-state system are^106^

24 {Ĥ1=12∑j=1N(P̂1,j2+P̂2,j2)+∑j=1N12ωj2(R̂1,j2+R̂2,j2)+ε1,Ĥ2=12∑j=1N(P̂1,j2+P̂2,j2)+∑j=1N12ωj2[(R̂1,j−Sj(12))2+R̂2,j2]+ε2,Ĥ3=12∑j=1N(P̂1,j2+P̂2,j2)+∑j=1N12ωj2[(R̂1,j−Sj(13))2+(R̂2,j−Sj(23))2]+ε3

The reorganization energy between any two states, |*X*⟩ and |*Y*⟩, is defined as the energy required to go from the minimum of *V*_*X*_ to the minimum of *V*_*Y*_ on the PES of state *X* (or *Y*). This corresponds to the sum over all modes *j* of one-half the squared Euclidean distance between the state minima in the extended space, multiplied by $\omega_{j}^{2}$ as in eq 13. For our 3-state example, these are given by

25 $$E_{r}^{\left(12\right)}=\overset{N}{\underset{j=1}{\sum }}\frac{1}{2}\omega_{j}^{2}{\left(S_{j}^{\left(12\right)}\right)}^{2}$$

26 $$E_{r}^{\left(13\right)}=\overset{N}{\underset{j=1}{\sum }}\frac{1}{2}\omega_{j}^{2}\left[{\left(S_{j}^{\left(13\right)}\right)}^{2}+{\left(S_{j}^{\left(23\right)}\right)}^{2}\right]$$

27 $$E_{r}^{\left(23\right)}=\overset{N}{\underset{j=1}{\sum }}\frac{1}{2}\omega_{j}^{2}\left[{\left(S_{j}^{\left(13\right)}-S_{j}^{\left(12\right)}\right)}^{2}+{\left(S_{j}^{\left(23\right)}\right)}^{2}\right]$$

This constitutes a system of three equations for the three sets of displacement parameters $\left\{S_{j}^{\left(12\right)}\right\},\{S_{j}^{\left(13\right)}\},$ and $\left\{S_{j}^{\left(23\right)}\right\}$ . These equations can be solved systematically to determine the displacements from the all-atom reorganization energies, ensuring the model’s consistency with the underlying physics. Geometrically, the three state minima form a triangle whose side lengths are proportional to the square root of the corresponding reorganization energies. The extended 2D space provides the necessary freedom to construct a triangle with any three specified side lengths, provided they satisfy the triangle inequality, a condition that is physically guaranteed.

The logic extends seamlessly to systems with more states. For a four-state system (*F* = 4), there are *F*(*F* – 1)/2 = 6 pairwise reorganization energies $\left(E_{r}^{\left(12\right)},E_{r}^{\left(13\right)},E_{r}^{\left(14\right)},E_{r}^{\left(23\right)},E_{r}^{\left(24\right)},E_{r}^{\left(34\right)}\right)$ that the model must satisfy. To accommodate these constraints, the MSH model requires an (*F* – 1) = 3-dimensional extended nuclear space for each physical mode *j*, spanned by the coordinates (*R̂*_1,*j*_,*R̂*_2,*j*_,*R̂*_3,*j*_). The PES minima of the four electronic states now form a tetrahedron in this 3D space, as depicted in Figure 2c. The Hamiltonian for the four-state MSH model takes the form:^120^

{Ĥ1=∑a=13∑j=1NP̂a,j22+∑j=1N12ωj2(R̂1,j2+R̂2,j2+R̂3,j2)+ε1,Ĥ2=∑a=13∑j=1NP̂a,j22+∑j=1N12ωj2[(R̂1,j−Sj(12))2+R̂2,j2+R̂3,j2]+ε2,Ĥ3=∑a=13∑j=1NP̂a,j22+∑j=1N12ωj2[(R̂1,j−Sj(13))2+(R̂2,j−Sj(23))2+R̂3,j2]+ε3,Ĥ4=∑a=13∑j=1NP̂a,j22+∑j=1N12ωj2[(R̂1,j−Sj(14))2+(R̂2,j−Sj(24))2+(R̂3,j−Sj(34))2]+ε4 28

The six edge lengths of the tetrahedron are determined by the six reorganization energies, and the displacement parameters $\left\{S_{j}^{\left(aX\right)}\right\}$ are solved to construct this geometry. This hierarchical construction, moving from a line for two states, to a triangle for three, to a tetrahedron for four (Figure 2), illustrates the elegant geometric foundation of the MSH model and its capacity to systematically encode the full matrix of environmental correlations for any number of states.

This logic generalizes directly to the *F*-state case presented in eq 23. To satisfy the *F*(*F* – 1)/2 pairwise reorganization energies, an (*F* – 1)-dimensional extended nuclear space is required for each physical mode. The PES minima of the *F* electronic states form a simplex in this space. The position of each state’s minimum is defined by the displacement vectors in eq 23, which are systematically chosen such that the squared distance between any two minima, summed over all modes, correctly reproduces the corresponding reorganization energy $E_{r}^{\left(XY\right)}$ from the all-atom data. The MSH model, therefore, provides a mathematically complete and physically motivated prescription for building an effective Hamiltonian that captures the full matrix of electronic-vibrational couplings in a multistate quantum system.

To determine the equilibrium shift component parameters, $\left\{S_{j}^{\left(aX\right)}\right\}$ , we need to obtain the *F*(*F* – 1)/2 energy-gap TCFs from all-atom simulations:

29 $$\begin{array}{lllll} C_{UU}^{\left(12\right)}\left(t\right) & & & \\ C_{UU}^{\left(13\right)}\left(t\right), & C_{UU}^{\left(23\right)}\left(t\right) & & \\ C_{UU}^{\left(14\right)}\left(t\right), & C_{UU}^{\left(24\right)}\left(t\right), & C_{UU}^{\left(34\right)}\left(t\right) & \\ \vdots & \vdots & \vdots & \ddots \\ C_{UU}^{\left(1F\right)}\left(t\right), & C_{UU}^{\left(2F\right)}\left(t\right), & C_{UU}^{\left(3F\right)}\left(t\right), & \cdots & C_{UU}^{\left((F-1)F\right)}\left(t\right) \end{array}$$

where the TCF of the energy gap between the PESs of states *X* and *Y* are defined as

30 $$C_{UU}^{\left(XY\right)}\left(t\right)=\left\langle U_{XY}\left(t\right)U_{XY}\left(0\right)\right\rangle -\langle U_{XY}\rangle^{2}$$

Here, the energy gap is *U*_*XY*_ = *V*_*X*_ – *V*_*Y*_, where *V*_*X*_ and *V*_*Y*_ denote the PESs of the distinct states *X* and state *Y*. From these TCFs, we can calculate their corresponding spectral density *J* ^*(XY)*^(ω) and reorganization energies $\left\{E_{r}^{\left(XY\right)}\right\}$ for all *F*(*F* – 1)/2 pairs of states using

31 $$J^{\left(XY\right)}\left(\omega \right)=\frac{\beta \omega }{4}\int_{0}^{\infty }dtC_{UU}^{\left(XY\right)}\left(t\right)\cos\left(\omega t\right)$$

32 $$E_{r}^{\left(XY\right)}=\frac{C_{UU}^{\left(XY\right)}\left(0\right)}{2k_{B}T}$$

The normal-mode frequency set {ω_*j*_} is assumed to be the same for all states as they represent the same global nuclear motions (a globally shared bath). This set can be obtained from any one of the $C_{UU}^{\left(XY\right)}\left(t\right)$ (e.g., $C_{UU}^{\left(12\right)}\left(t\right)$ ) using eq 6. These reorganization energies serve as the fundamental constraints that the model must satisfy.^120^

The core of the parametrization is to solve a geometric problem for each of the *N* physical modes of the system. For a given mode *j* with frequency ω_*j*_, the minima of the *F* electronic states are treated as vertices of a simplex (e.g., a triangle for *F* = 3, a tetrahedron for *F* = 4) residing in an (*F* – 1)-dimensional Cartesian space. The length of the edge connecting any two vertices *X* and *Y* in this space, denoted as $R_{j}^{\left(XY\right)}$ , is directly proportional to the square root of the reorganization energy $\sqrt{E_{r}^{\left(XY\right)}}$ :

33 $$R_{j}^{\left(XY\right)}=\sqrt{\frac{2E_{r}^{\left(XY\right)}}{N}}\frac{1}{\omega_{j}}=a_{j}\sqrt{E_{r}^{\left(XY\right)}},\left(j=1,...,N\right)$$

where $a_{j}=\sqrt{2/\left(N\omega_{j}^{2}\right)}$ is a mode-dependent scaling factor. The reorganization energy can in turn be expressed as a sum over these modal displacements:

34 $$E_{r}^{\left(XY\right)}=\overset{N}{\underset{j=1}{\sum }}\frac{1}{2}\omega_{j}^{2}{\left(R_{j}^{\left(XY\right)}\right)}^{2}$$

The problem then becomes one of determining the coordinates of these *F* vertices given all the *F*(*F* – 1)/2 intervertex distances for each mode.

This is achieved using a procedure equivalent to Gram–Schmidt orthogonalization,^177,178^ where the vertices are placed sequentially into the (*F* – 1)-dimensional space. The geometry of the simplex is defined by the angles between the vectors connecting the vertices, where the vector between PES minima of |*i*⟩ and |*k*⟩ has length $\left|v_{ik}\right|=v_{ik}=\sqrt{E_{r}^{\left(ik\right)}},\left(i\neq k\right)$ . For instance, if we place state |1⟩ at the origin, the angle θ_*ik*_ between the vectors pointing from the origin to state |*i*⟩ (**v**_1i_) and to state |*k*⟩ (**v**_1*k*_) can be found from the law of cosines using the known edge lengths:

35 $$\cos\theta_{ik}=\frac{E_{r}^{\left(1i\right)}+E_{r}^{\left(1k\right)}-E_{r}^{\left(ik\right)}}{2\sqrt{E_{r}^{\left(1i\right)}E_{r}^{\left(1k\right)}}}$$

The algorithmic construction for each mode *j* is as follows:

1 Place state |1⟩ at the origin of the (*F* – 1)-dimensional space.
2 Place state |2⟩ on the first axis at a distance of $R_{j}^{\left(12\right)}$ (see Figure 2a).
3 Place state |3⟩ in the plane defined by the first two axes. Its coordinates are determined by its distances $R_{j}^{\left(13\right)}$ and $R_{j}^{\left(23\right)}$ from the first two states, which uniquely defines its position via the angle θ_23_ (see Figure 2b).
4 Place the fourth state, |4⟩, in the 3D space defined by the first three axes. Its position is fixed by its distances to the first three states, which requires calculating the angles θ_24_, θ_34_, and a projected angle $\theta_{34}^{\prime }$ that orients it correctly with respect to the plane formed by the first three states (see Figure 2c).
5 This process continues for all *F* states. Each new state |*k*⟩ is positioned in the (*k* – 1)-dimensional space using its distances to all previously placed states, {1,2,···,*k* – 1}. This involves calculating the angles θ_*ik*_ for *i* = 2,···,*k* – 1 and a series of projected angles $\theta_{ik}^{\prime }$ (the angles between the projected **v**_1*i*_ and **v**_1*k*_), which determine the coordinates in each dimension of the orthonormal basis.

This sequential orthogonalization procedure generates the equilibrium shift components $\left\{S_{j}^{\left(aX\right)}\right\}$ of the MSH model, which are proportional to the Cartesian coordinates of the PES minimum of each state, and they form a lower triangle matrix as below (here *D* = *F* − 1)

36 $$\left(\begin{array}{llllll} S_{j}^{\left(12\right)} & & \\ S_{j}^{\left(13\right)} & S_{j}^{\left(23\right)} & \\ S_{j}^{\left(14\right)} & S_{j}^{\left(24\right)} & S_{j}^{\left(34\right)}\\ S_{j}^{\left(15\right)} & S_{j}^{\left(25\right)} & S_{j}^{\left(35\right)} & S_{j}^{\left(45\right)}\\ \vdots & \vdots & \vdots & \vdots & \ddots \\ S_{j}^{\left(1D\right)} & S_{j}^{\left(2D\right)} & S_{j}^{\left(3D\right)} & S_{j}^{\left(4D\right)} & \cdots & S_{j}^{\left(D-1,D\right)} \end{array}\right)$$

The PES minima of the *F* states defined in the (*F* – 1)-dimensional extended space are described in Appendix A.

For example, the equilibrium shift components in the 3-state case are

37 $$\begin{array}{ll} S_{j}^{\left(12\right)} & =a_{j}\sqrt{E_{r}^{\left(12\right)}},\\ S_{j}^{\left(13\right)} & =a_{j}\sqrt{E_{r}^{\left(13\right)}}\cos\theta_{23}\\ S_{j}^{\left(23\right)} & =a_{j}\sqrt{E_{r}^{\left(13\right)}}\sin\theta_{23} \end{array}$$

and those in the 4-state case are

38 $$\begin{array}{ll} S_{j}^{\left(12\right)} & =a_{j}\sqrt{E_{r}^{\left(12\right)}}\\ S_{j}^{\left(13\right)} & =a_{j}\sqrt{E_{r}^{\left(13\right)}}\cos\theta_{23}\\ S_{j}^{\left(23\right)} & =a_{j}\sqrt{E_{r}^{\left(13\right)}}\sin\theta_{23}\\ S_{j}^{\left(14\right)} & =a_{j}\sqrt{E_{r}^{\left(14\right)}}\cos\theta_{24}\\ S_{j}^{\left(24\right)} & =a_{j}\sqrt{E_{r}^{\left(14\right)}}\sin\theta_{24}\cos\theta_{34}^{\prime }\\ S_{j}^{\left(34\right)} & =a_{j}\sqrt{E_{r}^{\left(14\right)}}\sin\theta_{24}\sin\theta_{34}^{\prime } \end{array}$$

Here, $\cos\theta_{3k}^{\prime }\left(k\geq 4\right)$ is defined in eq 137. One can readily verify that this construction reproduces the input reorganization energies. For instance, the squared distance between states |2⟩ and |3⟩ in the 3-state MSH model is

39 $$\begin{array}{ll} & \overset{N}{\underset{j=1}{\sum }}\frac{1}{2}\omega_{j}^{2}\left[{\left(S_{j}^{\left(13\right)}-S_{j}^{\left(12\right)}\right)}^{2}+{\left(S_{j}^{\left(23\right)}\right)}^{2}\right]\\ & =\overset{N}{\underset{j=1}{\sum }}\frac{1}{2}\omega_{j}^{2}a_{j}^{2}\left[{\left(\sqrt{E_{r}^{\left(13\right)}}\cos\theta_{23}-\sqrt{E_{r}^{\left(12\right)}}\right)}^{2}+{\left(\sqrt{E_{r}^{\left(13\right)}}\sin\theta_{23}\right)}^{2}\right]\\ & =\overset{N}{\underset{j=1}{\sum }}\frac{1}{N}\left[E_{r}^{\left(13\right)}+E_{r}^{\left(12\right)}-2\sqrt{E_{r}^{\left(12\right)}E_{r}^{\left(13\right)}}\cos\theta_{23}\right]\\ & =\overset{N}{\underset{j=1}{\sum }}\frac{1}{N}\left[E_{r}^{\left(13\right)}+E_{r}^{\left(12\right)}-\left(E_{r}^{\left(12\right)}+E_{r}^{\left(13\right)}-E_{r}^{\left(23\right)}\right)\right]=E_{r}^{\left(23\right)} \end{array}$$

This confirms that the geometry correctly encodes the third reorganization energy, $E_{r}^{\left(23\right)}$ , which is impossible within 1D nuclear space, if the PES minima of the third state are located at distances from those of the first state determined by eq 33. Furthermore, eq 35 is not restrictive to state |1⟩ and other states, and more generally, the reorganization energies for pairs from any three states, e.g., |*a*⟩, |*b*⟩, and |*c*⟩ ∈ {1,···,*F*}, should satisfy

40 $$E_{r}^{\left(ab\right)}=E_{r}^{\left(ac\right)}+E_{r}^{\left(bc\right)}-2\sqrt{E_{r}^{\left(ac\right)}E_{r}^{\left(bc\right)}}\cos\theta_{ab}$$

The above triangular reorganization energy relation for the three-state case was first proposed by Cho and Silbey.^113^

Finally, the potential energy minima parameters {ϵ_*X*_} can be obtained by electronic structure calculations given the optimized equilibrium geometries of all states or by MD simulations that generate the reaction free energies Δ*E* ^*(XY)*^:

41 $$\varepsilon_{Y}-\varepsilon_{X}=\Delta E^{\left(XY\right)}=-E_{r}^{\left(XY\right)}-\left\langle U_{XY}\right\rangle$$

The electronic couplings {Γ_*XY*_} can be computed using the fragment charge difference (FCD),^179^ fragment energy difference (FED),^180^ generalized Mulliken-Hush (GMH),^181–183^ or Boys localization^184^ methods. Thus, all parameters of the general *F*-state MSH model Hamiltonian are constructed from all-atom simulations.

The MSH model, as detailed above, is defined by a set of features that distinguish it from traditional frameworks. It provides a more physically rigorous description of nonadiabatic dynamics by treating all electronic states on an equal footing, including the ground state, rather than focusing solely on excited-state manifolds. The model’s primary strength is its ability to describe heterogeneous and correlated environments, which stems from its construction: it is designed to systematically satisfy all *F*(*F* – 1)/2 pairwise reorganization energies derived from all-atom simulations. As this section has shown, this consistency is achieved by representing each physical mode in an extended (*F* – 1)-dimensional space. This representation allows the PES minima to form a polyhedron whose geometry (depicted in Figure 2) directly encodes the full network of state-to-state correlations. This framework is also general: it naturally describes uncorrelated, isolated baths in the specific case where all-atom data indicate geometrically orthogonal PES minima. Furthermore, the model’s parameters are physically grounded in the dynamics of realistic condensed-phase system. Because the spectral densities are constructed from all-atom simulations, they are consequently dominated by the low-frequency intermolecular vibrational modes (typically below 250 cm^–1^) that govern solvation and protein dynamics, a key distinction from models developed for gas-phase molecules. Finally, it is essential to clarify the two forms of coupling within the MSH Hamiltonian. The electronic-vibrational (system-bath) couplings are diagonal terms, characterized by the reorganization energies, which manifest as the equilibrium shifts between the PESs for each state. These are distinct from the off-diagonal electronic couplings, which represent the direct mixing between diabatic states and can be treated as constant (the Condon approximation) or as coordinate-dependent to include non-Condon effects.

The ultimate check of an effective model’s validity, however, goes beyond its ability to reproduce static properties like reorganization energies. The true measure of its success lies in its ability to reproduce the *nonadiabatic dynamics* predicted by the full, all-atom multistate Hamiltonian it seeks to represent. This comparison serves as the most stringent benchmark, directly testing the physical assumptions embedded within the model. A model that is parametrized from atomistic data but fails to replicate the resulting temporal evolution of the system is of limited practical use. Historically, this crucial validation step has often been challenging to perform, partly due to the significant difficulty of developing and applying all-atom nonadiabatic dynamics methods to complex condensed-phase systems. Furthermore, the conventional perspective has sometimes treated model building as a one-way process focused on parameter extraction, without always asking the subsequent question of whether the resulting model is dynamically accurate.

We have performed such a critical test for the PICT dynamics of CPC_60_ molecular triad dissolved in explicit tetrahydrofuran (THF) solvent.^119,134^ This system is a prototypical system for photovoltaics, designed to undergo ultrafast charge transfer upon photoexcitation. The process begins with the absorption of a photon, which prepares the system in a local excitonic state on the porphyrin moiety, denoted as the ππ* state. Following this initial excitation, the system evolves through a network of nonadiabatic transitions involving two distinct CT states: a partially charge-transfer state (CT1) and a fully charge-transfer state (CT2). This interplay of multiple competing charge transfer pathways is sensitive to the subtle fluctuations of the surrounding solvent environment, making it an ideal test case for multistate Hamiltonian models. As shown in the first two columns of Figure 3, the spectral densities of all pairs of electronic states in the triad have a rather similar structured spectrum (except for the scaling constant that is relevant to reorganization energy), which support our assumption that the same set of normal-mode frequencies could be used for different nuclear subspaces. Another important feature of the spectral density for condensed-phase systems is that the predominant low-frequency modes (less than 400 cm^–1^) have much heavier weights for the reorganization energy than the high-frequency modes (larger than 400 cm^–1^). The low-frequency modes correspond to the intermolecular motions that involve multiple solute and solvent molecules, some of which are collective ones.

**Figure 3:**
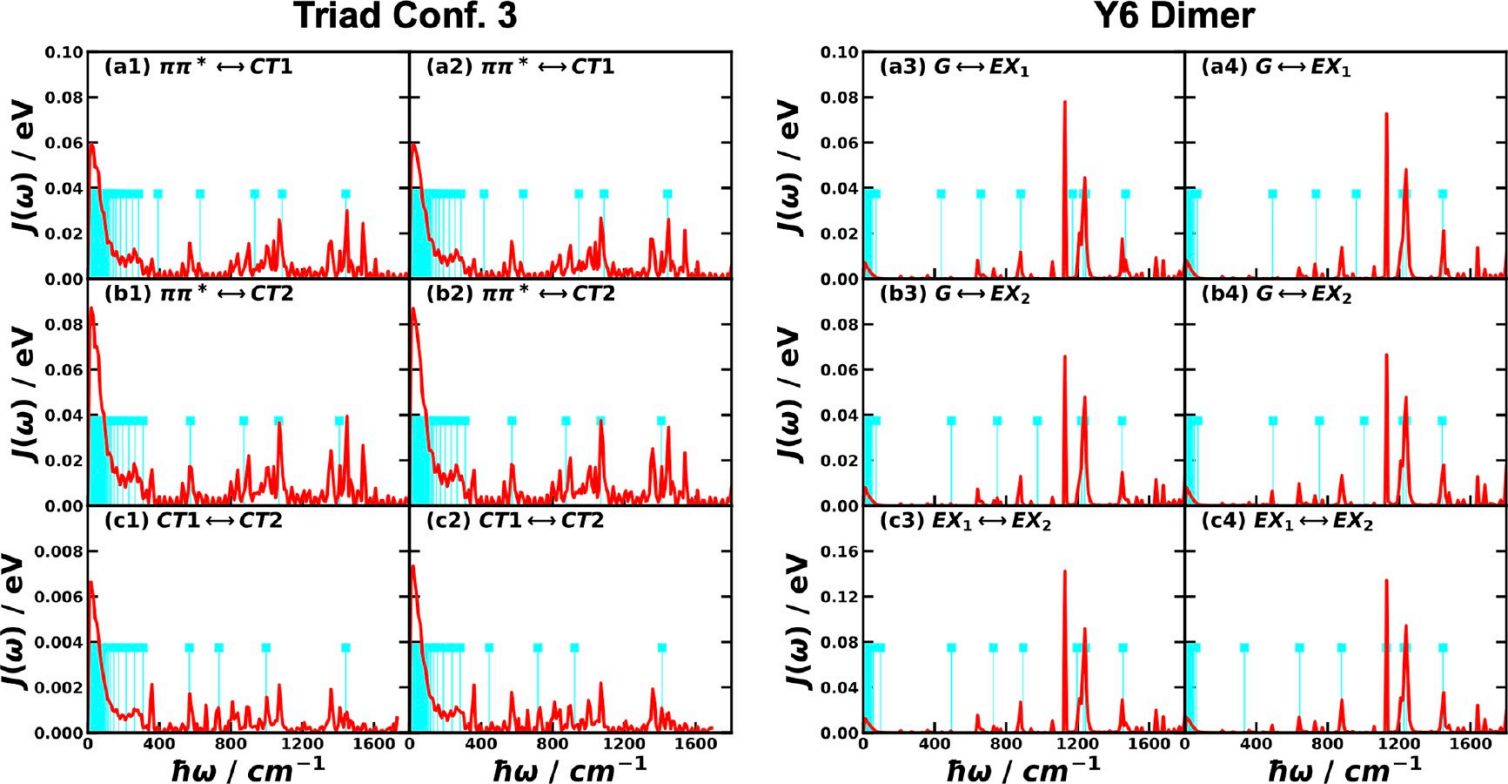
Spectral densities for the different pairs of electronic states obtained with all-atom MD simulations for CPC_60_ triad conformation 3 in THF solution^105^ and Y6 dimer separated by 10 Å in chloroform solution at 300 K. Column 1 is based the energy-gap TCFs sampled on ππ* state and column 2 is on the ground state of the triad; column 3 is based on sampling on EX_1_ state, and column 4 is on the ground state of the Y6 dimer.

To validate the MSH model, we compared the nonadiabatic dynamics it produced against reference results from the full, all-atom Hamiltonian, employing a wide array of state-of-the-art semiclassical and mixed quantum-classical simulation methods. As shown in Figure 4a,b, we showed the population dynamics of the two key excited states (ππ* and CT2) using methods including the linearized semiclassical approach^119,120,150,185–198^ (LSC1 ^186^ and LSC2,^185^ its resolution-of-identity variants (RI-LSC1–3),^195–198^ and symmetrical quasiclassical (SQC) method^139,199–206^ with the Meyer-Miller-Stock-Thoss (MMST) mapping Hamiltonian,^207,208^ as well as mean-field Ehrenfest theory,^209,210^ classical mapping model (CMM),^211–216^ and spin-mapping model (SPM).^217,218^ These comparisons are also plotted separately for each dynamical method in Figure S1 in the Supporting Information. While the absolute population dynamics differ between the various nonadiabatic dynamical methods, reflecting their inherent theoretical approximations, a striking consistency emerges: for any given dynamical method, the results from the MSH model are in excellent agreement with those from the all-atom benchmark. This remarkable correspondence demonstrates that the MSH model faithfully captures the essential physics of the system-bath interactions that govern the quantum dynamics, regardless of the specific algorithm used to propagate the equations of motion.

**Figure 4:**
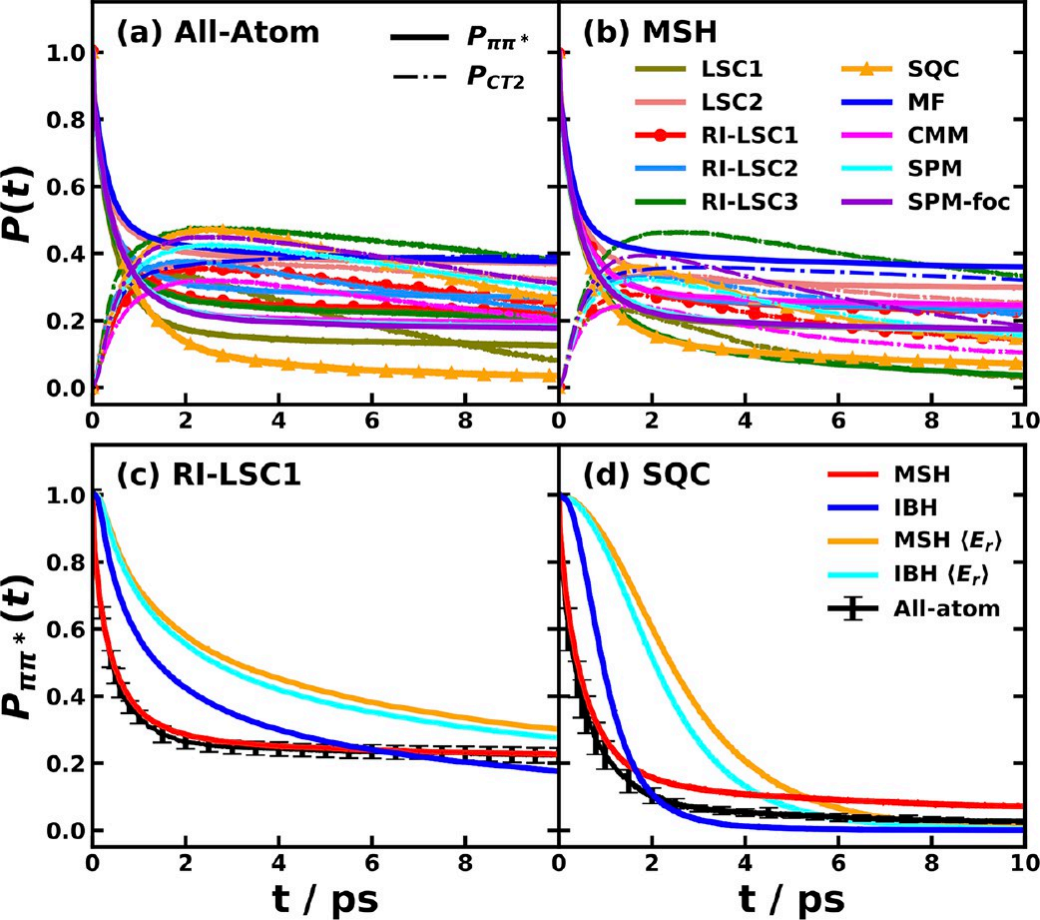
Comparison of nonadiabatic dynamics for the CPC_60_ triad conformation 3 in THF at 300 K, simulated with various Hamiltonians and dynamical methods. Panels (a) and (b) show the population dynamics of the ππ* (solid line) and CT2 (dash-dot line) states calculated using the all-atom multistate Hamiltonian (a) and the MSH model (b).^119^ The different colors correspond to different semiclassical and mixed quantum-classical dynamics methods. Panels (c) and (d) show the ππ* population dynamics calculated with the all-atom Hamiltonian (black with error bars) and various models using the RI-LSC1 (c) and SQC with triangle window (d) methods, reproduced from ref 134 with permission of AIP Publishing. The models shown are the original MSH model with heterogeneous reorganization energies (red), the state-dependent IBH model (blue), the MSH model with averaged reorganization energy ⟨*E*_*r*_⟩ (orange), and the state-independent IBH model with averaged reorganization energy ⟨*E*_*r*_⟩ (cyan).

The effectiveness of the MSH framework is further highlighted when contrasted with conventional Frenkel exciton or IBH models. In Figure 4c,d, we compare the decay of the initially populated ππ* state as computed with the all-atom Hamiltonian, the two versions of the MSH models with the original heterogeneous *E*_*r*_ and with the averaged ⟨*E*_*r*_⟩, and two versions of the IBH model with state-dependent *E*_*r*_ and with state-independent ⟨*E*_*r*_⟩, using the RI-LSC1 and SQC methods, respectively.^134^ The results are unambiguous. The MSH model’s predictions substantially agree with the all-atom benchmark dynamics. In stark contrast, both the standard SI-IBH model and the more flexible SD-IBH variant fail to capture the correct dynamics, predicting a slower decay of the excitonic state, even starting from the very early stage; the SI-IBH is much slower than the SD-IBH. The MSH with state-independent averaged ⟨*E*_*r*_⟩ is seen to be the slowest among all model Hamiltonians considered here, which could be attributed to the inability to represent the important heterogeneous environment for different electronic states. This result provides compelling evidence that for PICT that occurs among multiple electronic states on a chromophore embedded in a condensed-phase environment, the bath is intrinsically correlated and heterogeneous. The isolated bath assumption is not merely a minor approximation but a fundamental misrepresentation of the physics, and the MSH framework’s rigorous inclusion of these system-bath correlations is essential for achieving predictive accuracy. We will show more results for the importance of globally shared bath and the versatility of the MSH framework for representing heterogeneous environments and even for the limiting case of uncorrelated environments in Section 5.

## 4 The Multistate Reaction Coordinate (MRC) Model: An Equivalent and Physically Intuitive Representation

While the MSH model provides a formally complete framework for multistate nonadiabatic dynamics, its representation in terms of delocalized normal modes can obscure the intuitive physical picture of a collective reaction coordinate driving the process. To restore this picture, the MRC model was developed as an exactly equivalent representation of the MSH Hamiltonian.^135^ The MRC model transforms the high-dimensional bath of the MSH model into a more intuitive structure composed of a few primary reaction coordinates (RCs) that are directly coupled to the electronic states, and a set of secondary bath modes that are, in turn, coupled to the primary RCs.^219^

The motivation for this transformation stems from the long history of using RC models, like the single-state Caldeira-Leggett (CL)^104,220–223^ and the two-state Garg-Onuchic-Ambegaokar (GOA),^138,224,225^ Hamiltonians, to provide a clear physical description of nonadiabatic dynamics. The CL Hamiltonian consists of a primary reaction coordinate, *ŷ*, subject to an arbitrary potential *V*_0_(*ŷ*) and a collection of secondary harmonic bath modes, {*x̂*_α_|α = 1,···,*N* – 1}, and the primary mode and the secondary bath modes are bilinearly coupled:

42 $${Ĥ}_{\mathrm{CL}}=\frac{{\mathrm{P̂}}_{y}^{2}}{2}+V_{0}\left(ŷ\right)+\overset{N-1}{\underset{\alpha =1}{\sum }}\left[\frac{{\mathrm{p̂}}_{\alpha }^{2}}{2}+\frac{1}{2}{\mathrm{\omega ̃}}_{\alpha }^{2}{\left({\mathrm{x̂}}_{\alpha }+\frac{{\mathrm{c̃}}_{\alpha }}{{\mathrm{\omega ̃}}_{\alpha }^{2}}ŷ\right)}^{2}\right]$$

The GOA model was designed for nonadiabatic transition between two electronic states, where the primary RC, *ŷ*, on the two states is subject to shifted harmonic potentials with the same characteristic frequency Ω, and it connects the electronic DOF and the secondary bath modes {*x̂*_α_|α = 1,···,*N* – 1} associated with frequencies {*ω̃*_α_|α = 1,···, *N* – 1}:

43 $$\begin{array}{ll} {Ĥ}_{\mathrm{GOA}} & =\varepsilon {\mathrm{\sigma ̂}}_{z}+\Gamma {\mathrm{\sigma ̂}}_{x}+\frac{{\mathrm{P̂}}_{y}^{2}}{2}+\frac{1}{2}\Omega^{2}{\left(ŷ+y_{0}{\mathrm{\sigma ̂}}_{z}\right)}^{2}\\ & +\overset{N-1}{\underset{\alpha =1}{\sum }}\left[\frac{{\mathrm{p̂}}_{\alpha }^{2}}{2}+\frac{1}{2}{\mathrm{\omega ̃}}_{\alpha }^{2}{\left({\mathrm{x̂}}_{\alpha }+\frac{{\mathrm{c̃}}_{\alpha }}{{\mathrm{\omega ̃}}_{\alpha }^{2}}ŷ\right)}^{2}\right] \end{array}$$

Here, {*c̃*_α_|α = 1,···,*N* – 1} denote the coupling coefficients between the primary RC mode and the secondary bath modes, which should be distinguished from the electronic-nuclear couplings {*c*_*j*_|*j* = 1,···,*N*} for all normal modes of the spin-boson model in eq 1. As shown in eq 43, only the primary RC has an equilibrium shift between the two states, i.e., 2*y*_0_, such that the reorganization energy is given by $E_{r}=2\Omega^{2}y_{0}^{2}$ , and the secondary bath modes are not shifted across the two states. The spectral density of the secondary bath is defined similarly as that of the normal modes (eq 3):

44 $$J_{\mathrm{bath}}\left(\omega \right)=\frac{\pi }{2}\overset{N-1}{\underset{\alpha =1}{\sum }}\frac{{\mathrm{c̃}}_{\alpha }^{2}}{{\mathrm{\omega ̃}}_{\alpha }}\delta \left(\omega -{\mathrm{\omega ̃}}_{\alpha }\right)$$

When the spectral density of the secondary bath is assumed to be the Ohmic form, $J_{\mathrm{Ohm}}\left(\omega \right)=\eta \omega e^{-\omega /\omega_{c}}$ (η is the friction coefficient and ω_*c*_ is the cutoff frequency), at the limit of Ω ≪ ω_*c*_, an asymptotic effective spectral density for the corresponding normal modes was proposed by Garg et al.^224^

45 $$J_{\mathrm{eff}}^{\left(1\right)}\left(\omega \right)=\frac{\eta \omega \Omega^{4}y_{0}^{2}}{{\left(\Omega^{2}-\omega^{2}\right)}^{2}+\eta^{2}\omega^{2}}$$

which can be derived by following the Leggett approach:^220^

Jeff(ω)=limε→0+Im[K(ω−iε)] 46

47 $$K\left(z\right)=-z^{2}+\Omega^{2}y_{0}^{2}\frac{L\left(z\right)}{L\left(z\right)+\Omega^{2}}$$

48 $$L\left(z\right)=-z^{2}\left[1+\frac{2}{\pi }\int_{0}^{\infty }d\omega^{\prime }\frac{J_{\mathrm{Ohm}}\left(\omega^{\prime }\right)}{\omega^{\prime }\left(\omega^{\prime 2}-z^{2}\right)}\right]$$

where ω, ω′, and ε are real, and *z* is complex. However, the effective spectral density $J_{\mathrm{eff}}^{\left(1\right)}\left(\omega \right)$ in some parameter regions might be inaccurate, e.g., a significant deviation in the Fermi’s golden rule rate constant between the spin-boson model with numerically accurate discrete normal modes (eq 3) and the effective spectral density (eq 45) is seen.^138^ Thus, the effective spectral density in eq 45 should be used with caution, and it is advised to check against the discrete normal modes.

The advantage of the GOA model over the spin-boson model is a clear physical picture provided by the RC. This primary/secondary mode representation can be transformed into the standard normal-mode picture of the spin-boson model by diagonalizing the corresponding Hessian matrix of the nuclear PES:

49 $$D=\left(\begin{array}{ccccc} \Omega^{2}+\underset{\alpha }{\sum }\frac{{\mathrm{c̃}}_{\alpha }^{2}}{{\mathrm{\omega ̃}}_{\alpha }^{2}} & {\mathrm{c̃}}_{1} & {\mathrm{c̃}}_{2} & \cdots & {\mathrm{c̃}}_{N-1}\\ {\mathrm{c̃}}_{1} & {\mathrm{\omega ̃}}_{1}^{2} & & & \\ {\mathrm{c̃}}_{2} & & {\mathrm{\omega ̃}}_{2}^{2} & & \\ \vdots & & & \ddots & \\ {\mathrm{c̃}}_{N-1} & & & & {\mathrm{\omega ̃}}_{N-1}^{2} \end{array}\right)$$

we have

50 $$T^{T}DT=\mathrm{diag}\left(\omega_{1}^{2},...,\omega_{N}^{2}\right)$$

which gives the *N* normal-mode frequencies {ω_*j*_|*j* = 1,···,*N*} in the spin-boson model. The equilibrium position shifts $\left\{R_{j}^{eq}\right\}$ along the normal mode coordinates of the spin-boson model are given by^135,226^

51 $$\left(\begin{array}{c} R_{1}^{eq}\\ R_{2}^{eq}\\ \vdots \\ R_{N}^{eq} \end{array}\right)=2y_{0}T^{T}\left(\begin{array}{c} 1\\ -\frac{{\mathrm{c̃}}_{1}}{{\mathrm{\omega ̃}}_{1}^{2}}\\ \vdots \\ -\frac{{\mathrm{c̃}}_{N-1}}{{\mathrm{\omega ̃}}_{N-1}^{2}} \end{array}\right)$$

The reorganization energy written in terms of normal modes is then $E_{r}=\overset{N}{\underset{j=1}{\sum }}\frac{1}{2}\omega_{j}^{2}{\left(R_{j}^{eq}\right)}^{2}$ .

The MRC model extends this concept to the general *F*-state case by defining a set of *F* – 1 primary RCs within the extended-dimensional space pioneered by the MSH model. The nuclear Hamiltonian for each electronic state *X* in the MRC framework is given by a sum over the *F* – 1 subspaces, where each subspace contains one primary RC, *ŷ*_*i*_ (*i* = 1,···,*F* – 1), and a set of secondary bath modes, {*x̂*_*i,α*_|*i* = 1,···,*F* – 1;α = 1,···,*N* – 1}:^135^

$${Ĥ}_{X}=\overset{F-1}{\underset{i=1}{\sum }}\left[\frac{{\mathrm{P̂}}_{i}^{2}}{2}+\frac{1}{2}\Omega^{2}{\left({ŷ}_{i}-S^{\left(iX\right)}\right)}^{2}+\overset{N-1}{\underset{\alpha =1}{\sum }}\left(\frac{{\mathrm{p̂}}_{i,\alpha }^{2}}{2}+\frac{1}{2}{\mathrm{\omega ̃}}_{\alpha }^{2}{\left({\mathrm{x̂}}_{i,\alpha }+\frac{{\mathrm{c̃}}_{\alpha }}{{\mathrm{\omega ̃}}_{\alpha }^{2}}{ŷ}_{i}\right)}^{2}\right)\right]+\varepsilon_{X}$$ 52

This transformation is exact and can be performed systematically. While transforming from the MRC model to the MSH model involves a straightforward diagonalization of the Hessian matrix (eq 49), the reverse process of defining the primary RC from the MSH normal modes can be achieved via methods like the Householder reflection procedure.^227,228^ Importantly, the transformation ensures that the geometry of the PES minima, i.e., a polyhedron in an (*F* – 1)-dimensional space whose edge lengths are determined by the pairwise reorganization energies, is perfectly preserved in the RC space. The reorganization energies are absorbed entirely by the primary RCs, whose equilibrium positions are defined by the *F*(*F* – 1)/2 shift parameters $S_{j}^{\left(12\right)},S_{j}^{\left(13\right)},S_{j}^{\left(23\right)},...,S_{j}^{\left(F-1,F\right)}$ . The MRC model inherits all the advantages of the MSH framework, including the ability to consistently incorporate all *F*(*F* – 1)/2 reorganization energies $\sqrt{E_{r}^{\left(12\right)}},\sqrt{E_{r}^{\left(13\right)}},\sqrt{E_{r}^{\left(23\right)}},...,\sqrt{E_{r}^{\left(F-1,F\right)}}$ and describe heterogeneous, correlated environments.

We start from the first two states in the first subspace of the MSH model, which is equivalent to the asymmetric spin-boson model in eq 10 (omitting the PES minima parameters *ε*_*X*_) and the PESs of the two states are written as

53 $$\begin{array}{ll} V_{1}\left(\left\{{\mathrm{R̂}}_{j}\right\}\right) & =\overset{N}{\underset{j=1}{\sum }}\frac{1}{2}\omega_{j}^{2}{\mathrm{R̂}}_{j}^{2},\\ V_{2}\left(\left\{{\mathrm{R̂}}_{j}\right\}\right) & =\overset{N}{\underset{j=1}{\sum }}\frac{1}{2}\omega_{j}^{2}{\left({\mathrm{R̂}}_{j}-S_{j}\right)}^{2} \end{array}$$

where {*S*_*j*_} are the distances of PES minima of the two states along *j*-th normal mode of frequency ω_*j*_ (*j* = 1,···,*N*). We utilize the Householder reflection procedure to transform the normal-mode picture into the primary/secondary-mode picture. The reaction coordinate *ŷ* is defined as the specific linear combination of the MSH normal modes with a normalization factor κ:

54 $$ŷ=\frac{1}{\kappa }\overset{N}{\underset{j=1}{\sum }}\omega_{j}^{2}S_{j}{\mathrm{R̂}}_{j}$$

55 $$\kappa =\sqrt{\overset{N}{\underset{j=1}{\sum }}\omega_{j}^{4}S_{j}^{2}}$$

For the primary/secondary-mode representation, the PESs are expressed as

56 $$\begin{array}{ll} V_{1}\left(ŷ,\left\{{\mathrm{x̂}}_{\alpha }\right\}\right) & =\frac{1}{2}\Omega^{2}{ŷ}^{2}+\overset{N-1}{\underset{\alpha =1}{\sum }}\frac{1}{2}{\mathrm{\omega ̃}}_{\alpha }^{2}{\left({\mathrm{x̂}}_{\alpha }+\frac{{\mathrm{c̃}}_{\alpha }}{{\mathrm{\omega ̃}}_{\alpha }^{2}}ŷ\right)}^{2}\\ V_{2}\left(ŷ,\left\{{\mathrm{x̂}}_{\alpha }\right\}\right) & =\frac{1}{2}\Omega^{2}{\left(ŷ-\frac{\kappa }{\Omega^{2}}\right)}^{2}+\overset{N-1}{\underset{\alpha =1}{\sum }}\frac{1}{2}{\mathrm{\omega ̃}}_{\alpha }^{2}{\left({\mathrm{x̂}}_{\alpha }+\frac{{\mathrm{c̃}}_{\alpha }}{{\mathrm{\omega ̃}}_{\alpha }^{2}}ŷ\right)}^{2} \end{array}$$

The RC definition in eq 54 ensures that the reorganization energy is fully captured by the primary RC,

57 $$E_{r}=\frac{1}{2}\frac{\kappa^{2}}{\Omega^{2}}=\overset{N}{\underset{j=1}{\sum }}\frac{1}{2}\omega_{j}^{2}S_{j}^{2}$$

which in turn determines the primary mode’s characteristic frequency Ω:

58 $$\Omega =\sqrt{\frac{\kappa^{2}}{\overset{N}{\underset{j=1}{\sum }}\omega_{j}^{2}S_{j}^{2}}}$$

From eqs 56 and 57, the equilibrium position distance of the primary mode between the two states is given by

59 $$S=\frac{\kappa }{\Omega^{2}}=\frac{\sqrt{2E_{r}}}{\Omega }$$

The RC vector in eq 54 can be expressed in the normal mode basis {|*R*_1_⟩, |*R*_2_⟩,···, |*R*_*N*_⟩} as

60 $$\left|z_{1}\right\rangle =\frac{1}{\kappa }{\left(u_{1},u_{2},\cdot \cdot \cdot ,u_{N}\right)}^{T}$$

where the expansion coefficients are $u_{j}=\omega_{j}^{2}S_{j}\left(j=1,...,N\right)$ .

We define the Householder reflection operator as

61 P≡1−2|Δ⟩⟨Δ|

where $\left|\Delta \right\rangle =\frac{\left|\delta \right\rangle }{\sqrt{\left\langle \delta \right|\delta \rangle }}$ , and |δ⟩ = |*z*_1_⟩ – |*R*_1_⟩. The P reflects any vector about the plane perpendicular to the difference between the first normal mode |*R*_1_⟩ and the RC |*z*_1_⟩, and it satisfies $P\left|R_{1}\right\rangle =\left|z_{1}\right\rangle ,P\left|z_{1}\right\rangle =\left|R_{1}\right\rangle ,P=P^{T}=P^{-1}$ . A new set of orthonormal vectors $\left|z_{j}\right\rangle =P\left|R_{j}\right\rangle \left(j=1,2,...,N\right)$ can be obtained by applying the reflector to all the normal mode vectors.

The harmonic potential about its equilibrium position can be expressed in both the normal mode basis **R** = (*R*_1_,*R*_2_,···, *R*_*N*_)^*T*^ and the new basis **z** = (*z*_1_,*z*_2_,···, *z*_*N*_)^*T*^:

62 $$\begin{array}{ll} V & =\frac{1}{2}R^{T}\mathrm{diag}\left(\omega_{1}^{2},...,\omega_{N}^{2}\right)R\\ & =\frac{1}{2}z^{T}P\mathrm{diag}\left(\omega_{1}^{2},...,\omega_{N}^{2}\right)Pz=\frac{1}{2}z^{T}Kz \end{array}$$

The Hessian **K** in the new **z** basis is then

63 $$K≡P\mathrm{diag}\left(\omega_{1}^{2},...,\omega_{N}^{2}\right)P=\left(\begin{array}{ll} K_{11} & \left\langle d\right|\\ \left|d\right\rangle & K_{\mathrm{sub}} \end{array}\right)$$

where the element $K_{11}=\frac{1}{\kappa^{2}}\overset{N}{\underset{j=1}{\sum }}u_{j}^{2}\omega_{j}^{2}$ , |*d*⟩ = (*K*_21_,*K*_31_, ..., *K*_*N*1_)^*T*^ is a column vector of size *N* – 1, and sub-Hessian matrix **K**_sub_ is the bottom right (*N* – 1) × (*N* – 1) block of **K**. We then diagonalize the sub-Hessian matrix **K**_sub_ to bet the orthogonal secondary bath mode frequencies

64 $$U^{T}K_{\mathrm{sub}}U=\mathrm{diag}\left({\mathrm{\omega ̃}}_{1}^{2},{\mathrm{\omega ̃}}_{2}^{2},...,{\mathrm{\omega ̃}}_{N-1}^{2}\right)=\Lambda$$

such that the Hessian matrix in eq 49 can be achieved

65 $$\left(\begin{array}{ll} 1 & \left\langle 0\right|\\ \left|0\right\rangle & U^{T} \end{array}\right)\left(\begin{array}{ll} K_{11} & \left\langle d\right|\\ \left|d\right\rangle & K_{\mathrm{sub}} \end{array}\right)\left(\begin{array}{ll} 1 & \left\langle 0\right|\\ \left|0\right\rangle & U \end{array}\right)=\left(\begin{array}{ll} K_{11} & \left\langle \mathrm{c̃}\right|\\ \left|\mathrm{c̃}\right\rangle & \Lambda \end{array}\right)=D$$

Thus, the primary/secondary coupling coefficients are given by

66 $$\left|\mathrm{c̃}\right\rangle =\left(\begin{array}{c} {\mathrm{c̃}}_{1}\\ {\mathrm{c̃}}_{2}\\ \vdots \\ {\mathrm{c̃}}_{N-1} \end{array}\right)=U^{T}\left|d\right\rangle =U^{T}\left(\begin{array}{c} K_{21}\\ K_{31}\\ \vdots \\ K_{N1} \end{array}\right)$$

The primary mode is *ŷ* = *z*_1_, the remaining normal modes are then projected into an orthogonal basis of secondary bath modes

67 $${\left({\mathrm{x̂}}_{1},{\mathrm{x̂}}_{2},\cdot \cdot \cdot ,{\mathrm{x̂}}_{N-1}\right)}^{T}=U^{T}{\left(z_{2},z_{3},...,z_{N}\right)}^{T}$$

The resulting potential energy for the bath takes the desired primary/secondary form

68 $$\begin{array}{ll} V & =\frac{1}{2}\left(ŷ,{\mathrm{x̂}}_{1},\cdots ,{\mathrm{x̂}}_{N-1}\right)\left(\begin{array}{ll} K_{11} & \left\langle \mathrm{c̃}\right|\\ \left|\mathrm{c̃}\right\rangle & \Lambda \end{array}\right)\left(\begin{array}{c} ŷ\\ {\mathrm{x̂}}_{1}\\ \vdots \\ {\mathrm{x̂}}_{N-1} \end{array}\right)\\ & =\frac{1}{2}\Omega^{2}{ŷ}^{2}+\overset{N-1}{\underset{\alpha =1}{\sum }}\frac{1}{2}{\mathrm{\omega ̃}}_{\alpha }^{2}{\left({\mathrm{x̂}}_{\alpha }+\frac{{\mathrm{c̃}}_{\alpha }}{{\mathrm{\omega ̃}}_{\alpha }^{2}}ŷ\right)}^{2} \end{array}$$

This transformation machinery can be generalized to the *F*-state case. A key property is that the components of the transformation, including the primary RC mode *ŷ*, RC frequency Ω, and Hessian matrix **K** do not depend on the magnitude of {*S*_*j*_}, and the primary/secondary couplings {*c̃*_α_} do not depend on the reorganization energy. As a result, the reorganization energy for any pair of states is exclusively absorbed into the equilibrium positions of the primary modes. Because the MSH model already provides a valid geometric structure for the equilibrium shifts using a polyhedron whose vertices are determined by the square roots of the reorganization energies, we can directly apply this same geometry to the primary RCs in the MRC model’s (*F* – 1)-dimensional space.

The equilibrium shifts for the primary RCs, {*S* ^*(XY)*^}, are therefore determined by the same geometric construction as in the MSH model. Using the vertex coordinates {*A* ^*(1X)*^} derived from the pairwise reorganization energies and eq 59, the matrix of primary RC shifts is given by

69 $$S=\left(\begin{array}{llll} S^{\left(12\right)} & & & \\ S^{\left(13\right)} & S^{\left(23\right)} & & \\ S^{\left(14\right)} & S^{\left(24\right)} & S^{\left(34\right)} & \\ & \cdots & & \end{array}\right)=\frac{\sqrt{2}}{\Omega }\left(\begin{array}{llll} A_{1}^{\left(12\right)} & & \\ A_{1}^{\left(13\right)} & A_{2}^{\left(13\right)} & \\ A_{1}^{\left(14\right)} & A_{2}^{\left(14\right)} & A_{3}^{\left(14\right)} & \\ & \cdots & & \end{array}\right)$$

With all parameters now defined, the final Hamiltonian for the general *F*-state MRC model can be written explicitly. The nuclear Hamiltonian for each of the *F* electronic states is given by^135^

$$\{\begin{array}{ll} {Ĥ}_{1} & =\overset{F-1}{\underset{i=1}{\sum }}\frac{{{\mathrm{P̂}}_{i}}_{i}^{2}}{2}+\frac{1}{2}\Omega^{2}\left({ŷ}_{1}^{2}+{ŷ}_{2}^{2}+\cdots +{ŷ}_{F-1}^{2}\right)+{Ĥ}_{BC}+\varepsilon_{1},\\ {Ĥ}_{2} & =\overset{F-1}{\underset{i=1}{\sum }}\frac{{{\mathrm{P̂}}_{i}}_{i}^{2}}{2}+\frac{1}{2}\Omega^{2}\left[{\left({ŷ}_{1}-S^{\left(12\right)}\right)}^{2}+{ŷ}_{2}^{2}+\cdots +{ŷ}_{F-1}^{2}\right]+{Ĥ}_{BC}+\varepsilon_{2},\\ {Ĥ}_{3} & =\overset{F-1}{\underset{i=1}{\sum }}\frac{{{\mathrm{P̂}}_{i}}_{i}^{2}}{2}+\frac{1}{2}\Omega^{2}\left[{\left({ŷ}_{1}-S^{\left(13\right)}\right)}^{2}+{\left({ŷ}_{2}-S^{\left(23\right)}\right)}^{2}+\cdots +{ŷ}_{F-1}^{2}\right]+{Ĥ}_{BC}+\varepsilon_{3},\\ \cdots & \\ {Ĥ}_{F} & =\overset{F-1}{\underset{i=1}{\sum }}\frac{{{\mathrm{P̂}}_{i}}_{i}^{2}}{2}+\frac{1}{2}\Omega^{2}\left[{\left({ŷ}_{1}-S^{\left(1F\right)}\right)}^{2}+{\left({ŷ}_{2}-S^{\left(2F\right)}\right)}^{2}+\cdots +{\left({ŷ}_{F-1}-S^{\left(F-1,F\right)}\right)}^{2}\right]+{Ĥ}_{BC}+\varepsilon_{F}, \end{array}$$ 70

where the secondary bath and its coupling to the primary RCs, *Ĥ*_*BC*_, is the sum of the bath and system-bath coupling term that is common to all electronic states:

$${Ĥ}_{BC}=\overset{F-1}{\underset{i=1}{\sum }}\overset{N-1}{\underset{\alpha =1}{\sum }}\left[\frac{{\mathrm{p̂}}_{i,\alpha }^{2}}{2}+\frac{1}{2}{\mathrm{\omega ̃}}_{\alpha }^{2}{\left({\mathrm{x̂}}_{i,\alpha }+\frac{{\mathrm{c̃}}_{\alpha }}{{\mathrm{\omega ̃}}_{\alpha }^{2}}{ŷ}_{i}\right)}^{2}\right]$$ 71

The equivalence between the MSH and MRC models allows for a direct translation of nuclear coordinates between the two representations. The transformation from the MRC coordinates {*ŷ*_*i*_,{*x̂*_*i,α*_}} to the MSH normal mode coordinates {*R̂*_*a,j*_} for a given state *X* is accomplished via the transformation matrix **T** obtained from the Hessian diagonalization in eq 50:

72 $$\left(\begin{array}{c} {ŷ}_{i}-S^{\left(iX\right)}\\ {\mathrm{x̂}}_{i,1}+\frac{{\mathrm{c̃}}_{1}}{{\mathrm{\omega ̃}}_{1}^{2}}S^{\left(iX\right)}\\ \vdots \\ {\mathrm{x̂}}_{i,N-1}+\frac{{\mathrm{c̃}}_{N-1}}{{\mathrm{\omega ̃}}_{N-1}^{2}}S^{\left(iX\right)} \end{array}\right)=T\left(\begin{array}{c} {\mathrm{R̂}}_{i,1}-S_{1}^{\left(iX\right)}\\ {\mathrm{R̂}}_{i,2}-S_{2}^{\left(iX\right)}\\ \vdots \\ {\mathrm{R̂}}_{i,N}-S_{N}^{\left(iX\right)} \end{array}\right)$$

This explicit transformation confirms the formal equivalence of the two models and provides the means to switch between the geometrically intuitive normal-mode picture and the physically insightful reaction-coordinate picture.

The transformation between the primary/secondary coupled MRC model and the uncoupled normal-mode MSH model is exact and reversible. While converting from the MRC back to the MSH representation is a straightforward diagonalization of the Hessian matrix **D** (as in eq 50), the reverse transformation from MSH to MRC is more involved. An important outcome of this construction is that the secondary bath and its coupling to the primary modes, described by the Hamiltonian *Ĥ*_*BC*_, is independent of the electronic state. This property is essential for verifying that the primary modes {*ŷ*_*i*_} indeed serve as true reaction coordinates, as it isolates the state-dependent dynamics entirely within the RCs. The formal equivalence between the MSH and MRC models, established analytically, has also been confirmed through numerical simulations, for example, the PICT nonadiabatic dynamics of CPC_60_ triad conformation 3,^119,120,134^ and conformation 5 ^135^ in THF solution sampled from the triad landmark structures^229^ as well as the excitation energy transfer (EET) of FMO photosynthetic light-harvesting complexes found in *P. aestuarii* and *C. tepidum* obtained with MRC and MSH models are identical as shown in Figure 5.^119,135,173^

**Figure 5:**
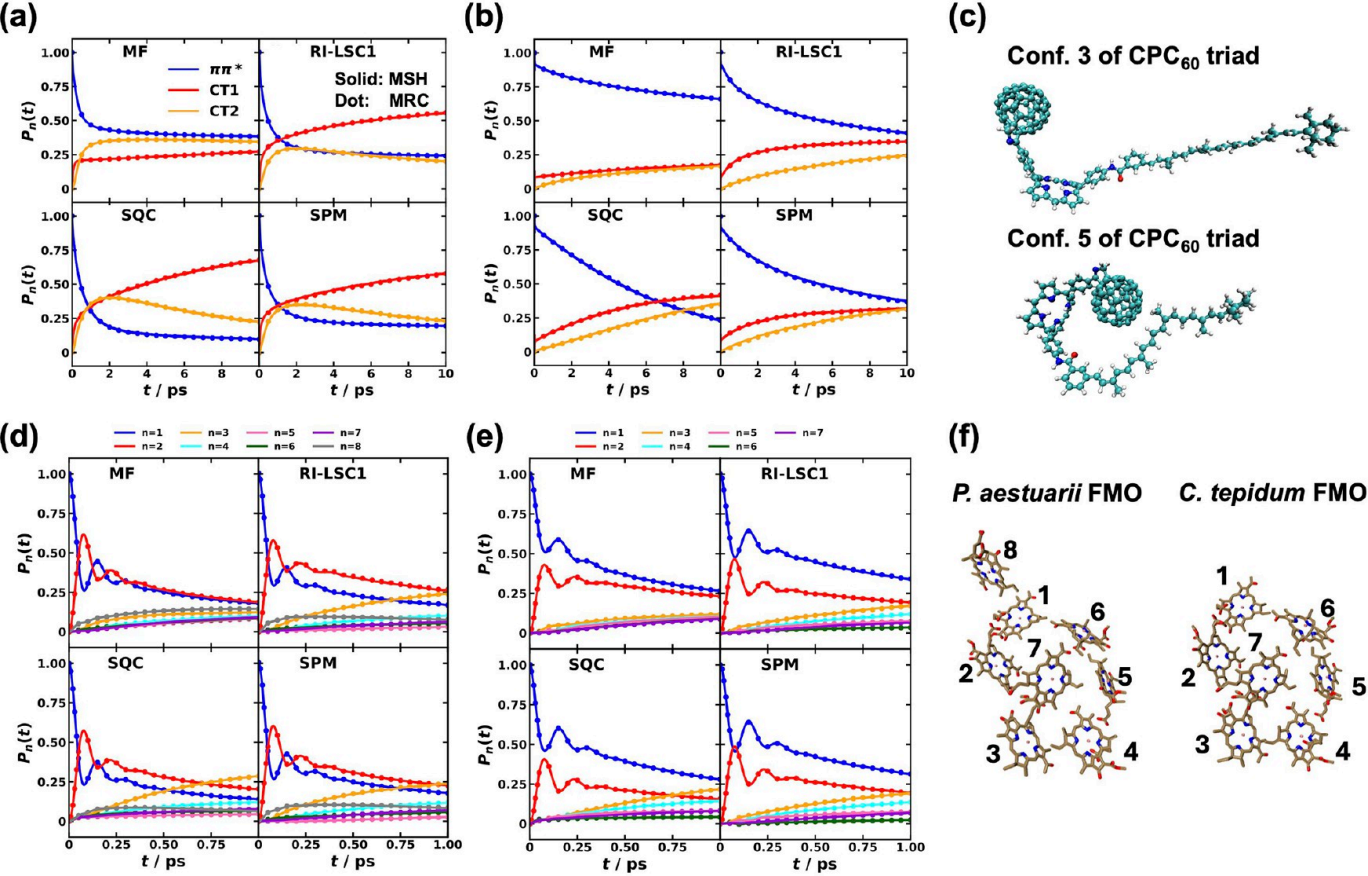
Equivalence of the MSH (solid line) and MRC (dot symbols) models for the CPC_60_ triad conformation 3 (a) and conformation 5 (b), whose structures are shown in (c) as well as the FMO light-harvesting complexes of *P. aestuarii* (d) and *C. tepidum* (e), whose chromophore arrangements are shown in (f). The nonadiabatic dynamics are obtained with MF, RI-LSC1, SQC, and SPM dynamical methods at 300 K. Reproduced from ref 135.

The PICT dynamics of the CPC_60_ triad conformation 3 shown in Figure 5a are initiated by the decay of the photoexcited ππ* state, which populates both the CT1 and CT2 states competitively. While CT1 shows a faster initial rise, both CT states reach a similar population level of about 30% within the first picosecond. Over longer time scales (up to 10 ps), the system evolves into a more complex kinetic network, where the CT2 population can decrease slightly, suggesting back-transfer or equilibration processes. In stark contrast, triad conformation 5 exhibits a dramatically different dynamical signature, as seen in Figure 5b. Its evolution is dominated by an ultrafast, coherent population oscillation between the initial ππ* state and the CT1 state within the first tens of femtoseconds (see ref 135) followed by a much slower, incoherent-like population transfer to both CT states. This pronounced dependence of the dynamics on the molecular geometry underscores the sensitivity of PICT pathways to the underlying potential energy landscape, which is defined by the reorganization energies and electronic couplings parametrized in the models. Across all of these complex, conformation-specific dynamics, the results of the MSH (solid lines) and MRC (dots) models are numerically identical. This confirms their formal equivalence and validates that the MRC representation provides an intuitive physical picture without sacrificing the accuracy of the underlying MSH construction.

This equivalence is further confirmed in the context of EET in the FMO photosynthetic complexes, as shown for *P. aestuarii* and *C. tepidum* in Figure 5d,e.^135,173^ The MSH and MRC models again yield identical population dynamics, demonstrating the robustness of the framework for describing different types of nonadiabatic processes in distinct physical systems. Beyond confirming this equivalence, the simulations provide significant biophysical insight. The models reveal distinct EET pathways for the two species; for instance, the populations of bacteriochlorophyll (BChl) sites 1 and 2 cross in *P. aestuarii* but do not in *C. tepidum*. Furthermore, the dynamics show the clear involvement of the distant BChl site 8 in the energy transfer network of *P. aestuarii* within the first 200 fs, a subtle feature captured by the model. This application also reinforces the importance of the MSH model’s sophisticated environmental description on static disorder in state-pairwise reorganization energies, realistic spectral density, and state-dependent system-bath couplings.

The MRC model inherits all the strengths of the MSH framework, such as the consistent treatment of all pairwise reorganization energies and the ability to describe heterogeneous environments, while offering several additional advantages: (1) By projecting the complex environmental dynamics onto a few key RCs, the MRC model offers an intuitive physical picture of the reaction pathway and helps to identify the collective motions responsible for driving electronic transitions. (2) The model naturally provides a system-bath partitioning that is compatible with many advanced quantum dynamics theories, like the Nakajima-Zwanzig generalized quantum master equation (GQME),^77,191,194,230–248^ which are formulated based on such a division. (3) The MRC framework allows for the straightforward preparation of arbitrary nonequilibrium initial nuclear states simply by defining a shift in the RC space.

## 5 Applications of the MSH and MRC Model Hamiltonian

Any chemical dynamical simulation can be understood through three fundamental aspects: the *Hamiltonian* that represents the physical system, the *dynamical method* used to propagate the system’s equations of motion, and the *observable* that connects the simulation to a measurable physical property such as spectroscopy.^249^ This Review is primarily concerned with the first aspect: the construction of an effective model Hamiltonian. The MSH/MRC framework serves as a powerful bridge between a high-fidelity, but computationally intractable, all-atom multistate Hamiltonian and the diverse array of dynamical methods used to simulate nonadiabatic processes. The core of this framework is the procedure for mapping the essential information from an all-atom description onto a tractable yet physically sound model. It must be emphasized that the ultimate accuracy of the MSH/MRC model is fundamentally dependent on the quality of the input all-atom Hamiltonian, which itself relies on the accuracy of the underlying electronic structure methods and force field representations. In principle, given an all-atom multistate anharmonic Hamiltonian, our approach for constructing the effective MSH/MRC Hamiltonian provides a consistent and robust mapping. This section explores the broad applications of this framework, demonstrating its utility as both a tool for simulating realistic systems and as a sophisticated theoretical platform for developing and benchmarking other methods.

### 5.1 Validation and Application to Realistic Condensed-Phase Systems

The foremost requirement of any effective model is that it must faithfully reproduce the dynamics of the full, all-atom system it aims to represent. The MSH model has been rigorously tested in this regard across several complex chemical systems where environmental heterogeneity is crucial.

#### 5.1.1 Photoinduced Charge Transfer in Liquid Solutions

The MSH model has been critically validated as a powerful tool for simulating PICT in diverse molecular architectures. Its accuracy has been rigorously tested against full all-atom simulations for both intramolecular and intermolecular CT processes. For the intramolecular case, the CPC_60_ triad serves as a key benchmark system.^119,134^ As shown in Figure 4, the nonadiabatic dynamics computed with the MSH model show excellent agreement with those from the full all-atom multistate Hamiltonian across a wide variety of semiclassical and mixed quantum-classical methods. Importantly, this study also highlighted the fundamental failure of the traditional Frenkel exciton model or IBH model to capture the correct dynamics, demonstrating the physical requirement of the correlated, globally shared bath that is built into the MSH construction.

The framework’s effectiveness extends to intermolecular PICT, as demonstrated in studies of the methylperylene (MPe) and two tetracyanoethylene (TCNE) trimer in an acetonitrile (ACN) solvent at 300 K.^250–253^ This intermolecular PICT process starts with the excitonic (EX) state located on the MPe molecule and later could form charge-transfer states between the MPe and the first or the second TCNE molecules, labeled as CT1 and CT2, respectively. As shown in Figure 6, the MSH model’s dynamics again excellently reproduce the all-atom reference results for various nonadiabatic dynamics methods, especially the more accurate RI-LSC2/3 and CMM methods. The SD-IBH gives less accurate predictions, and the SI-IBH generates the worst result compared with MSH. From the PES triangle shape in Figure 6a, we observe that the angle between CT1-EX-CT2 minima is less than 90°, indicating correlated bath and explaining the deviation seen in SD-IBH that assumes orthogonal relation. Together with the triad case, these applications to both intramolecular (between covalently linked moieties) and intermolecular CT systems establish the broad robustness and physical necessity of the MSH model for accurately simulating photoinduced charge transfer in the condensed phase.

**Figure 6:**
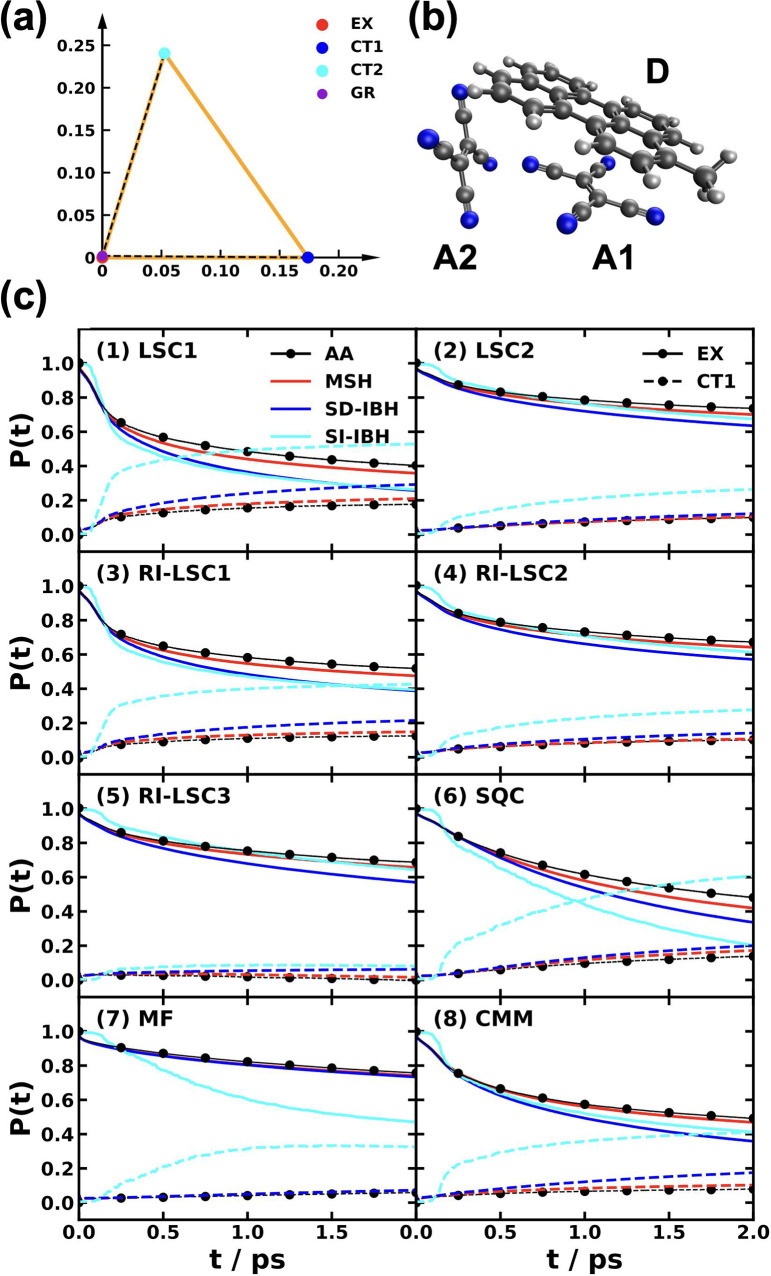
Photo-induced intermolecular charge transfer dynamics of MPe and two TCNE trimer. Panel (a) shows the PES minima of the excitonic state (EX) located on MPe, the charge transfer state between MPe and TCNE #1 (CT1) and between MPe and TCNE #2 (CT2), and the ground state (GR). Panel (b) shows the geometry of the trimer. Panel (c) shows the EX (solid) and CT1 (dash) population dynamics of MPe/2TCNE trimer dissolved in acetonitrile solvent at 300 K obtained with all-atom Hamiltonian (black with dot symbols), MSH (red), SD-IBH (blue), and SI-IBH (cyan) models using different semiclassical dynamical methods.

#### 5.1.2 Excitation Energy Transfer in Multichromophoric Systems

The MSH model has been applied to provide a more physically grounded description of EET in the FMO photosynthetic light-harvesting complex, a prototypical system for studying biological energy conversion.^173^ Theoretical studies of this protein have traditionally relied on the Frenkel exciton model, a type of IBH model.^103,112,121,122,141–149,151^ The central limitation of the IBH model for a realistic system like FMO is the assumption that each BChl pigment couples to an identical and isolated bath, often described by an empirical Debye spectral density. This neglects the specific, heterogeneous protein environment surrounding each pigment and the potential for correlated fluctuations between them.

The MSH framework provides a more rigorous approach by using parameters derived from all-atom Quantum Mechanics/Molecular Mechanics (QM/MM) simulations^167^ to construct a globally shared bath that couples to each pigment site according to its unique, realistic environment. Comparing the dynamics predicted by the two distinct models reveals a noticeable difference in the EET population dynamics. As shown in Figure 7, the MSH model with a realistic spectral density and site-dependent system-bath couplings displays slower EET dynamics than the conventional Frenkel exciton (SI-IBH) model.^173^ Although the difference is not drastic, it highlights the tangible impact of using a more physically rigorous environmental description. The systematic comparison of various MSH and IBH model variants allowed for a deeper analysis of the factors controlling the energy transfer rates. This work showed that a larger average reorganization energy, heterogeneity in the spectral densities, and a greater weight of low-frequency modes could facilitate energy dissipation and thus accelerate EET. Interestingly, the dynamics were found to be relatively insensitive to the static disorder in the site-to-site reorganization energies in the EET of FMO complex. This application of the MSH model demonstrates its power to dissect which specific features of the system-bath interaction are most important for biological function.

**Figure 7:**
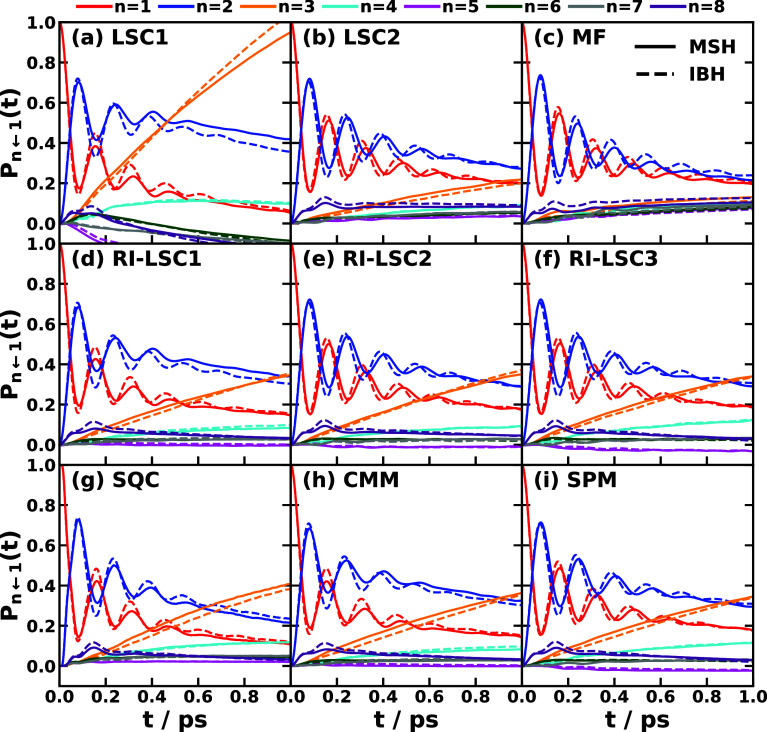
Comparison of MSH model (solid) and the traditional IBH model (dash) for excitation energy transfer in 8-site *P. aestuarii* FMO complex after initial excitation on BChl 1 at 77 K as obtained with LSC1, LSC2, MF, RI-LSC1–3, SQC with square window, CMM (γ = 1/2), and SPM methods, reproduced from ref 173.

The MSH framework is also being applied to understand EET in newly discovered organic photovoltaic materials, such as dimers of the nonfullerene acceptor (NFA) Y6,^254–260^ which are central candidates for the next generation of modern organic solar cells. In these multichromophoric systems, the interplay between efficient EET (exciton diffusion) and the coupling to CT states is an essential factor for device performance. The MSH model is uniquely suited for this challenge, as it can rigorously describe the shared environmental bath and the electronic-vibrational couplings between the different electronic states of the dimer.

To validate this approach for artificial materials, a model system was constructed consisting of two Y6 molecules in a face-to-face alignment dissolved in a chloroform solvent with periodic boundary conditions. A key characteristic of this system, revealed by analyzing the pairwise spectral densities between the two locally excited states (EX1, EX2) and the ground state (Figure 3), is that the overall structured shapes of the spectral densities are very similar for different transitions and are independent of the sampling state. The MSH model was then used to simulate the EET process, which involves population transfer from an initially photoexcited molecule (EX1) to its neighbor (EX2). As shown in Figure 8, the nonadiabatic dynamics of the Y6 dimer simulated using the MSH model accurately reproduce the all-atom nonadiabatic dynamics at room temperature for Y6 molecules separated by 5 Å, 10 Å, and 20 Å in Figure 8c–e, gradually transition from correlated bath to uncorrelated bath as shown in Figure 8a. The SD-IBH is seen to have a systematic underestimation of population transfer compared with the all-atom results, especially when the intermolecular distance gets larger, while the SI-IBH shows a smaller oscillation amplitude than the all-atom reference in all cases. This successful application to the Y6 dimer in organic solvent serves as another critical check of the MSH model’s effectiveness, confirming its ability to reproduce realistic nonadiabatic EET dynamics in artificial multichromophoric systems in the condensed phase.

**Figure 8:**
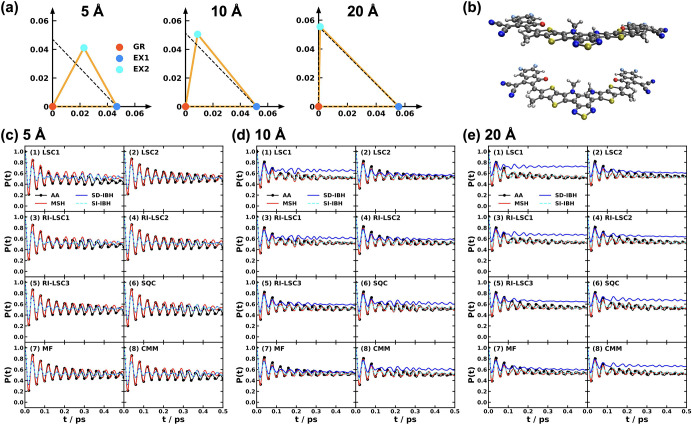
Comparison of excitation energy transfer dynamics of Y6 dimer separated by different distances in chloroform solvent at 300 K. Panel (a) shows the PES minima triangles of ground state (GR) and excitonic states (EX1 and EX2) for the Y6 dimer at separations of 5 Å, 10 Å, and 20 Å. Panel (b) shows the geometry of Y6 dimer separated by 10 Å. Panels (c), (d), and (e) show the excitonic state (EX1) population dynamics of all-atom multistate Hamiltonian (black with dot symbols), MSH (red solid), SD-IBH (blue solid), and SI-IBH (cyan dash) models for Y6 dimer at separations of 5 Å, 10 Å, and 20 Å, respectively.

### 5.2 MRC Representation: Physical Insight and Practical Utility

While the MSH model is formally complete, its representation in terms of delocalized normal modes can obscure a clear physical picture of the nonadiabatic process. The MRC model, which is an exactly equivalent representation, was developed to restore this intuition. Figure 5 explicitly demonstrates this equivalence, showing that the nonadiabatic dynamics of both the CPC_60_ triad confirmations 3 and 5 and the FMO complexes from *P. aestuarii* and *C. tepidum* are identical whether computed with the MSH or MRC Hamiltonians.^135^ If starting with the same initial sampling, the numerical results of the electronic populations obtained with MSH and MRC Hamiltonians are identical within 11 significant figures in the first tens of steps and within 7 significant figures at 1 ps, proving the numerical equivalence, and the small difference is due to the computer’s double precision rather than the transformation. RI-LSC1, SQC, and SPM dynamics tend to be similar and are believed to be more accurate than MF dynamics. The nonadiabatic dynamics predicted by the MSH model have been demonstrated to agree with those obtained with the explicitly all-atom multistate Hamiltonian of the triad in Figure 4. The agreement among MSH, MRC, and all-atom Hamiltonians in combination with the same dynamical method suggests the effectiveness of the simplified MSH and MRC models in studying the nonadiabatic dynamics in complex condensed-phase systems.

The primary advantage of the MRC representation is the direct access it provides to the reaction coordinate trajectories, a physically insightful observable that distills the high-dimensional environmental dynamics into an (*F* – 1)-dimensional, visualizable pathway. Figure 9 shows these RC trajectories for both the triad and FMO systems. While individual trajectories exhibit wild oscillatory characteristics of thermal fluctuations, the ensemble-averaged RC trajectory reveals a smooth, deterministic path that maps the collective nuclear motion driving the electronic transition, offering a direct and visual understanding of the reaction mechanism.

**Figure 9:**
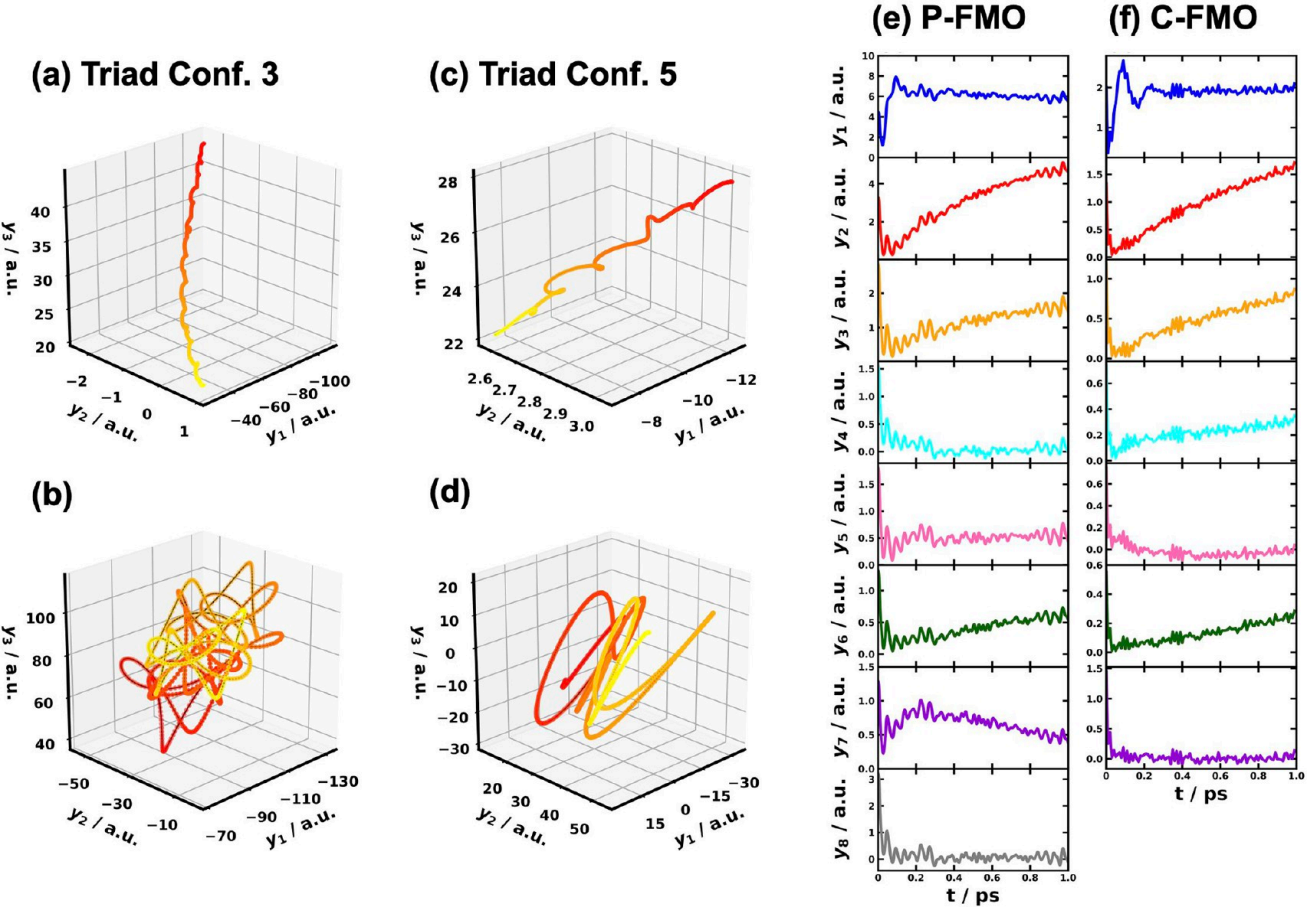
Reaction coordinate (RC) trajectories for various systems simulated with the MRC model using SQC dynamics. Panels (a, b) and (c, d) show the RC trajectories for conformations 3 and 5 of the CPC_60_ triad, respectively. Panels (a, c) depict the ensemble average over 10^5^ trajectories, while (b, d) show single RC trajectories. Panels (e) and (f) show the ensemble-averaged RC trajectories for the FMO complexes of *P. aestuarii* and *C. tepidum*, respectively, averaged over 10^6^ trajectories. Reproduced from ref 135.

The analysis of these trajectories provides a powerful tool for comparing and understanding the dynamics of different systems. For instance, the RC trajectories for the 8-site *P. aestuarii* and 7-site *C. tepidum* FMO complexes reveal distinct dynamical signatures that reflect their underlying structural and energetic differences. The RC component *y*_1_, which connects the PES minima of sites |1⟩ and |2⟩, displays larger oscillations in the *C. tepidum* FMO, consistent with the more pronounced population oscillations observed for this species. Furthermore, the RC trajectories clearly exhibit two distinct time scales: an ultrafast initial response on a ∼500 fs time scale where all RC components are activated, followed by a slower, multipicosecond drift in several components that governs the longer-time equilibration of the EET process. The amplitudes of these RC motions are also informative; the smaller RC amplitudes in the *C. tepidum* complex can be traced back to its higher characteristic reaction coordinate frequency (1187.2 cm^–1^) compared to that of *P. aestuarii* (507.68 cm^–1^). Thus, the RC trajectories provide a rich, multidimensional signature of the nonadiabatic process, directly linking the collective nuclear motions to the observed electronic dynamics and the underlying parameters of the model Hamiltonian.

Furthermore, the MRC framework has significant practical utility for exploring how to control reaction dynamics. As shown in Figure 10, the low-dimensional RC space allows for the straightforward preparation of arbitrary nonequilibrium initial nuclear states, providing a powerful tool to systematically investigate how the initial nuclear configuration influences the subsequent nonadiabatic dynamics.^135^ This capability was demonstrated for the triad system, where the population transfer dynamics were shown to be highly sensitive to the initial nuclear conditions. While keeping the initial electronic state on the ππ* level, initiating the dynamics from the ground state’s equilibrium nuclear density, mimicking a vertical photoexcitation, resulted in faster population transfer than starting from nuclear configurations equilibrated on any of the excited states. To further probe this relationship, dynamics were initiated from two arbitrary points in the RC space (V1 and V2). The simulations revealed a clear trend: starting from V2 led to significantly faster population transfer, while starting from V1 resulted in slower dynamics. This systematic exploration using MRC model demonstrates a direct link between the initial location in the collective nuclear coordinate space and the efficiency of the subsequent electronic transitions, offering a conceptual basis for the rational control of photochemical processes.

**Figure 10:**
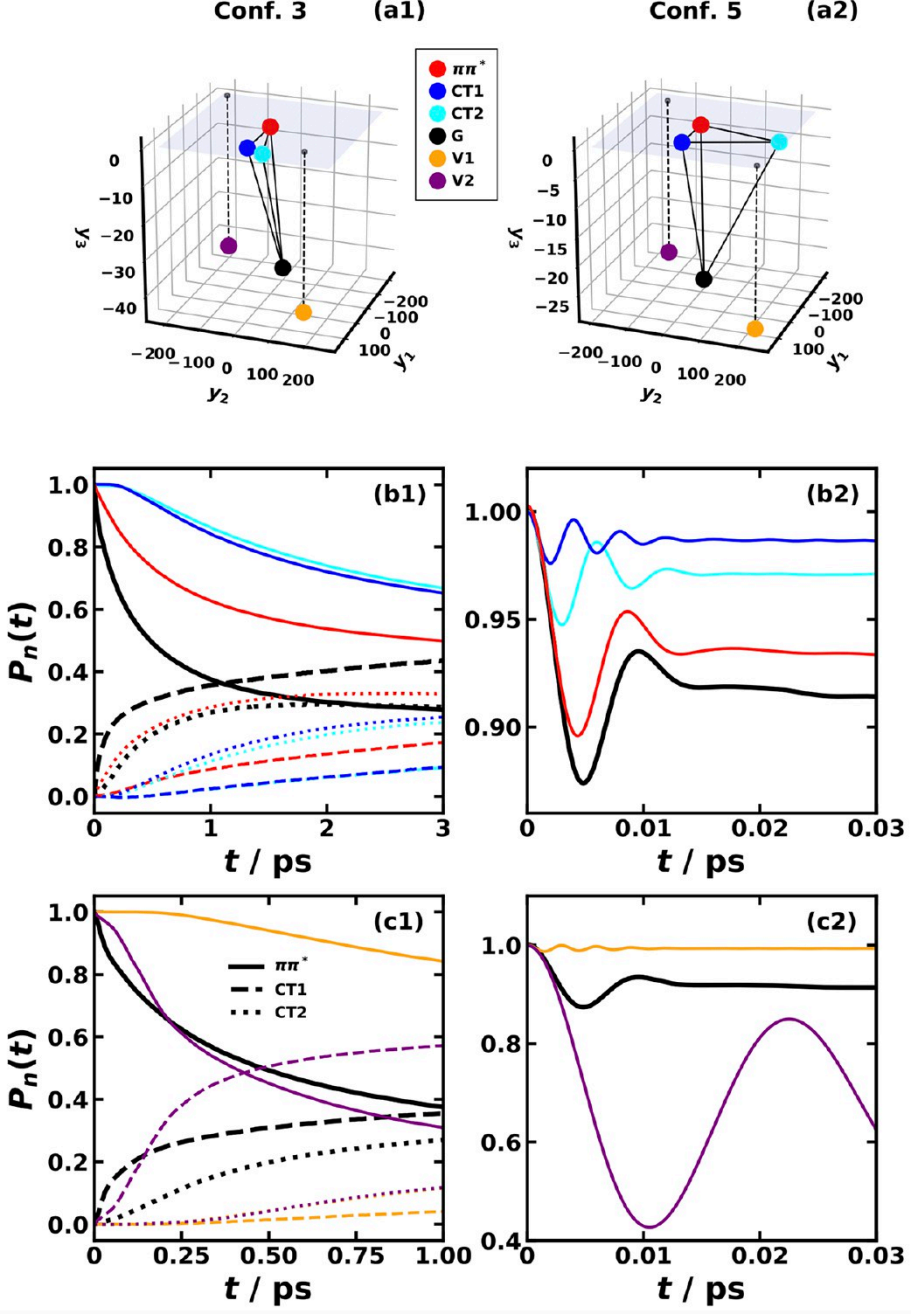
Effect of the initial nuclear state on the nonadiabatic dynamics of the CPC_60_ triad, simulated with the MRC model and SQC method at 300 K. Panels (a1) and (a2) illustrate the PES minima for the ground (G), ππ*, CT1, and CT2 states, along with two arbitrary starting points (V1, V2), in the 3D reaction coordinate space for conformations 3 and 5, respectively. The subsequent panels show the resulting population dynamics, initiated from the |ππ*⟩ ⟨ππ*| electronic population, for various initial nuclear distributions: (b1, b2) starting from ππ* (red), CT1 (blue), and CT2 (cyan) states, and (c1, c2) starting from the arbitrary points V1 (orange) and V2 (purple), compared with starting from the ground state (black). Reproduced from ref 135.

### 5.3 A Platform for Method Development and Exploration

The MSH/MRC framework, being both physically realistic and often analytically tractable, serves as an ideal theoretical platform for developing, testing, and understanding other theoretical methods, from foundational rate theories to advanced quantum dynamics algorithms.

#### 5.3.1 Exploring the Parameter Space of Nonadiabatic Dynamics

The inherent tunability of the MSH model makes it a powerful tool for exploring the fundamental physics of nonadiabatic transitions. A systematic scan of the MSH parameter space was performed to map out the characteristic dynamical behaviors in different physical regimes, as shown in Figure 11. Following the conventional classification, the parameter space was divided into the nonadiabatic (Γ/ω_*c*_ < 1) to adiabatic (Γ/ω_*c*_ > 1) regimes by varying the ratio of the electronic coupling (Γ) to the characteristic bath frequency (ω_*c*_), and into the Marcus normal (Δ*E*/*E*_*r*_ < 1) to inverted (Δ*E*/*E*_*r*_ > 1) regimes^261–264^ by varying the ratio of the reaction free energy (Δ*E*) to the reorganization energy (*E*_*r*_). A series of 45 three-state MSH model variations (S1–S9 with five variations each) were simulated at 300 K using the SQC with triangle window^203^ to systematically explore the parameter space. Each series was built around a central base model (c), with variants representing systematically altered conditions: low (a) and high (e) electronic coupling, alongside large (b) and small (d) reorganization energies.

**Figure 11:**
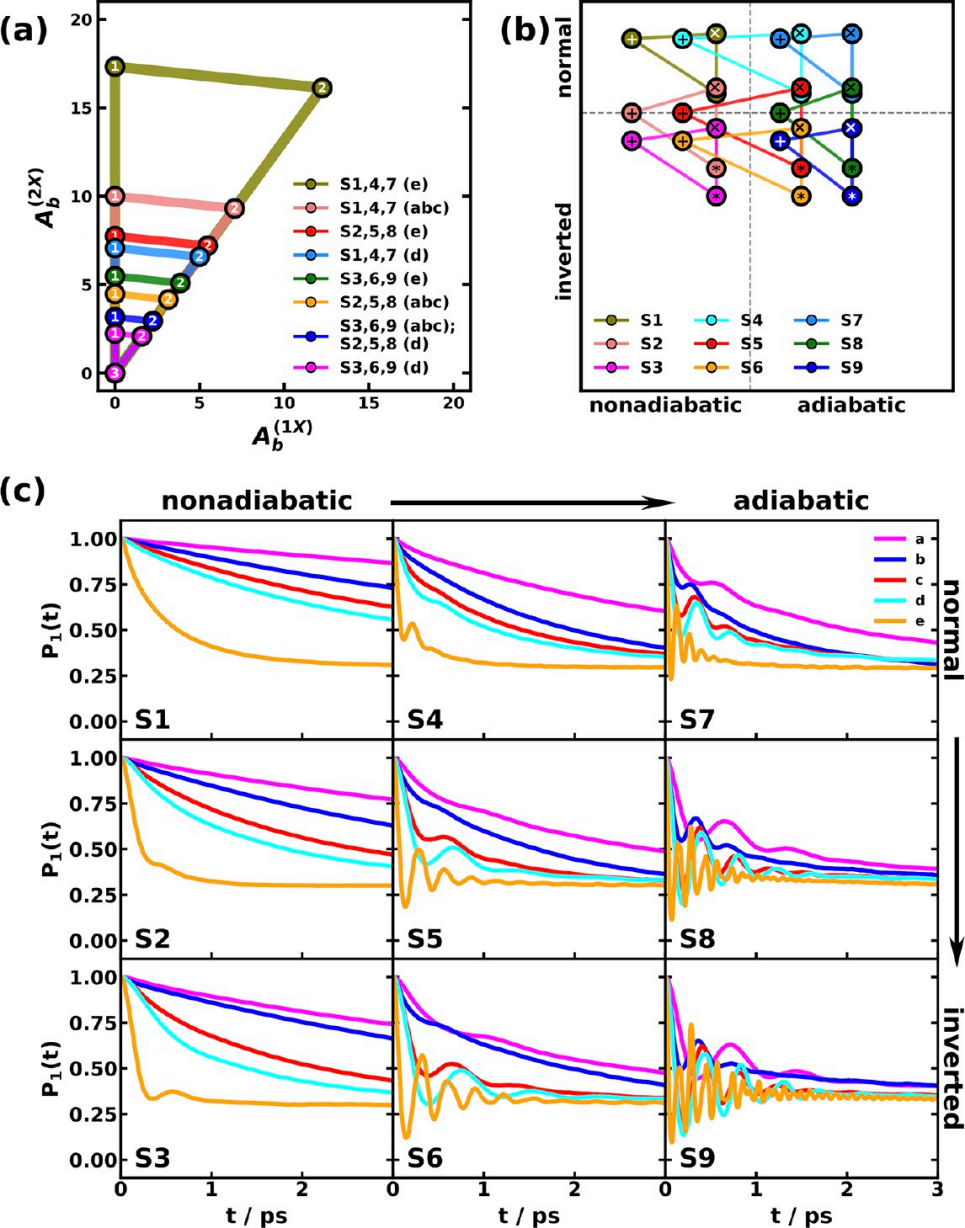
Exploration of the MSH parameter space. Panel a) shows PES minima relations of MSH models S1–S9, with the labels on circles denoting the electronic state. Panel (b) shows the parameter regimes for MSH models S1–S9, where the circles labeled with ∗, + and × correspond to transitions between |1⟩ ↔ |2⟩, |1⟩ ↔ |3⟩ and |2⟩ ↔ |3⟩ transitions; the regimes of Δ*E*/*E*_*r*_ < 1 (normal) and Δ*E*/*E*_*r*_ > 1 (inverted) across vertical axis and regimes of Γ/ω_*c*_ < 1 (nonadiabatic) and Γ/ω_*c*_ > 1 (adiabatic) across horizontal axis. Panel (c) shows population dynamics of state |1⟩ in MSH models S1–S9 with different variants at 300 K obtained using SQC with the triangle window method. Reproduced from ref 265 with permission of AIP Publishing.

These explorations provide a comprehensive map of how key physical parameters shape nonadiabatic dynamics between multiple states, with the results summarized in Figure 11. The geometric arrangement of the PES minima for the S1–S9 models forms a series of scaled triangles, as shown in Figure 11a, with the corresponding parameter regimes for the three pairs of states plotted in Figure 11b. The simulations revealed clear and physically intuitive trends. Moving from the nonadiabatic to the adiabatic regime (left to right in Figure 11b), by increasing the electronic coupling, leads to more rapid population decay and stronger coherent oscillations, reflecting the enhanced diabatic coupling. Similarly, moving from the normal to the inverted regime (top to bottom in Figure 11b) by lowering reorganization energy also accelerates population transfer and amplifies oscillations as shown in Figure 11c. The most rapid population transfer and the strongest oscillations are observed in the adiabatic-inverted limit, which corresponds to the corner of the parameter space with large coupling and low reorganization. Within any single panel in Figure 11c, increasing the electronic coupling or decreasing the reorganization energy was found to accelerate the dynamics and amplify the oscillatory pattern in population. These systematic explorations provide a comprehensive map of the dynamical behavior as a function of the key physical parameters that govern nonadiabatic processes.

The MSH model also enables the targeted study of more subtle environmental effects, such as the degree of bath correlation. Here, we explore this effect by performing CMM dynamics for a series of three-state MSH models at 300 K, where the angle θ_23_ defining the triangular geometry of the PES minima was systematically scanned from 45° to 135°. As depicted in Figure 12a, this geometric tuning continuously modulates the bath from being correlated (e.g., θ_23_ = 45°) to uncorrelated (θ_23_ = 90°), and to anticorrelated (e.g., θ_23_ = 135°). In these simulations, the initial electronic state is the excited state |2⟩, whose PES minimum is located on the horizontal axis *R*_1_, while the initial nuclear sampling is performed on the ground state |1⟩, whose PES minimum is located at the origin. The spectral density is chosen as Ohmic form with characteristic frequency ω_*c*_ = 10 cm^–1^. The reorganization energies between the ground state and excited states were set to $E_{r}^{\left(12\right)}=E_{r}^{\left(13\right)}=100\;{\mathrm{cm}}^{-1}$ , with the correlation-dependent term being $E_{r}^{\left(23\right)}=\left(1-\cos\theta_{23}\right)200\;{\mathrm{cm}}^{-1}$ . The energy minima were *ε*_1_ = 0 and *ε*_2_ = *ε*_3_ = 100 cm^–1^. Two distinct electronic coupling scenarios were investigated to probe different dynamical regimes: a strong-coupling case shown in Figure 12b, where the interaction between the excited states |2⟩ and |3⟩ is dominant (Γ_12_ = Γ_13_ = 10 cm^–1^ and Γ_23_ = 100 cm^–1^), and a weak-coupling case shown in Figure 12c where it is suppressed (Γ_12_ = Γ_13_ = 100 cm^–1^ and Γ_23_ = 10 cm^–1^).

**Figure 12:**
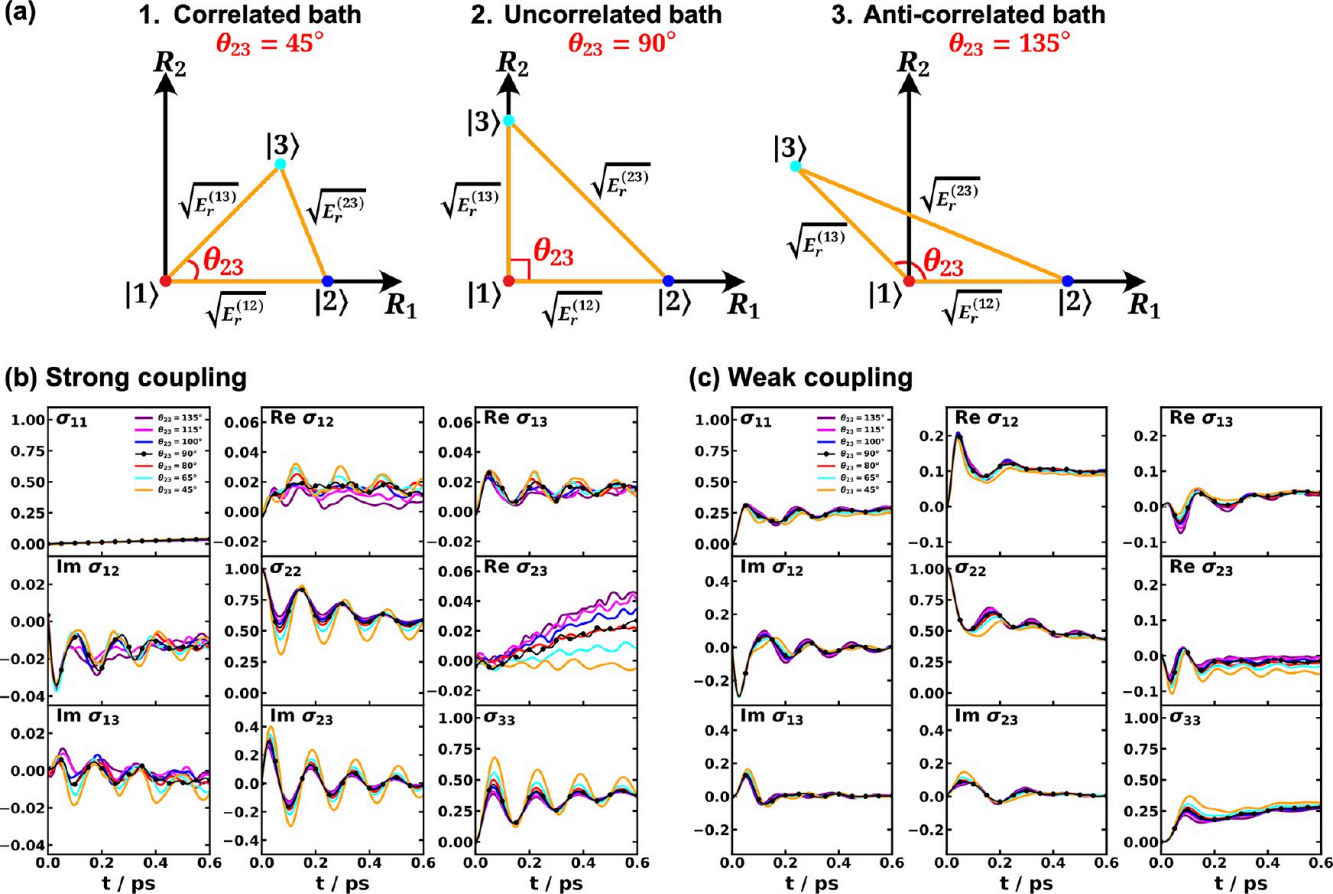
Effect of bath correlation in three-state MSH models with different angle θ_23_. Panel (a) shows PES relations for correlated, uncorrelated, and anticorrelated baths with θ_23_ = 45°, 90°, and 135°, respectively. Panels (b) and (c) show the RDM dynamics for the MSH models of different θ_23_ with strong coupling Γ_23_ = 100 cm^–1^ and weak coupling Γ_23_ = 10 cm^–1^, respectively, obtained with CMM method at 300 K.

This analysis revealed that the effect of bath correlation on the dynamics is drastically different in the weak and strong electronic coupling regimes, highlighting the model’s utility for dissecting nuanced environmental roles. A key insight is that the uncorrelated case, where θ_23_ = 90°, is exactly equivalent to the IBH or Frenkel exciton model. In the strong-coupling case shown in Figure 12b, the correlated-bath MSH models exhibit a larger oscillation amplitude in the populations of states |2⟩ and |3⟩ than the IBH case; conversely, the anticorrelated bath models tend to suppress the oscillation amplitude. This phenomenon arises because the electronic coupling between |2⟩ and |3⟩ is large, and population exchange becomes more efficient when the two electronic states share a correlated bath that facilitates concerted nuclear motions. In the weak-coupling case, shown in Figure 12c, we observed that the correlated bath leads to a faster overall population transfer from the initially populated state |2⟩ to both |1⟩ and |3⟩. The population transfer likely proceeds through a stepwise pathway, |2⟩ → |1⟩ → |3⟩, which can be traced back to the weak direct coupling between states |2⟩ and |3⟩. This systematic exploration demonstrates the MSH framework’s power in revealing how specific environmental properties, like bath correlation, can fundamentally alter nonadiabatic dynamics depending on the intrinsic electronic-vibrational coupling of the system.

#### 5.3.2 Benchmarking Approximate Dynamics Against Numerically Exact Dynamics

A significant application of the MSH framework is its role as a robust platform for method development and validation. While the MSH model provides a physically grounded representation of a complex system, the choice of dynamical method used to propagate its equations of motion remains a critical factor. The challenge in any quantum dynamics simulation is the “curse of dimensionality”, where the coefficients needed to represent a wave function $\Psi \in C^{n_{1}\times \cdot \cdot \cdot \times n_{d}}$ is a *d*-dimensional tensor, and the computational cost of the wavepacket

73 $$\left|\Psi \left(t\right)\right\rangle =\overset{n_{1},...,n_{d}}{\underset{j_{1},...,j_{d}}{\sum }}\Psi \left(j_{1},...,j_{d};t\right)\left|j_{1},...,j_{d}\right\rangle$$

scales exponentially with the number of DOF, *d*. The tensor-train (TT) formalism,^266^ also known as the matrix product state (MPS) representation,^267^ circumvents this issue by decomposing the high-dimensional tensor of coefficients into a contracted product of smaller, third-order core tensors:

$$\Psi \left(j_{1},...,j_{d}\right)=\Psi_{1}\left(j_{1}\right)\Psi_{2}\left(j_{2}\right)\cdot \cdot \cdot \Psi_{d}\left(j_{d}\right)$$ 74

This structure reduces the storage complexity from an exponential scaling to a much more manageable linear scaling, *O*(*dnr̃* ^2^), where *n* is the local basis size and *r̃* is the TT-rank. For time propagation, the tensor-train KSL (TT-KSL) algorithm serves as an efficient dynamical low-rank approximation, integrating the time-dependent Schrödinger equation, $\frac{d}{dt}\left|\Psi \left(t\right)\right\rangle =-\frac{i}{\hbar }Ĥ\left|\Psi \left(t\right)\right\rangle$ , while maintaining the TT format of the wave function.^268–270^

To address finite-temperature dynamics, which are governed by the quantum Liouville equation for the density matrix, $\frac{d}{dt}\mathrm{\rho ̂}\left(\beta ,t\right)=-\frac{i}{\hbar }\left[Ĥ,\mathrm{\rho ̂}\left(\beta ,t\right)\right]$ , the TT framework can be combined with thermofield dynamics (TFD).^247,271–273^ The core idea of TFD is to represent the mixed-state density operator as a pure-state thermal state vector, |ψ­(β, *t*)⟩, in a double Hilbert space composed of the original physical space and an identical, fictitious “tilde” space. The evolution of this thermal state vector is governed by finite-temperature Schrödinger equation,

75 $$\frac{d}{dt}\left|\psi \left(\beta ,t\right)\right\rangle =-\frac{i}{\hbar }\mathrm{H̅}\left|\psi \left(\beta ,t\right)\right\rangle$$

Here, the double-space Hamiltonian is defined as *H̅* = *Ĥ* ⊗ *Ĩ* − *Î* ⊗ *H̃*_*f*_, where Kronecker product *Â* ⊗ *B̃* is between a physical-space operator *Â* and a fictitious-space operator *B̃*, and *H̃*_*f*_ can be chosen arbitrarily. The thermal average of any physical observable *Â* is simply given by the expectation value ⟨*A*(*t*)⟩ = ⟨ψ­(β,*t*)|*Â*|ψ­(β,*t*)⟩. The harmonic nature of the MSH model is particularly synergistic with this TT-TFD framework. Specifically, the preparation of the initial thermal equilibrium state is given by

76 $$\left|\psi \left(\beta ,0\right)\right\rangle =\frac{1}{Z{\left(\beta \right)}^{1/2}}e^{-\beta Ĥ/2}\underset{n}{\sum }\left|n\right\rangle ⊗\left|ñ\right\rangle$$

where *Z*(β) is the partition function at inverse temperature β. This is computationally demanding for general systems, can be performed analytically for harmonic models by utilizing the Bogoliubov transformation.^271,274^ This transformation maps the complex thermal state onto a simple, rank-1 vacuum state in the double Hilbert space, providing a highly efficient starting point for the subsequent time evolution with the TT-KSL algorithm. This capability allows us to generate benchmark quantum dynamics for these complex, multistate model systems, providing a rigorous reference against which the accuracy of various widely used and computationally efficient approximate methods can be systematically evaluated. These methods include the LSC approaches and their RI variants,^119,120,150,185–198^ SQC,^139,199–206^ CMM,^211–216^ MF Ehrenfest dynamics,^209,210^ and fewest-switches surface hopping (FSSH).^275,276^

An example of such a benchmark is presented in Figure 13, which shows the population dynamics of the MSH model S5­(d) at temperatures of 0, 77, and 300 K. This model was specifically chosen because it resides in an intermediate regime, positioned between the nonadiabatic and adiabatic limits as well as between the normal and inverted Marcus regimes, presenting a challenging test case for approximate methods. At zero temperature, the numerically exact TT-KSL dynamics reveal that while most approximate methods capture the initial ultrafast decay and oscillations, they begin to diverge from the exact result after approximately 1 ps. Among the approximate approaches, the SQC, RI-LSC2, and CMM methods demonstrate the most accurate performance over longer time scales. As the temperature increases to 77 and 300 K, the quantum coherences are increasingly damped, and the system dynamics become more classical. Consequently, the discrepancies between the various approximate methods and the exact TT-TFD results diminish substantially, with most methods converging to the correct dynamics at room temperature. This systematic comparison not only validates the use of certain semiclassical methods in specific regimes but also provides a clear map of their domains of validity, offering practical guidance for selecting the appropriate level of theory for a given physical problem.

**Figure 13:**
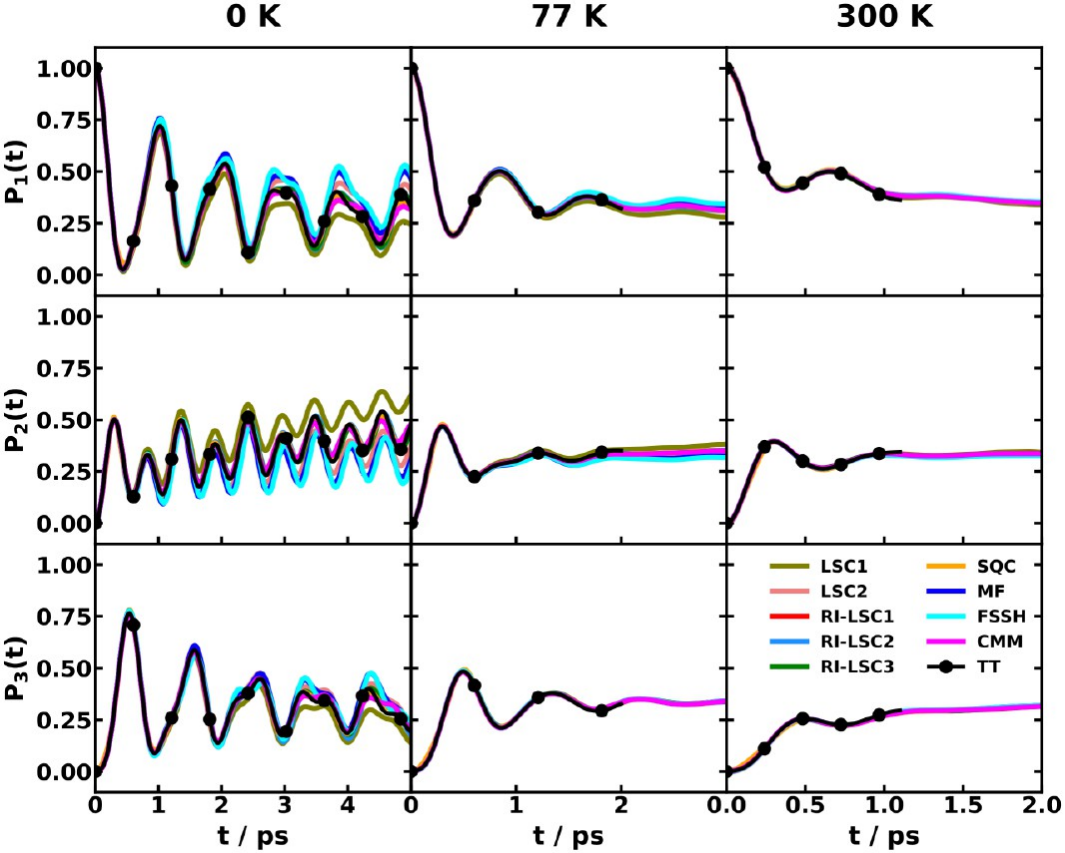
Comparison of numerically exact and semiclassical dynamics of MSH S5­(d) model at different temperatures, where exact result is simulated with tensor-train dynamics. Reproduced from ref 265 with permission of AIP Publishing.

The combination of the MSH model with numerically exact methods also provides deeper insights into the nature of the system-bath coupling itself, particularly the role of the bath’s dimensionality. Figure 14 illustrates the effect of varying the number of physical normal modes, *N*, from 20 to 60 for the MSH model of a Y6 dimer at distance of 10 Å in chloroform solution at 300 K, whose spectral densities were obtained from all-atom simulations of the Y6 dimer in chloroform solvent are in Figure 3. The EET process involves population transfer between two locally excited states on the two Y6 molecules, denoted as |1⟩ and |2⟩, starting with initially populated state |1⟩. The nonadiabatic dynamics were simulated using TT-TFD and a suite of approximate methods. A critical observation emerges from this study: when the number of bath modes is small (*N* = 20), the numerically exact TT-TFD dynamics exhibit larger quantum oscillations in both population and coherence than the tested semiclassical dynamics. However, as the number of modes increases to 40 and then 60, thereby creating a denser manifold of vibrational states, these oscillations in the exact dynamics are damped, reflecting more realistic quantum decoherence due to the system-bath interaction. The TT-TFD results begin to converge toward the predictions of the semiclassical methods, which themselves are largely insensitive to the number of bath modes.

**Figure 14:**
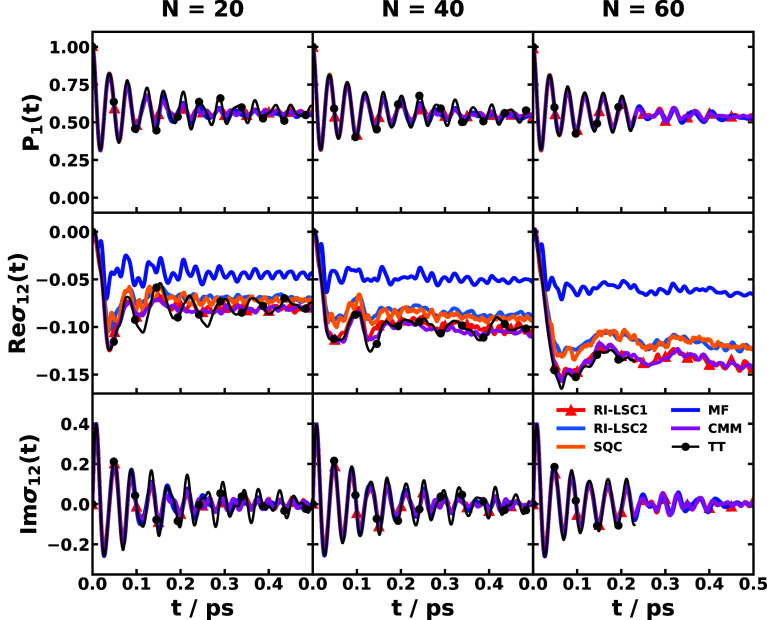
Effect of number of modes (*N* = 20, 40, 60) in MSH model of the Y6 dimer at distance of 10 Å dissolved in chloroform at 300 K, simulated with various semiclassical dynamics and numerically exact TT-TFD methods.

This convergence carries a profound implication for method benchmarking. It demonstrates that comparing an approximate method to an exact simulation of a model with an insufficient number of bath modes can be misleading. A sparse bath may exhibit artificial quantum recurrences that are not representative of a true condensed-phase environment, leading one to incorrectly conclude that an approximate method is inaccurate. The fact that the exact dynamics approach the semiclassical results as the bath becomes denser suggests that these approximate methods are particularly suited for condensed-phase systems. In such environments, the collective dephasing effect of a large number of bath modes is a dominant physical feature, and this is precisely what the semiclassical approaches are effective at capturing. This finding not only validates the use of methods like LSC, CMM, and SQC for complex systems but also reinforces the power of the MSH model as a flexible and physically realistic platform for understanding the fundamental interplay between quantum system and the environment.

#### 5.3.3 Applications to Rate Constant and Time-Dependent Rate Theories

Beyond its use in direct nonadiabatic dynamical simulations, the MSH framework serves as an ideal theoretical model for applying and testing rate theories in complex, multistate systems. The model’s parameters, which include all pairwise reorganization energies $\left(E_{r}^{\left(XY\right)}\right)$ , electronic couplings (Γ_*XY*_) and reaction free energies (Δ*E* ^(*XY*)^), provide the necessary inputs for established theories like Marcus theory^85,277–282^ and equilibrium Fermi’s golden rule (FGR) for charge transfer rate constant^77,78,105,137,283–292^ and the more advanced nonequilibrium Fermi’s golden rule (NE-FGR) for time-dependent rate.^113,134,226,293–301^ This capability allows for a systematic investigation of how the fundamental parameters of a system govern the kinetics of all competing and sequential transfer pathways.

The MSH model is particularly well-suited for exploring the Marcus landscape of a multistate system. Because it is parametrized with all pairwise $E_{r}^{\left(XY\right)}$ , Γ_*XY*_, and Δ*E* ^(*XY*)^ values, a Marcus rate constant can be calculated for every possible transition, for example, *X* → *Y* transition has,^85,277,278^

77 $$k_{X\to Y}^{\mathrm{Marcus}}=\frac{\Gamma_{XY}^{2}}{\hbar }\sqrt{\frac{\pi }{k_{B}TE_{r}^{\left(XY\right)}}}\exp\left[-\frac{{\left(\Delta E^{\left(XY\right)}+E_{r}^{\left(XY\right)}\right)}^{2}}{4k_{B}TE_{r}^{\left(XY\right)}}\right]$$

This capability was demonstrated by systematically modifying the parameters of the MSH model for the CPC_60_ triad conformation 3, as illustrated in Figure 15. The figure shows the Marcus parabolas for the six possible transitions among the three excited states and how their operating points shift in response to global changes in the model’s electronic coupling, reorganization energies or energy minima along with the nonadiabatic dynamics. For instance, when the reorganization energies were scaled by a factor of 1/9, the forward and backward rates between ππ* ↔ CT1 becomes more comparable as in Figure 15b3, thus the net population transfer rate from ππ* to CT1 gets greatly reduced as in Figure 15b2. Moreover, scaling the energy minima altered the thermodynamic driving forces and revealed that either increasing or decreasing this parameter could suppress net population transfer as in Figure 15c1,c2, but for different physical reasons. Increasing the driving forces pushed transitions into the deep normal and deep inverted regimes of the Marcus parabola, drastically lowering the absolute rate constants for both forward and backward reactions, as shown in Figure 15c3–c5. Conversely, decreasing the driving forces moved the system closer to the top of the parabolas, which increased the absolute rates but also brought them closer to equality, again resulting in a slow net population transfer.

**Figure 15:**
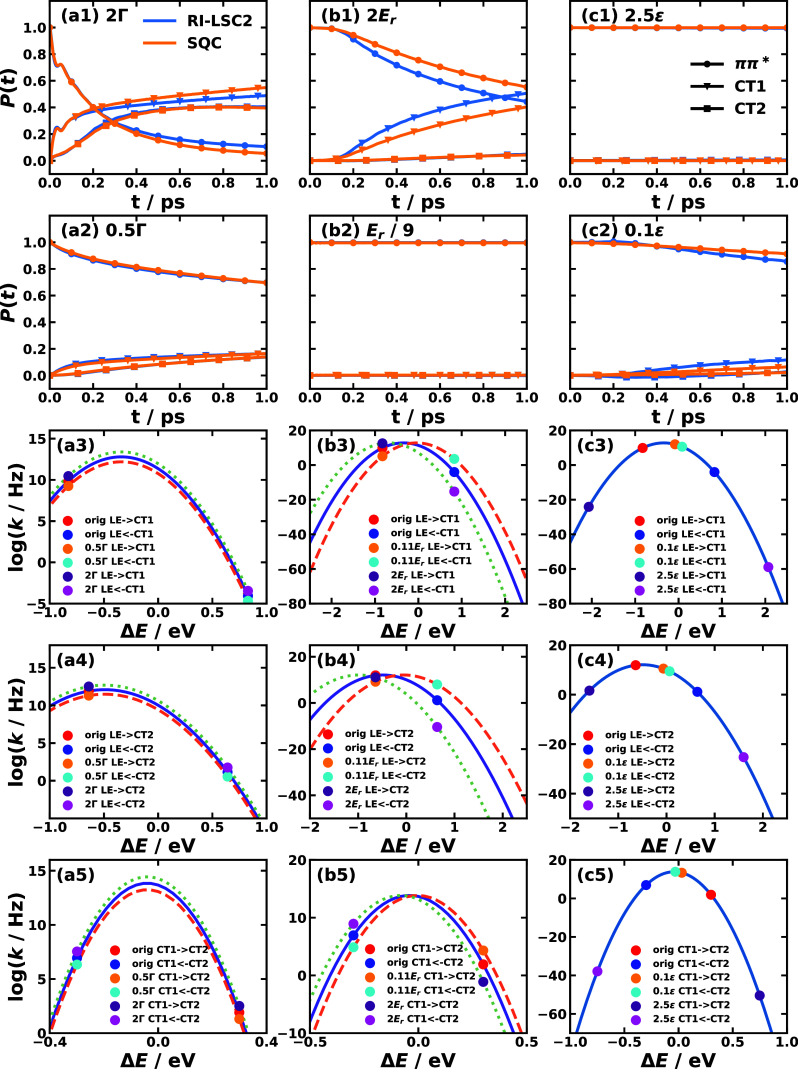
Nonadiabatic dynamics and Marcus parabolas for the modified MSH models based on CPC_60_ triad conformation 3 at 300 K. The left panels a1–a5 are based on applying factors of 2 and 0.5 on the electronic couplings, respectively. The middle panels in b1–b5 are on applying factors of 2 and 1/9 to the reorganization energies, respectively, and the right panels in c1–c5 are on applying factors of 2.5 and 0.1 to the energy minima parameters. Nonadiabatic dynamics obtained with RI-LSC2 and SQC methods for augmented and reduced parameters are shown in the first and second row, respectively. Different transitions with the Marcus rate constants are plotted in order of ππ*­(LE) ↔ CT1, ππ*­(LE) ↔ CT2, and CT1 ↔ CT2 from the third to the fifth row. Reproduced from ref 302 with permission of AIP Publishing.

While Marcus theory is widely used for the CT rate constant in condensed-phase systems,^85,277^ it is still based on a classical description of nuclear DOF and the assumption of static Gaussian donor–acceptor energy gap distribution. To incorporate important nuclear quantum effects (NQE), a fully quantum-mechanical FGR and its hierarchical approximations based on the LSC method have been developed.^105,137,226,287,299,301^ For each electronic transition, say from a given initial state |*X*⟩ to a final state |*Y*⟩, the MSH model could distill the relevant two-state spin-boson model, which has closed-form expressions for all these levels of approximations ranging from full quantum mechanical to more classical treatments, all the way to the Marcus-like expression.^105,137^ For example, the fully quantum-mechanical FGR rate constant for the spin-boson model (eq 10) coincides with the LSC approximation, which is based on Wigner sampling of the nuclear DOF and classical propagation on the average PES between the initial state and the final state:^105,137^

78 kX→Y=2ℏ2Re∫0∞dtC(t)

79 $$C\left(t\right)=\Gamma_{XY}^{2}{\mathrm{Tr}}_{N}\left[{\mathrm{\rho ̂}}_{X}^{\mathrm{eq}}e^{-i{Ĥ}_{Y}t/\hbar }e^{i{Ĥ}_{X}t/\hbar }\right]$$

where ${\mathrm{\rho ̂}}_{X}^{\mathrm{eq}}$ is the equilibrium nuclear density operator of |*X*⟩ state and Tr_*N*_(·) is trace over nuclear Hilbert space, and the exact/LSC expression for the TCF is given by^137^

80 $$\begin{array}{ll} C^{\mathrm{exact}/\mathrm{LSC}}\left(t\right) & =\Gamma_{XY}^{2}\exp\{-\frac{i\Delta E_{XY}t}{\hbar }-\overset{N}{\underset{j=1}{\sum }}\frac{\omega_{j}{\left(R_{j}^{\left(XY\right)}\right)}^{2}}{2\hbar }\\ & \times \left[\coth\left(\frac{\beta \hbar \omega_{j}}{2}\right)\left(1-\cos\left(\omega_{j}t\right)\right)+i\sin\left(\omega_{j}t\right)\right]\} \end{array}$$

At the classical end of the hierarchy of FGR approximations, the Marcus-like expression for the CT rate constant is

81 $$k_{X\to Y}^{M}=\frac{\Gamma_{XY}^{2}}{\hbar }\sqrt{\frac{2\pi }{\sigma_{XY}^{2}}}\exp\left[-\frac{\langle U_{XY}\rangle^{2}}{2\sigma_{XY}^{2}}\right]$$

where *U*_*XY*_ = *V*_*X*_ – *V*_*Y*_ is the energy gap with average ⟨*U*_*XY*_⟩ and standard deviation $\sigma_{XY}=\sqrt{\left\langle U_{XY}^{2}\right\rangle -\langle U_{XY}\rangle^{2}}$ . Comparing eqs 81 and 77, we have the correspondence $E_{r}^{\left(XY\right)}=\sigma_{XY}^{2}/\left(2k_{B}T\right)=-\Delta E^{\left(XY\right)}-\left\langle U_{XY}\right\rangle$ (eq 41). The CT rate constant calculation is particularly useful to estimate the importance of NQE by comparing the hierarchical FGR approximations and also to predict the time scale of an electronic transition, which might be too slow to perform any nonadiabatic dynamics, like in microseconds.

While rate constant theory is powerful for equilibrium or slow processes, photoinduced dynamics are inherently nonequilibrium phenomena. The NE-FGR^113,134,226,293,296,301,303–307^ provides the appropriate theoretical tool for these situations, yielding a time-dependent rate coefficient, *k*_*D → A*_(*t*), that captures the transient effects of nuclear relaxation following photoexcitation. For a nonequilibrium transition |*D*⟩ → |*A*⟩ with initial nuclear state sampled from the ground state, the population of |*D*⟩ is

82 $$P_{D}\left(t\right)\approx \exp\left[-\int_{0}^{t}dt^{\prime }k_{D\to A}\left(t^{\prime }\right)\right]$$

where the time-dependent NE-FGR rate is

83 kD→A(t)=2ℏ2Re∫0tdτC(t,τ)

84 $$C\left(t,\tau \right)=\Gamma_{DA}^{2}{\mathrm{Tr}}_{N}\left[e^{-i{Ĥ}_{D}t/\hbar }\mathrm{\rho ̂}\left(0\right)e^{i{Ĥ}_{D}t/\hbar }{\mathrm{V̂}}_{DA}e^{-i{Ĥ}_{A}\tau /\hbar }{\mathrm{V̂}}_{AD}e^{i{Ĥ}_{D}\tau /\hbar }\right]$$

where the nuclear density operator has evolved for time *t* on the donor PES starting from an arbitrary *ρ̂*(0). The time scale of the transient NE-FGR rate *k*_*D → A*_(*t*) is typically chosen such that a plateau value is reached. For each time *t*, the second variable τ in TCF *C*(*t*,τ) is scanned from 0 to *t*. It is important to distinguish these TCFs from those used to parametrize the model: while the energy-gap TCFs $\left(C_{UU}^{\left(XY\right)}\left(t\right)\right)$ are obtained from all-atom simulations to construct the MSH Hamiltonian, the NE-FGR TCFs *C*(*t*,τ) can be subsequently calculated analytically from the resulting MSH model’s parameters. The harmonic nature of the model thus allows for an exact quantum-mechanical treatment of the NE-FGR rate. Figure 16 shows two distinct and opposing transient behaviors in the NE-FGR time-dependent rate for two separate transitions of the CPC_60_ triad’s bent conformation, i.e., ππ → CT1 and ππ* → CT2, which are started with the nuclear initial state in equilibrium with the ground state PES. Here, the MSH model (labeled as “model 3” in ref 106) and the MSH model with the last nuclear subspace associated with initial nuclear sampling truncated (labeled as “model 2” in ref 106) proved highly effective. For the ππ* → CT1 transition, the rate coefficient is initially very high and then decays significantly over the first 2 ps. The physical picture is that the vertical photoexcitation from the ground state places the system in a nuclear configuration that is fortuitously close to the crossing seam of the ππ* and CT1 PESs, leading to an enhanced initial transfer rate; subsequent structural relaxation on the ππ* PES moves the system away from this favorable region, causing the rate to decrease. In contrast, for the ππ* → CT2 transition, the rate coefficient is initially small and slightly increases with time. In this case, the vertically excited geometry is a bit further from the relevant crossing seam for the CT2 state than the ππ* PES minimum, and the nuclear relaxation brings the system toward the ππ* minimum, a more favorable configuration for transfer, thus increasing the rate. The ability of the MSH model to accurately capture these complex, opposing transient effects, which were also observed in all-atom simulations, underscores its power in representing multistate potential energy landscape.

**Figure 16:**
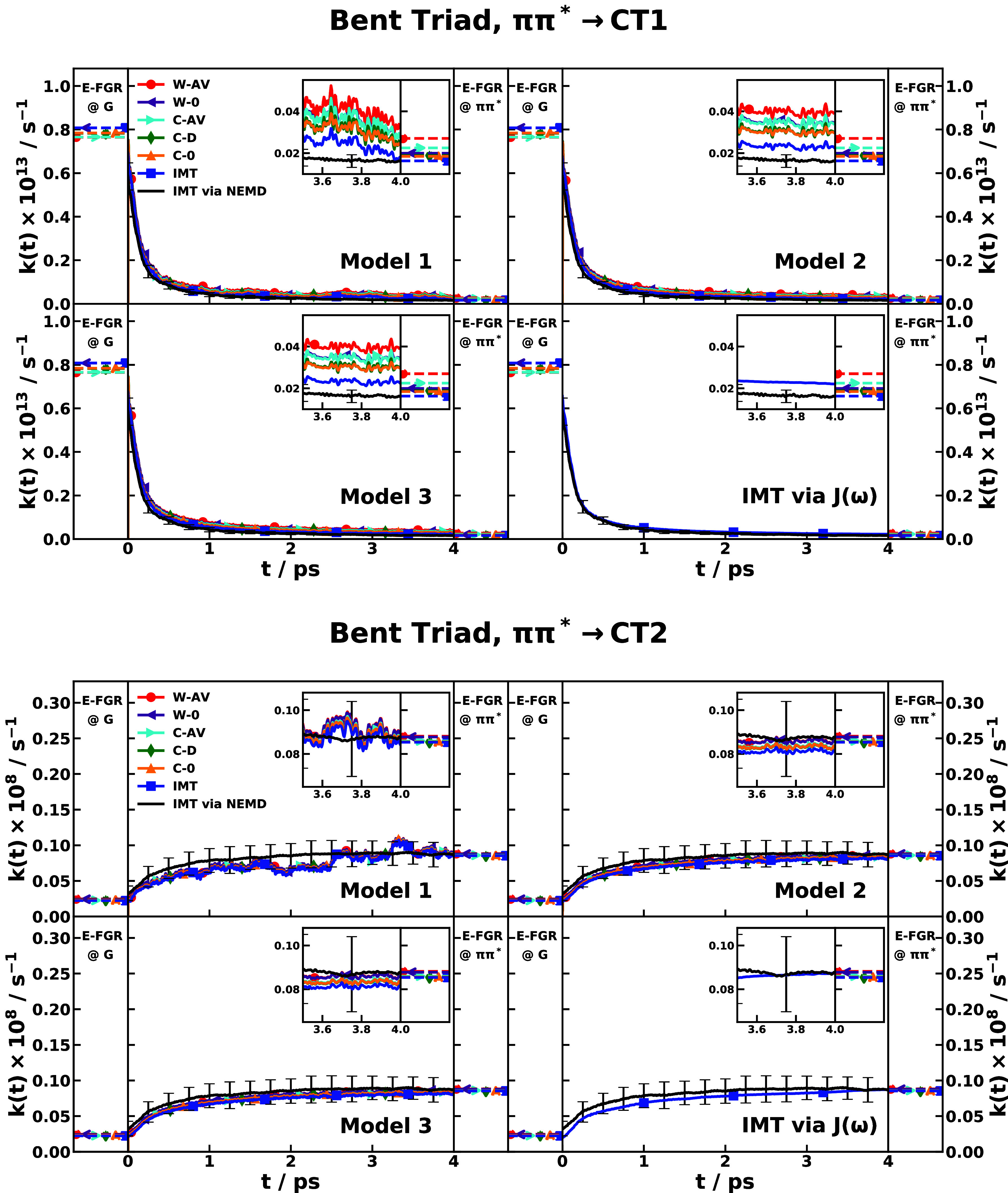
Time-dependent NE-FGR charge transfer rate for ππ* → CT1 (top) and ππ* → CT2 (bottom) transitions for CPC_60_ conformation 1 (bent triad) compared with FGR rate constants sampled on equilibrated ground (G) and ππ* states, calculated with different model Hamiltonians. Here, Model 1 is the randomly flipping model in 1 dimension, Model 2 is the projected MSH model in 1 dimension, and Model 3 is the MSH model. The instantaneous Marcus theory via spectral density [IMT via *J*(ω)] was obtained using eqs 58 and 59 in ref 106. Reproduced from ref 106 with permission of AIP Publishing.

The initial application of NE-FGR to these models was limited to describing a given donor–acceptor transition with nonequilibrium nuclear initial condition. More recently, we have generalized the NE-FGR formalism to a full multistate network within the MSH framework. This significant advance moves beyond calculating a single *k*_*D → A*_(*t*) and instead formulates a set of coupled Pauli’s master equations for the *F* electronic populations:^77,78^

85 ddtPk(t)=∑j≠kFkj→k(t)Pj(t)−∑j≠kFkk→j(t)Pk(t),(k=1,...,F)

where *P*_*k*_(*t*) is the population of state |*k*⟩. In this generalized NE-FGR approach,^134^ the time-dependent rate coefficients, *k*_*j→k*_(*t*), for all possible forward and backward transitions are computed simultaneously using NE-FGR with a common, nonequilibrium initial nuclear state:

86 kj→k(t)=2ℏ2Re∫0tdτCjk,kj(t,τ)

87 $$C_{jk,kj}\left(t,\tau \right)=\left\langle {Ṽ}_{jk}\left(t\right){Ṽ}_{kj}\left(\tau \right)\right\rangle$$

where ${Ṽ}_{jk}\left(t\right)=e^{i{Ĥ}_{j}t/\hbar }{\mathrm{V̂}}_{jk}e^{-i{Ĥ}_{k}t/\hbar },\left(j\neq k\right)$ and ⟨•⟩ = Tr_*N*_[*ρ̂*(0)•]. For the MSH Hamiltonian, the closed-form expression for *C*_*jk,kj*_(*t*,τ) follows the general *C*_*jk,mn*_(*t*,τ) in eq 109.^134,302,308^

A very important extension of the LSC NE-FGR is the instantaneous Marcus theory (IMT),^301,303,309,310^ which provides an intuitive and efficient framework for describing nonequilibrium charge transfer. Derived from the classical limit of NE-FGR, its accuracy depends on the assumptions that nuclear motion is classical and slow compared to electronic dephasing, and that the energy gap follows a time-dependent Gaussian distribution. The IMT expression for the time-dependent rate is

88 $$k_{j\to k}^{\mathrm{IMT}}\left(t\right)=\frac{|\Gamma_{jk}{|}^{2}}{\hbar }\sqrt{\frac{2\pi }{\sigma_{jk}^{2}\left(t\right)}}\exp\left[-\frac{{\left(\overset{―}{U_{jk}\left(t\right)}\right)}^{2}}{2\sigma_{jk}^{2}\left(t\right)}\right]$$

where $\overset{―}{U_{jk}\left(t\right)}$ and $\sigma_{jk}^{2}\left(t\right)$ are the nonequilibrium average and variance of the energy gap *U*_*jk*_. This theory is particularly powerful for the MSH model, as its harmonic nature allows for the analytical derivation of the IMT parameters. Specifically, the time-dependent energy gap average $\overset{―}{U_{jk}\left(t\right)}$ can be expressed analytically, $\overset{―}{U_{jk}\left(t\right)}=\left\langle U_{jk}\right\rangle +\overset{N_{n}}{\underset{i=1}{\sum }}\omega_{i}^{2}D_{i}^{jk}D_{i}^{jg}\cos\left(\omega_{i}t\right)$ , where $D_{i}^{jk}$ is the PES minima shift between states *j* and *k* along the *i*-th mode and *g* is the ground state, while the variance $\sigma_{jk}^{2}\left(t\right)$ becomes a time-independent constant proportional to the reorganization energy, i.e., $\sigma_{jk}^{2}\left(t\right)=2k_{B}TE_{r}^{\left(jk\right)}$ .^310^ This analytical tractability makes the MSH model an ideal system for rigorously testing the IMT framework itself against more accurate quantum mechanical methods like NE-FGR for the same MSH model. Our results confirm that for the MSH model, population dynamics predicted by IMT show excellent agreement with full quantum-mechanical NE-FGR calculations, validating that IMT accurately captures the essential nonequilibrium physics when the underlying energy gap statistics are described by time-dependent Gaussian. The framework is generalized to multistate systems by calculating these pathway-specific IMT rates and using them to solve a set of coupled Pauli’s master equations (eq 85), which allows for the simulation of the complete population dynamics across the entire network of electronic states.

#### 5.3.4 Applications to Perturbative Quantum Master Equation

The MSH framework also serves as an ideal platform for developing and benchmarking perturbative quantum master equations (QMEs), which offer a powerful alternative to semiclassical methods for simulating nonadiabatic dynamics.^77,78,233,311^ By treating the off-diagonal electronic couplings as a perturbation, one can derive equations of motion for the electronic population or the reduced density matrix (RDM) σ̂(*t*) = Tr_*N*_[*ρ̂*(*t*)] in either a time-convolution (TC) or time-convolutionless (TCL) form.^94,302,308,312–315^ Consider a general multistate Hamiltonian

89 $$Ĥ=\overset{F}{\underset{j=1}{\sum }}{Ĥ}_{j}\left|j\right\rangle \left\langle j\right|+\overset{F}{\underset{j\neq k}{\sum }}{\mathrm{V̂}}_{jk}\left|j\right\rangle \left\langle k\right|={Ĥ}_{0}+{Ĥ}_{I}$$

we denote the Liouville superoperators as L(•)=[Ĥ,•] , $L_{0}\left(•\right)=\left[{Ĥ}_{0},•\right]$ , $L_{I}\left(•\right)=\left[{Ĥ}_{I},•\right]$ . Starting from interaction-picture quantum Liouville equation with a general projection superoperator P (satisfying idempotence condition $P^{2}=P$ ) and its complementary projection superoperator Q=1−P one can arrive at the formally exact Nakajima-Zwanzig generalized quantum master equation (NZ-GQME)^77,191,194,230–248^ for the evolution of projected density matrix Pρ̃(t) :

90 $$\frac{d}{dt}P\mathrm{\rho ̃}\left(t\right)=-\frac{i}{\hbar }P{\mathrm{L̃}}_{I}\left(t\right)P\mathrm{\rho ̃}\left(t\right)-\int_{0}^{t}d\tau {\mathrm{K̃}}_{I}^{\mathrm{NZ}}\left(t,\tau \right)P\mathrm{\rho ̃}\left(\tau \right)+{Ĩ}^{\mathrm{NZ}}\left(t,0\right)$$

Here, ${\mathrm{K̃}}_{I}^{\mathrm{NZ}}\left(t,\tau \right)$ is the memory kernel superoperator, which is the core of QME and encodes the time-correlated influence of the bath and historical states on the electronic system’s evolution and it is defined as

91 $${\mathrm{K̃}}_{I}^{\mathrm{NZ}}\left(t,\tau \right)=\frac{1}{\hbar^{2}}P{\mathrm{L̃}}_{I}\left(t\right){\mathrm{G̃}}_{Q}\left(t,\tau \right)Q{\mathrm{L̃}}_{I}\left(\tau \right)P$$

where the interaction-picture Q -projected-propagator is

92 $${\mathrm{G̃}}_{Q}\left(t,\tau \right)=\exp_{+}\left[-\frac{i}{\hbar }\int_{\tau }^{t}dsQ{\mathrm{L̃}}_{I}\left(s\right)\right]$$

In the interaction picture, the density operator is written as $\mathrm{\rho ̃}\left(t\right)=e^{i{Ĥ}_{0}t/\hbar }\mathrm{\rho ̂}\left(t\right)e^{-i{Ĥ}_{0}t/\hbar }$ and the perturbation Liouville superoperator is written as ${\mathrm{L̃}}_{I}\left(t\right)=e^{itL_{0}/\hbar }L_{I}e^{-itL_{0}/\hbar }$ . ${Ĩ}^{\mathrm{NZ}}\left(t,0\right)$ is the inhomogeneous term, which vanishes if the initial condition can be written as direct product of the electronic and nuclear parts: *ρ̂*(0) = σ̂(0) ⊗ *ρ̂*_*N*_(0). Applying the second-order perturbation theory to the NZ-GQME, one obtains the TC QME:

93 $$\frac{d}{dt}P\mathrm{\rho ̃}\left(t\right)=-\frac{i}{\hbar }P{\mathrm{L̃}}_{I}\left(t\right)P\mathrm{\rho ̃}\left(t\right)-\frac{1}{\hbar^{2}}\int_{0}^{t}d\tau P{\mathrm{L̃}}_{I}\left(t\right)Q{\mathrm{L̃}}_{I}\left(\tau \right)P\mathrm{\rho ̃}\left(\tau \right)$$

Furthermore, the Shibata–Takahashi–Hashitsume equation^316–318^ reduces to TCL QME under the second-order perturbative treatment for ${\mathrm{L̃}}_{I}\left(t\right)$ :

94 $$\frac{d}{dt}P\mathrm{\rho ̃}\left(t\right)=-\frac{i}{\hbar }P{\mathrm{L̃}}_{I}\left(t\right)P\mathrm{\rho ̃}\left(t\right)-\frac{1}{\hbar^{2}}\left[\int_{0}^{t}d\tau P{\mathrm{L̃}}_{I}\left(t\right)Q{\mathrm{L̃}}_{I}\left(\tau \right)\right]P\mathrm{\rho ̃}\left(t\right)$$

Choosing the population-only and the full-RDM projection superoperator P :^302,308,314^

95 $$P\left(•\right)=\underset{j}{\sum }\left|j\right\rangle \left\langle j\right|⊗{\mathrm{\rho ̂}}_{N}\left(0\right)\mathrm{Tr}\left[\left|j\right\rangle \langle j|•\right]$$

96 $$P\left(•\right)={\mathrm{\rho ̂}}_{N}\left(0\right)⊗{\mathrm{Tr}}_{N}\left(•\right)$$

gives rise to two different TC/TCL QMEs.

First, applying the population-only P defined in eq 95 to eqs 93 and 94, we have the TC and TCL QMEs for populations ( σ̃ _*kk*_ = σ̂_*kk*_):

97 $$\frac{d}{dt}{\mathrm{\sigma ̃}}_{kk}\left(t\right)=-\frac{1}{\hbar^{2}}\overset{F}{\underset{j=1}{\sum }}\left[\int_{0}^{t}d\tau K_{kk,jj}^{\prime }\left(t,\tau \right)\right]{\mathrm{\sigma ̃}}_{jj}\left(t\right)$$

98 $$\frac{d}{dt}{\mathrm{\sigma ̃}}_{kk}\left(t\right)=-\frac{1}{\hbar^{2}}\overset{F}{\underset{j=1}{\sum }}\int_{0}^{t}d\tau K_{kk,jj}^{\prime }\left(t,\tau \right){\mathrm{\sigma ̃}}_{jj}\left(\tau \right)$$

Here, the memory kernels are given by

99 Kkk,jj′(t,τ)=2Reδkj∑l≠k,jF⟨Ṽkl(t)Ṽlk(τ)⟩−2Re⟨Ṽjk(t)Ṽkj(τ)⟩

where ${Ṽ}_{jk}\left(t\right)={\mathrm{Tr}}_{e}\left[\left|k\right\rangle \langle j|{\mathrm{H̃}}_{I}\left(t\right)\right]=e^{i{Ĥ}_{j}t/\hbar }{\mathrm{V̂}}_{jk}e^{-i{Ĥ}_{k}t/\hbar }$ and their TCFs are defined as

100 $$C_{jk,kj}\left(t,\tau \right)=\left\langle {Ṽ}_{jk}\left(t\right){Ṽ}_{kj}\left(\tau \right)\right\rangle$$

Second, applying the full-RDM P defined in eq 96 to eqs 93 and 94, we have the TC and TCL QMEs for RDM σ̃ ­(*t*):

101 $$\frac{d}{dt}\mathrm{\sigma ̃}\left(t\right)=-\frac{i}{\hbar }\left\langle {\mathrm{L̃}}_{I}\left(t\right)\right\rangle \mathrm{\sigma ̃}\left(t\right)-\frac{1}{\hbar^{2}}\int_{0}^{t}d\tau K\left(t,\tau \right)\mathrm{\sigma ̃}\left(\tau \right)$$

102 $$\frac{d}{dt}\mathrm{\sigma ̃}\left(t\right)=-\frac{i}{\hbar }\left\langle {\mathrm{L̃}}_{I}\left(t\right)\right\rangle \mathrm{\sigma ̃}\left(t\right)-\frac{1}{\hbar^{2}}\left[\int_{0}^{t}d\tau K\left(t,\tau \right)\right]\mathrm{\sigma ̃}\left(t\right)$$

Here, the memory kernels have more nonvanishing terms:

103 Kjk,mn(t,τ)=δkn∑s≠j,m⟨δṼjs(t)δṼsm(τ)⟩+δjm∑s≠k,n⟨δṼks(t)δṼsn(τ)⟩*−⟨δṼnk(t)δṼjm(τ)⟩−⟨δṼmj(t)δṼkn(τ)⟩*

where the TCFs are ⟨δ*Ṽ*_*jk*_(*t*)­δ*Ṽ*_*mn*_(τ)⟩ = ⟨*Ṽ*_*jk*_(*t*)*Ṽ*_*mn*_(τ)⟩ – ⟨*Ṽ*_*jk*_(*t*)⟩ ⟨*Ṽ*_*mn*_(τ)⟩ with the fluctuations δ*Ṽ*_*jk*_(*t*) = *Ṽ*_*jk*_(*t*) − ⟨*Ṽ*_*jk*_(*t*)⟩, and the elements of $\left\langle {\mathrm{L̃}}_{I}\left(t\right)\right\rangle$ are given by

104 $$\langle {\mathrm{L̃}}_{I}\left(t\right)\rangle_{jk,mn}=\delta_{kn}\left\langle {Ṽ}_{jm}\left(t\right)\right\rangle -\delta_{jm}\left\langle {Ṽ}_{nk}\left(t\right)\right\rangle$$

Thus, for population-only TC/TCL QME, one needs the inputs of *C*_*jk,kj*_(*t*,τ) for memory kernel $K_{kk,jj}^{\prime }\left(t,\tau \right)$ in eq 99; for full-RDM TC/TCL QME, one needs the following TCFs *C*_*jk,mn*_(*t*,τ) and *C*_*jk*_(*t*) for memory kernel K(t,τ) and elements of $\left\langle {\mathrm{L̃}}_{I}\left(t\right)\right\rangle$ in eqs 103 and 104, respectively:

105 $$C_{jk,mn}\left(t,\tau \right)=\left\langle {Ṽ}_{jk}\left(t\right){Ṽ}_{mn}\left(\tau \right)\right\rangle$$

106 $$C_{jk}\left(t\right)=\left\langle {Ṽ}_{jk}\left(t\right)\right\rangle$$

A key advantage of the MSH model is that these complex kernels, which are computationally prohibitive for all-atom systems, can be derived in a closed, analytical form. If we set $\left\{S_{i}^{\left(XY\right)}\right\}≡0$ when *X* ≥ *Y* in the MSH Hamiltonian in eq 23 such that the nuclear Hamiltonian of *X*-th state (X = 1,···,*F*) is expressed as

107 $${Ĥ}_{X}=\overset{F-1}{\underset{a=1}{\sum }}\overset{N}{\underset{i=1}{\sum }}\left[\frac{{\mathrm{P̂}}_{a,i}^{2}}{2}+\frac{1}{2}\omega_{i}^{2}{\left({\mathrm{R̂}}_{a,i}-S_{i}^{\left(aX\right)}\right)}^{2}\right]+\varepsilon_{X}$$

Denoting $D_{a,i}^{\left(XY\right)}=S_{i}^{\left(aX\right)}-S_{i}^{\left(aY\right)}$ and *ℏ*ω_*XY*_ = ε_*X*_ – ε_*Y*_, we can express the exact quantum-mechanical and the coinciding LSC approximation for the TCFs as below:^302^

108 Cjkexact/LSC(t)=Γjkexp{iωjkt+∑a=1F−1∑i=1Nωi(Da,i(jk))22ℏcoth(βℏωi2)[cos(ωit)−1]+i·∑a=1F−1∑i=1Nωi2ℏ((Si(aj))2−(Si(ak))2)sin(ωit)}

109 $$\begin{array}{ll} C_{jk,mn}^{\mathrm{exact}/\mathrm{LSC}}\left(t,\tau \right) & =\Gamma_{jk}\Gamma_{mn}\exp\{i\omega_{jk}t+i\omega_{mn}\tau +\overset{F-1}{\underset{a=1}{\sum }}\overset{N}{\underset{i=1}{\sum }}\frac{\omega_{i}}{2\hbar }\coth\left(\frac{\beta \hbar \omega_{i}}{2}\right)\\ & \times [-D_{a,i}^{\left(jk\right)}D_{a,i}^{\left(mn\right)}\cos\left(\omega_{i}t-\omega_{i}\tau \right)+D_{a,i}^{\left(jk\right)}\left(D_{a,i}^{\left(jk\right)}+D_{a,i}^{\left(mn\right)}\right)\cos\left(\omega_{i}t\right)\\ & +D_{a,i}^{\left(mn\right)}\left(D_{a,i}^{\left(jk\right)}+D_{a,i}^{\left(mn\right)}\right)\cos\left(\omega_{i}\tau \right)-{\left(D_{a,i}^{\left(jk\right)}\right)}^{2}-{\left(D_{a,i}^{\left(mn\right)}\right)}^{2}-D_{a,i}^{\left(jk\right)}D_{a,i}^{\left(mn\right)}]\\ & +i\cdot \overset{F-1}{\underset{a=1}{\sum }}\overset{N}{\underset{i=1}{\sum }}\frac{\omega_{i}}{2\hbar }[D_{a,i}^{\left(jk\right)}D_{a,i}^{\left(mn\right)}\sin\left(\omega_{i}t-\omega_{i}\tau \right)\\ & +D_{a,i}^{\left(jk\right)}\left(S_{i}^{\left(aj\right)}+S_{i}^{\left(ak\right)}-S_{i}^{\left(am\right)}+S_{i}^{\left(an\right)}\right)\sin\left(\omega_{i}t\right)\\ & +D_{a,i}^{\left(mn\right)}\left(S_{i}^{\left(aj\right)}-S_{i}^{\left(ak\right)}+S_{i}^{\left(am\right)}+S_{i}^{\left(an\right)}\right)\sin\left(\omega_{i}\tau \right)]\} \end{array}$$

and the *C*_*jk,kj*_(*t*,τ) is a special case of eq 109.^134,302,308^

For example, Figure 17 illustrates the 81 memory kernels, $K_{jk,mn}\left(t,\tau \right)$ at a fixed *t* = 0.02 ps for the three-excited-state MSH model of the CPC_60_ triad conformation 3. The kernels exhibit a rapid decay on a time scale of less than 100 fs (see $K_{jk,mn}\left(t,\tau \right)$ at fixed *t* = 0.25 ps in Figure S15 of ref 302), indicating that the environment-induced memory effects are short-lived. Furthermore, only a specific subset of kernels possesses significant magnitude, corresponding to the dominant diagonal self-evolution $K_{jk,jk}$ and off-diagonal population transfer $K_{jj,kk}$ , providing a direct view into the primary mechanisms of quantum evolution. The other small-amplitude cross terms of $K_{jk,mn}$ , such as $K_{00,12}$ , $K_{00,21}$ , $K_{01,12}$ , $K_{01,22}$ , represent the influence between population and coherence, which is even shorter lived than the dominant kernels.

**Figure 17:**
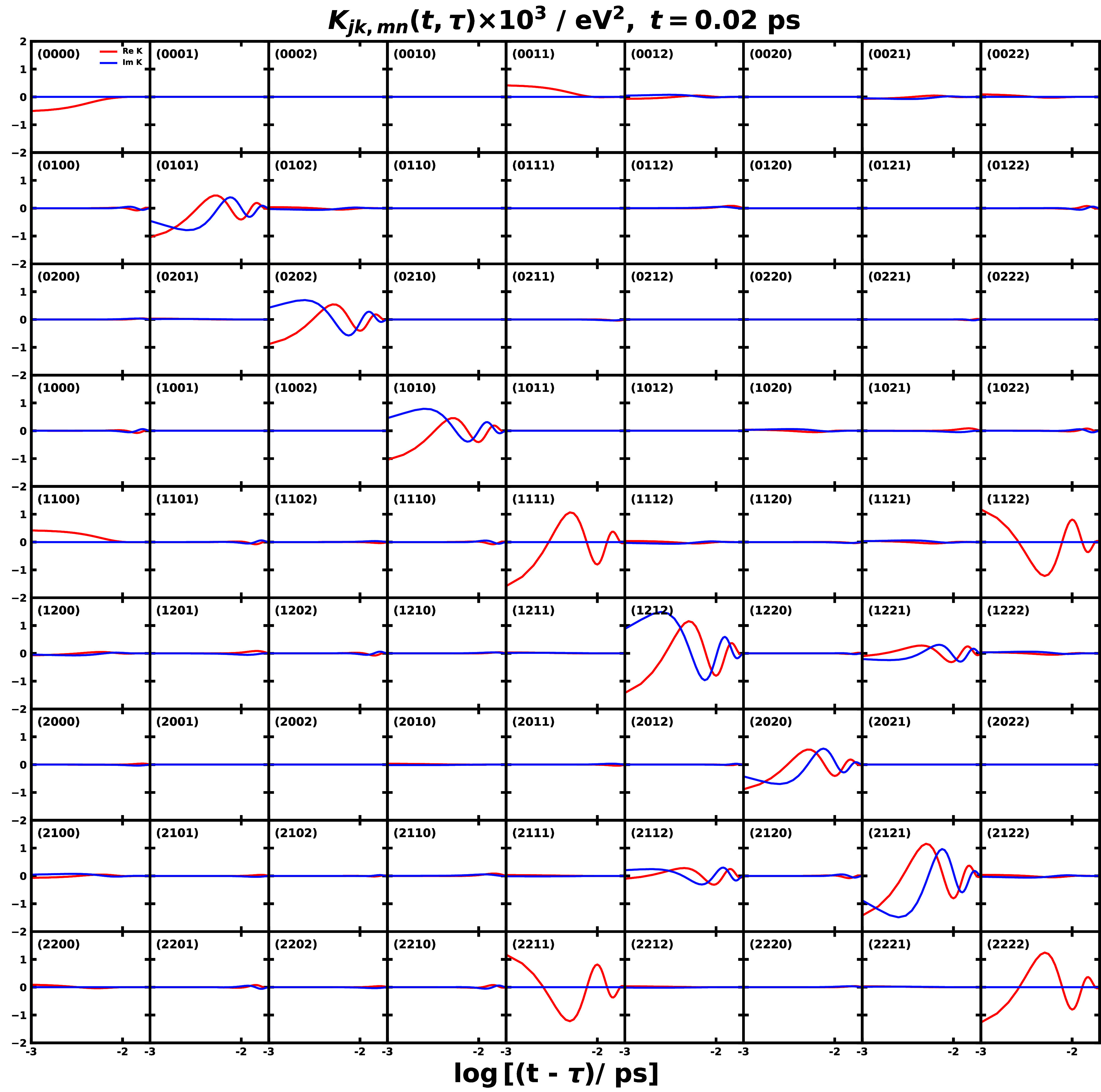
Full 81 kernels $K_{jk,mn}\left(t,\tau \right)$ with a fixed *t* = 0.02 ps for the MSH model of CPC_60_ triad conformation 3 at 300 K on the exact quantum mechanical level. Reproduced from ref 302 with permission of AIP Publishing.

With these analytical kernels, we can directly compare the dynamics predicted by different QME formalisms against those from nonadiabatic semiclassical methods for the same underlying MSH model. Figure 18 presents such a comparison for the RDM dynamics of the triad conformation 3’s MSH model. A general observation is that the QMEs, both TC and TCL versions with either full-RDM or population-only projections, predict a faster initial population transfer than the LSC and SQC methods. While all four QME variants yield broadly similar population dynamics, demonstrating a degree of robustness, their predictions for the electronic coherences show more pronounced differences when compared to the semiclassical results. The coherences are significant for nearly a picosecond, and while the overall decay trends are similar, the QMEs often predict different magnitudes and oscillatory patterns, highlighting the distinct theoretical underpinnings of perturbative versus semiclassical treatments of the coupled electronic-nuclear evolution.

**Figure 18:**
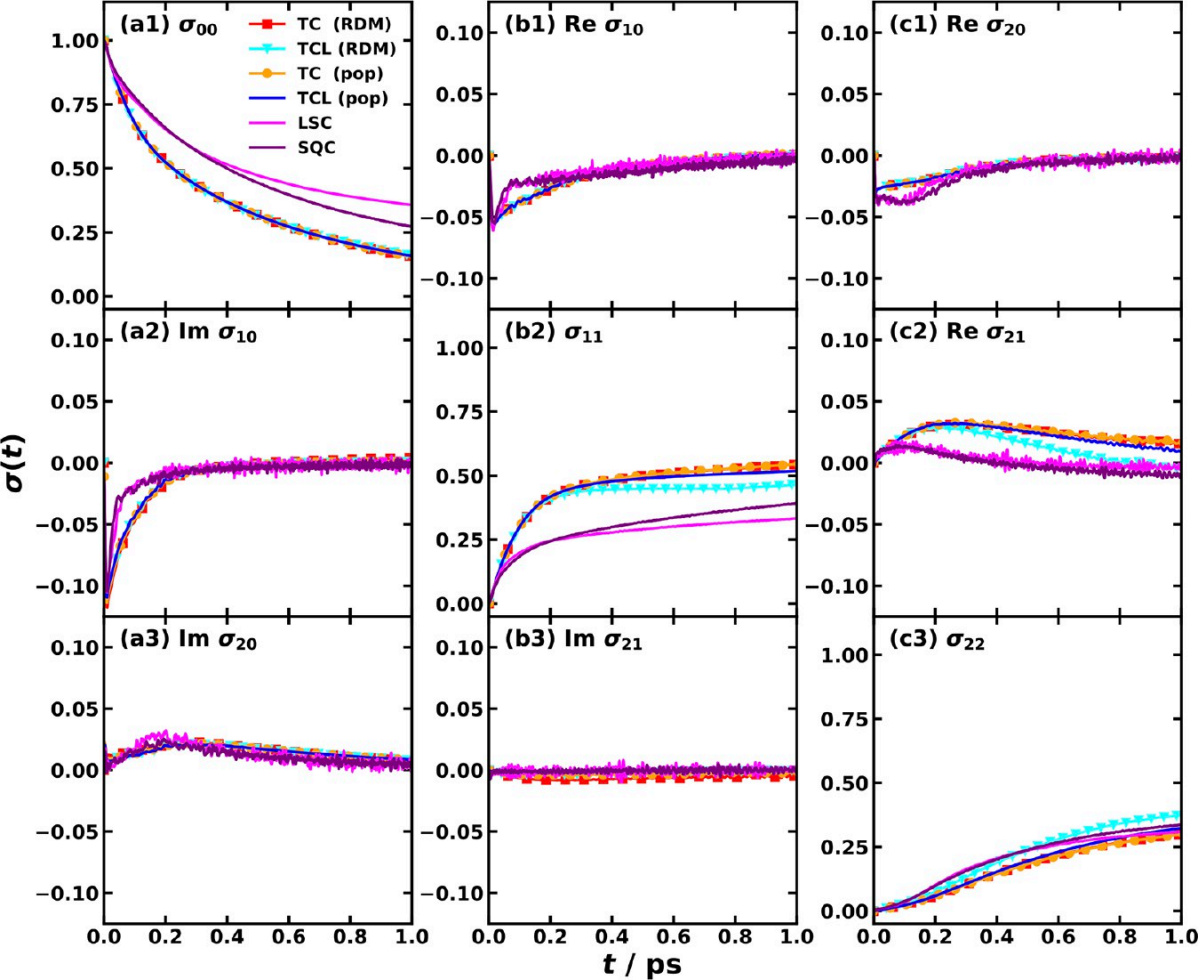
Full RDM dynamics for the MSH model of CPC_60_ triad conformation 3 at 300 K obtained with exact quantum-mechanical kernels in the population-only full-RDM time-convolution (TC) and time-convolutionless (TCL) QMEs, which are compared with RI-LSC2 and SQC with triangle window methods. Reproduced from ref 302 with permission of AIP Publishing.

The analytical tractability of the MSH model also allows for a systematic assessment of various semiclassical approximations within the QME kernels themselves. This hierarchical approach, shown for the CPC_60_ triad conformation 3 in Figure 19, involves comparing the exact quantum kernel to approximations based on Wigner (W) or classical (C) nuclear sampling, combined with different treatments of the nuclear dynamics during the quantum coherence period: propagation on an average PES (AV), on the ground-state PES (G), or no propagation at all (0). Thus, the LSC approximation is labeled as W-AV. For the triad at 300 K, the results from Wigner and classical sampling are nearly identical, indicating that NQE are not significant in this system. However, the different treatments of the coherence dynamics lead to noticeable variations, particularly for the TCL QME, where the C-0 and W-0 approximations deviate from the reference exact/W-AV dynamics.

**Figure 19:**
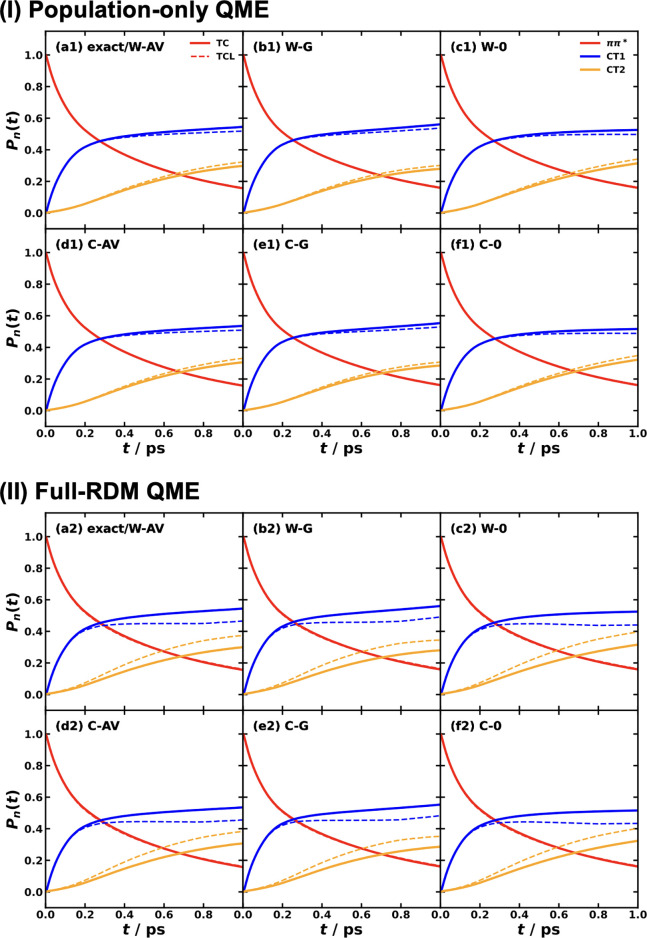
Hierarchy of semiclassical approximations for the kernels of population-only (top) and full-RDM (bottom) TC and TCL QMEs for PICT in CPC_60_ triad conformation 3 at 300 K. Reproduced from refs 308 and 302 with permission of AIP Publishing.

This hierarchy of semiclassical approximations reveals even more pronounced differences when applied to the EET dynamics in the *P. aestuarii* FMO complex, as shown in Figure 20. Here, the TC QME is clearly superior to the TCL QME in capturing the initial coherent population oscillations between BChl sites, with the TCL approach yielding an overdamped response regardless of the kernel used. Importantly, for the FMO complex, the W-0 and C-0 approximations, which neglect nuclear dynamics during the coherence period, completely fail to reproduce the short-time oscillatory features of the EET process. This result demonstrates that a proper description of the nuclear evolution during the lifetime of electronic coherence is essential for capturing EET dynamics, a requirement that was less stringent for the faster, charge-transfer-driven dynamics of the triad. This comparative analysis underscores the power of the MSH model as a versatile testbed: it not only allows for rigorous benchmarking of different QME theories but also provides deep physical insights into which specific dynamical approximations are valid for different types of nonadiabatic processes.

**Figure 20:**
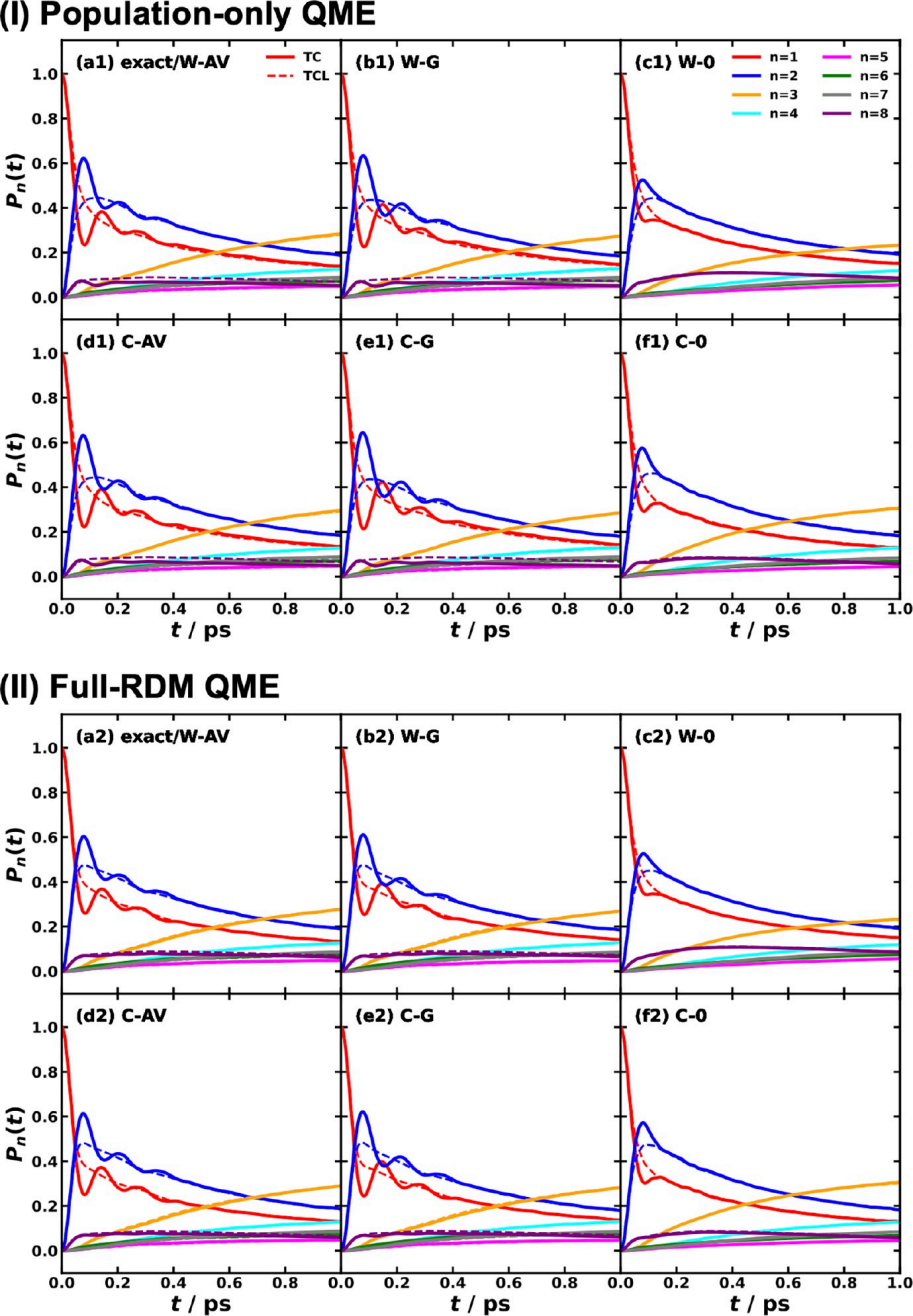
Hierarchy of semiclassical approximations for the kernels of population-only (top) and full-RDM (bottom) TC and TCL QMEs for EET in 8-site FMO complex of *P. aestuarii* at 300 K. Reproduced from refs 308 and 302 with permission of AIP Publishing.

## 6 Discussion

### 6.1 Distinguishment of MSH/MRC Framework from Other Correlated Bath Models

While the traditional Frenkel exciton or IBH model neglects bath correlations,^110^ more advanced frameworks have been developed to include the correlated bath effect within the local bath bases. A prominent example is the extended Frenkel (EF) exciton model,^115,319^ developed by Burghardt and coworkers to describe multichromophoric systems that feature both Frenkel excitonic (EX) and charge-transfer states. The EF model provides an important point of comparison that highlights the unique structural and philosophical underpinnings of the MSH framework.

The EF Hamiltonian of *M* chromophores is constructed within a system-bath formalism, where the bath is composed of *M* independent sets of *N* local normal modes, with one set associated with each of the *M* molecular sites in the aggregate, so the total number of nuclear DOF is *N*_*n*_ = *MN*. The electronic states include *M* local EX states, |EX_*a*_⟩ with *a* = 1,···, *M*, and up to *M*(*M* – 1) CT states, |CT_*a,b*_⟩ for positively charged site *a* and negatively charged site *b* (*b* ≠ *a*), so the total number of electronic states is *N*_*e*_ = *M* ^2^. If we label the electronic states {|EX_1_⟩,···,|EX_*M*_⟩,|CT_1,2_⟩,···,|CT_*M*‑1,*M*_⟩,|CT_2,1_⟩,···,|CT_*M*‑1,*M*_⟩} as {|1⟩,|2⟩,···,|*N*_*e*_⟩}, the EF Hamiltonian can be expressed generally as^115^

Ĥ=Ĥsys+Ĥbath+ĤbsĤsys=∑s=1Neεs|s⟩⟨s|+∑u≠sNeΓus|u⟩⟨s|Ĥbath=∑a=1M∑j=1N(P̂a,j22+12ωj2R̂a,j2)Ĥbs=∑s=1Ne∑j=1Ncs,jR̂a(s),j|s⟩⟨s| 110

Here, the nuclear coordinates are **R̂** = (**R̂**_1_,···, **R̂**_*M*_) with the nuclear coordinates in the *a*-th subspace **R̂**_*a*_=(*R̂*_*a,1*_ ...,*R̂*_*a,N*_) representing the local normal modes for molecular site *a* (*a* = 1,···,*M*), usually obtained with electronic structure calculation at the ground-state optimized geometry. The momenta of the *a*-th local bath modes are denoted as **P̂**_*a*_=(*P̂*_*a,1*_ ...,*P̂*_*a,N*_). The nuclear space and the normal-mode frequencies {ω_*j*_|*j* = 1,···, *N*} of the EF model are the same as the Frenkel exciton model or the state-independent IBH model (eq 21). In the system Hamiltonian, *Ĥ*_sys_, the nonvanishing terms include the diagonal vertical site energies, *ε*_*s*_, for all *N*_*e*_ states and off-diagonal electronic couplings, Γ_*us*_, between two neighboring EX states, between EX and CT states, and two CT states that share at least one charged site.^115^

The key feature of the EF model is its treatment of the system-bath coupling, which contains the local and nonlocal contributions, ${Ĥ}_{\mathrm{bs}}={Ĥ}_{\mathrm{bs}}^{L}+{Ĥ}_{\mathrm{bs}}^{\mathrm{NL}}$ . While an EX state couples only to its own local bath modes (local coupling), a CT state involving sites *a* and *b*, |CT_*a,b*_⟩, is coupled to the local bath modes of both sites simultaneously (nonlocal coupling). The local vibronic couplings are given by

111 $${Ĥ}_{\mathrm{bs}}^{L}=\overset{M}{\underset{a=1}{\sum }}c_{a}\cdot {\mathrm{R̂}}_{a}\left|{\mathrm{EX}}_{a}\right\rangle \left\langle {\mathrm{EX}}_{a}\right|$$

the same as the traditional Frenkel (IBH) model, whereas the nonlocal vibronic couplings are

112 $${Ĥ}_{\mathrm{bs}}^{\mathrm{NL}}=\overset{M}{\underset{a=1}{\sum }}\overset{M}{\underset{b\neq a}{\sum }}\left(c_{a}^{\left(+\right)}\cdot R_{a}+c_{b}^{\left(-\right)}\cdot R_{b}\right)\left|{\mathrm{CT}}_{a,b}\right\rangle \left\langle {\mathrm{CT}}_{a,b}\right|$$

Here, the vibronic coupling coefficients between the local EX state and the corresponding ground state **c**_*a*_ = (*c*_*a,1*_,···, *c*_*a,N*_) is the same as traditional Frenkel exciton model as in eq 19, whereas the vibronic coupling coefficients between the positively (negatively) charged site and the corresponding neutral site are $c_{a}^{\left(\pm \right)}=\left(c_{a,1}^{\left(\pm \right)},...,c_{a,N}^{\left(\pm \right)}\right)$ , respectively. In practice, only the CT states that are charged in the nearest neighbor sites are considered {|CT_*a,b*_⟩; *a* = *b* ± 1}, so there are many vanishing elements in the EF Hamiltonian in eq 110. Denote the *N*_*n*_-dimensional vibronic coupling vector for EX and CT states as

113 $$C_{{\mathrm{EX}}_{a}}=\left(0^{\left(a-1\right)},c_{a},0^{\left(M-a\right)}\right)$$

114 $$C_{{\mathrm{CT}}_{a,b}}=\left(0^{\left(a-1\right)},c_{a}^{\left(+\right)},c_{b}^{\left(-\right)},0^{\left(M-a-1\right)}\right)$$

respectively, such that the system-bath coupling Hamiltonian can be written using state-specific vibronic coupling vectors **C**_*s*_ (*s* = 1,···, *N*_*e*_):

115 $${Ĥ}_{\mathrm{bs}}=\overset{N_{e}}{\underset{s=1}{\sum }}C_{s}\cdot \mathrm{R̂}\left|s\right\rangle \left\langle s\right|$$

Burghardt and coworkers argued that the correlated spectral densities due to the nonlocal part of the vibronic coupling should be expressed as^115,319^

116 $$J_{\mathrm{EF}}^{\left(ss^{\prime }\right)}\left(\omega \right)=\frac{\pi }{2}\overset{N_{n}}{\underset{j=1}{\sum }}\frac{C_{s,j}C_{s^{\prime },j}}{\omega_{j}}\delta \left(\omega -\omega_{j}\right)$$

which is given in the mass-weighted coordinates, while the original expression was given in mass and frequency weighted coordinates such that ${Ĥ}_{\mathrm{bath}}=\frac{1}{2}\overset{N_{n}}{\underset{j=1}{\sum }}\omega_{j}\left({\mathrm{P̂}}_{j}^{2}+{\mathrm{R̂}}_{j}^{2}\right)$ and $J_{\mathrm{EF}}^{\left(ss^{\prime }\right)}\left(\omega \right)=\frac{\pi }{2}\overset{N_{n}}{\underset{j=1}{\sum }}C_{s,j}C_{s^{\prime },j}\delta \left(\omega -\omega_{j}\right)$ that is equivalent to eq 116. It is straightforward to show that the cross spectral density vanishes between two EX states since the vibronic coupling vectors are orthogonal, and the cross spectral densities between the two states that share at least one subspace of vibronic coupling, such as |EX_*a*_⟩ and |CT_*a,b*_⟩, have nonvanishing values.

The distinctions between the EF and MSH models are fundamental and can be summarized by four key points. First, their representations of the nuclear bath are conceptually different. The EF model employs *M* sets of independent, local normal modes, associated with each chromophore **R̂**_*a*_ (*a* = 1,···,*M*) and the total number of nuclear DOF is *N*_*n*_ = *MN*. The local modes of a single chromophore are typically obtained with electronic structure calculations of ground-state optimized molecular geometry, and thus the intermolecular modes for the environment effect might be absent. In contrast, the nuclear DOF of *F*-state MSH model represent a globally shared bath, and even the *N* effective bath modes in a specific subspace **R̂**_*a*_ (*a* = 1,···,*F* – 1) represent the global normal modes of the entire multichromophoric system including the environment and the total number of nuclear DOF is *N*_*n*_ = (*F* – 1)*N*. These global modes of the entire system and environment are often obtained with discretizing a transition spectral density between a pair of states (e.g., using eq 6) that is determined by the energy-gap TCF of these two states from the all-atom simulations of all chromophores and global environment (e.g., using eq 31). Suppose we want to treat the molecular normal modes in the MSH model, for each nuclear subspace, the number of global normal modes, *N*, should be equal to the sum of all molecular normal modes, that is *N* = *Mn*_*c*_ with *n*_*c*_ being the number of single molecular modes.

Second, the electronic bases have different typical scopes. In the tight-binding EF model of *M* chromophores, the basis is often limited to the single-exciton manifold, resulting in *M* ^2^ electronic states (including *M* local EX states and up to *M*(*M* – 1) CT states). In contrast, the MSH model is more general, capable of describing any arbitrary set of *F* diabatic states. These states can be local or delocalized, represent excitonic or charge-transfer character, or even include higher-lying excited states of a single complex molecule (such as CT1 and CT2 states of CPC_60_ triad), so long as the nuclear DOF are extended according to *N*_*n*_ = (*F* – 1)*N* and the parameters are determined from all-atom simulations. Of course, the EF model could be extended to include higher-lying excited states, resulting in a total of *F* states. However, the fundamental difference in construction would persist: the EF model would define *F* system-bath couplings relative to a central bath, whereas the MSH model is constructed from the *F*(*F* – 1)/2 pairwise relationships. In principle, for a simple donor–acceptor (D-A) dimer system, electronic structure calculation should be performed for the D–A dimer together, instead of two separate donor and acceptor systems for $c_{a}^{\left(+\right)}$ and $c_{b}^{\left(-\right)}$ couplings, respectively, as in the EF model.

Third, the philosophies for defining the system-bath couplings diverge. The EF model uses a “centralized” approach, where the vibronic couplings for all excited states (**c**_*a*_, $c_{a}^{\left(+\right)}$ , and $c_{b}^{\left(-\right)}$ for Frenkel exciton and positively and negatively charged states, respectively) are defined relative to a common reference ground-state bath *Ĥ*_bath_, where the ground state is not treated as an explicit electronic state |*g*⟩. This framework ensures consistency for the reorganization energies between each excited state and the ground state, but does not guarantee it for transitions between two different excited states. The MSH model, conversely, employs a “decentralized”, state-pairwise approach. It is constructed to rigorously satisfy all *F*(*F* – 1)/2 pairwise reorganization energies simultaneously, making no assumption about a central reference bath and thus providing a more complete and systematically derivable description of the electronic-vibrational coupling landscape.

Fourth, the mechanisms for generating bath correlations are different. The EF model induces correlations by including the CT states that couple to two independent local baths. In the MSH model, correlation is an intrinsic feature of the globally shared bath itself, mathematically encoded in the geometry of the PES minima within the extended (*F* – 1)-dimensional nuclear space. While both models represent significant advances over the uncorrelated IBH framework, the MSH model offers a more general and systematically derivable approach that is directly parametrized from the dynamics of the entire condensed-phase system. It is noted that the cross-spectral density $J_{\mathrm{EF}}^{\left(XY\right)}\left(\omega \right)$ in eq 116 does not correspond to the transition spectral density *J* ^*(XY)*^(ω) in eq 31 obtained from all-atom simulations for the underlying orthogonal local bath modes assumption in the EF model.

Next, we compare different combination schemes for determining the spectral density for a transition between two excited states, |*a*⟩ and |*b*⟩, given the spectral densities of their individual transitions from a common reference state, |*c*⟩. The MSH framework provides a clear prescription, which we term the global bath (GB) scheme. Based on the geometric construction of the MSH model and the observation of the similarity between the transition spectral densities, we can write the transition spectral density as

JGB(ab)(ω)=J(ab)(ω)=J(ac)(ω)+J(bc)(ω)−2J(ac)(ω)J(bc)(ω)cos⁡θab 117

where the angle θ_*ab*_ is determined by all-atom reorganization energies $E_{r}^{\left(ab\right)},E_{r}^{\left(ac\right)}$ , and $E_{r}^{\left(bc\right)}$ via the law of cosines, as shown in eq 40. This equation can be contrasted with two other common approximations. One is the local bath (LB) scheme, characteristic of the Frenkel exciton or IBH model, which assumes the baths are independent and their effects are simply additive:

118 $$J_{\mathrm{LB}}^{\left(ab\right)}\left(\omega \right)=J^{\left(ac\right)}\left(\omega \right)+J^{\left(bc\right)}\left(\omega \right)$$

This is a special case of the GB scheme where the correlation angle θ_*ab*_ is fixed at 90°. A third scheme is referred to as the nonlocal bath (NB) scheme:

119 $$J_{\mathrm{NB}}^{\left(ab\right)}\left(\omega \right)=\sqrt{J^{\left(ac\right)}\left(\omega \right)J^{\left(bc\right)}\left(\omega \right)}$$

which is motivated by the EF model and the following cross-spectral density definition

120 $$J^{\left(ac\right)}\left(\omega \right)=\frac{\pi }{2}\overset{N}{\underset{j=1}{\sum }}\frac{c_{ac,j}^{2}}{\omega_{j}}\delta \left(\omega -\omega_{j}\right)$$

121 $$J^{\left(bc\right)}\left(\omega \right)=\frac{\pi }{2}\overset{N}{\underset{j=1}{\sum }}\frac{c_{bc,j}^{2}}{\omega_{j}}\delta \left(\omega -\omega_{j}\right)$$

122 $$J_{\mathrm{cross}}^{\left(ab\right)}\left(\omega \right)=\frac{\pi }{2}\overset{N}{\underset{j=1}{\sum }}\frac{c_{ac,j}c_{bc,j}}{\omega_{j}}\delta \left(\omega -\omega_{j}\right)$$

Note that the cross spectral density $J_{\mathrm{cross}}^{\left(ab\right)}\left(\omega \right)$ ^41,320^ is different from the correlated spectral density of EF model in eq 116, since eq 122 measures the two different vibronic coupling coefficient sets within the same nuclear subspace of *N* modes, whereas eq 116 might involve multiple nuclear subspaces of up to *N*_*n*_ = *MN* modes.

We tested these three schemes by predicting the spectral density for the CT1 ↔ CT2 transition in the CPC_60_ triad, using the spectral densities for the ππ* ↔ CT1 and ππ* ↔ CT2 transitions as input. The results, shown in Figure 21, clearly demonstrate the superiority of the MSH-derived approach. The GB scheme almost perfectly reproduces the reference spectral density obtained directly from all-atom simulations. The NB scheme performs better than the LB scheme, but both deviate significantly, with the LB scheme drastically overestimating the spectral density’s amplitude. This discrepancy is quantified by the accumulated error in the reorganization energy, Δ*E*_*r*_(ω_*u*_), calculated using

123 $$\Delta E_{r}\left(\omega_{u}\right)=\frac{1}{\pi }\int_{0}^{\omega_{u}}\frac{J_{\mathrm{pred}}\left(\omega \right)-J_{\mathrm{ref}}\left(\omega \right)}{\omega }d\omega$$

The LB scheme results in a substantial error of 0.23 eV, whereas the GB scheme’s error is a negligible 0.01 eV. The failure of the simpler schemes is rooted in the geometric principles of the MSH model. Knowing the lengths of two sides of the PES triangle, $\sqrt{E_{r}^{\left(ac\right)}}$ and $\sqrt{E_{r}^{\left(bc\right)}}$ or equivalently {*c*_*ac,j*_} and {*c*_*bc,j*_}, is insufficient to determine the third side $\sqrt{E_{r}^{\left(ab\right)}}$ or $\left\{c_{ab,j}\right\}$ without knowing the angle between them. As shown in Figure 21d, the LB scheme implicitly forces this angle to be 90°, assuming the two CT states are orthogonal with respect to ππ* state. This incorrect geometric assumption elongates the distance between the CT1 and CT2 PES minima, leading to a significant overestimation of the reorganization energy and, consequently, an inaccurate spectral density. Additionally, the NB scheme is better than the LB scheme but still the resulting reorganization energy is overestimated compared with AA result, which can be seen in both the amplitude of spectral density and the accumulated error of reorganization energy. The accuracy of NB scheme is strongly affected by the input reorganization energies, but NB is not necessarily to be better than the LB scheme in the general case. Thus, the cross spectral density $J_{\mathrm{cross}}^{\left(ab\right)}\left(\omega \right)$ in eq 122 alone cannot be used to determine the transition spectral density *J* ^*(ab)*^(ω) that is consistent with all-atom simulation.

**Figure 21:**
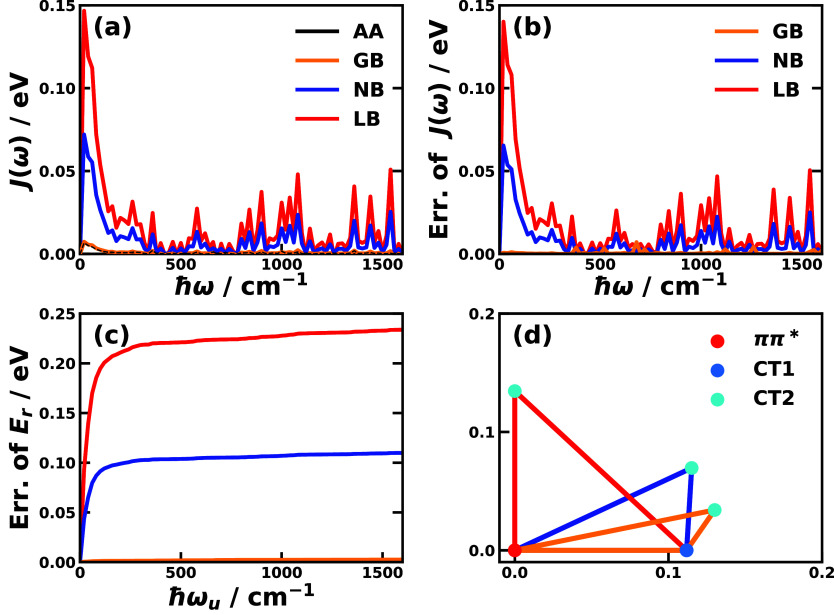
Spectral density *J* ^(CT1,CT2)^(ω) between CT1 and CT2 states of the CPC_60_ triad conformation 3 obtained from the knowledge of all-atom *J* ^(ππ^*^,CT1)^(ω) and *J* ^(ππ^*^,CT2)^(ω) using different bath combination schemes: global bath (GB) as in MSH model, nonlocal bath (NB), local bath (LB) as in Frenkel exciton model or IBH model, compared with the spectral density directly obtained from all-atom (AA) simulation (a). The error of spectral density with respect to the AA reference (b). The accumulative error for reorganization energy for different treatments as compared with the AA result (c). The PES minima triangles in GB (orange), NB (blue), and LB (red) schemes (d).

Similar to the MRC motivation to select out the most important modes, the effective-mode (EM) approach can be used to isolate the so-called effective modes that directly couple to the electronic DOF, and also couple to the rest of the nuclear modes.^321–326^ Starting with the EF model of an *M*-chromophore system, one could define *N*_*e*_ = *M* + *N*_*CT*_ effective modes, which is the sum of local excited states associated with each chromophore and *N*_CT_ (e.g., 2*M* – 2) CT states associated with two neighbor sites. The state-specific vibronic coupling in eq 115 can be written as ${\mathrm{Q̂}}_{s}=\frac{1}{{\mathrm{c̅}}_{s}}C_{s}\cdot \mathrm{R̂}$ with *c̅*_*s*_ the normalization constant, such that the system-bath coupling term is ${Ĥ}_{\mathrm{bs}}=\overset{N_{e}}{\underset{s=1}{\sum }}{\mathrm{c̅}}_{s}{\mathrm{Q̂}}_{s}\left|s\right\rangle \left\langle s\right|$ . The effective modes correspond to the first *N*_*e*_ rows of an orthogonal transformation matrix **T** as in the transformation **Q̂** = **TR̂**. Since the first *M* rows are chosen to represent the independent local baths of the EX states, thus they are orthogonal by design, the orthogonalization procedure will be applied to the *M* + 1 until the end of nuclear modes. The resulting *MN* orthogonal modes are thus called “global normal modes”. It is noted that these global normal modes are not equivalent to the global shared bath modes in MSH, since the former is the combination of local modes of individual chromophores and the latter is discretized from the energy-gap TCF of the entire system. The residual modes can form secondary layers that allow truncation to achieve dimensionality reduction, and the Mori chain representation was proposed for numerical implementation.^115^ The above EM model inherits the properties of the EF model, and they are equivalent if no truncation is applied. Another relevant non-Condon EM model by Cederbaum et al. is designed to capture the essential short-time dynamics through a conical intersection by representing the linear vibronic coupling (LVC) model with non-Condon electronic coupling.^327–332^ A key insight of this work is that through an orthogonal transformation of the bath coordinates, the complex, high-dimensional bath can be decomposed into *N*_*e*_(*N*_*e*_ + 1)/2 effective modes that couple directly to the *N*_*e*_ electronic DOF, and a set of residual modes that couple only to the effective modes. In two-state case shown in ref 327, the three effective modes are found to capture the short-time dynamics, while the residual bath becomes important at longer times. This construction provides a powerful tool for creating hierarchical reduced-dimensionality models with more complicated modes construction than the MRC model.

Another important framework for describing multistate systems is the vector system-bath (VSB) model proposed by Makri in 2023,^116^ which addresses the challenge of representing systems with local baths, such as molecular aggregates, within a traditional system-bath Hamiltonian form. The model was motivated by the notion that in multichromophoric systems composed of identical units, where the reorganization energy accompanying excitation transfer is independent of site-to-site distance, a feature that a simple common bath model cannot capture. The proposed solution is to replace the scalar system operator in the system-bath coupling with a vector coefficient. For the system of *M* chromophores, the VSB model shares the same nuclear and electronic bases as the SD-IBH model in eq 16. Suppose there are *N* local modes associated with each chromophore, the total nuclear DOF is *N*_*n*_ = *MN*. The VSB Hamiltonian expressed in the mass-weighted coordinates {*R̂*_*a,j*_|*a* = 1,···, *M*; *j* = 1,···, *N*} is given by

124 Ĥ=Ĥsys+Ĥbath+ĤbsĤsys=∑a=1Mεa|a⟩⟨a|+∑a≠bMΓab|a⟩⟨b|Ĥbath=∑a=1M∑j=1NP̂a,j22+12ωa,j2R̂a,j2

which are the same as the SD-IBH Hamiltonian, apart from a constant shift in site energies. The system-bath coupling term, however, is written as

125 $${Ĥ}_{\mathrm{bs}}=-\overset{N}{\underset{j=1}{\sum }}{\mathrm{R̂}}_{\left(j\right)}^{T}C_{\left(j\right)}Ŝ$$

Here, **R̂**_(*j*)_ = (*R̂*_*1,j*_,···, *R̂*_*M,j*_)^*T*^ is the vector of the *j*-th nuclear modes from all *M* local baths, **C**_(*j*)_ = diag­{*c*_*1,j*_,···, *c*_*M,j*_} is a diagonal matrix of coupling coefficients, and the system coordinate operator vector is defined by a set of *M*-component vectors {**σ**_*a*_}:

126 $$Ŝ=\overset{M}{\underset{a=1}{\sum }}\sigma_{a}\left|a\right\rangle \left\langle a\right|$$

with the *M*-component vectors

127 $$\sigma_{1}=\left(\begin{array}{l} \sigma_{1}^{e}\\ \sigma_{2}^{g}\\ \vdots \\ \sigma_{M}^{g} \end{array}\right),\sigma_{2}=\left(\begin{array}{l} \sigma_{1}^{g}\\ \sigma_{2}^{e}\\ \vdots \\ \sigma_{M}^{g} \end{array}\right),...,\sigma_{M}=\left(\begin{array}{l} \sigma_{1}^{g}\\ \sigma_{2}^{g}\\ \vdots \\ \sigma_{M}^{e} \end{array}\right)$$

These vectors determine the equilibrium shifts of the ground $\left(\sigma_{a}^{g}\right)$ and excited $\left(\sigma_{a}^{e}\right)$ PESs for each local site *a*. For identical monomers, the vectors are chosen such that the distances between any pair of PES minima are equal, which geometrically corresponds to placing the minima at the vertices of a regular polyhedron in an *M*-dimensional space.

The nuclear Hamiltonian of the *a*-th singly excited electronic state |*a*⟩ is then

128 $${Ĥ}_{a}=\varepsilon_{a}+{ĥ}_{a}^{e}+\overset{M}{\underset{b\neq a}{\sum }}{ĥ}_{b}^{g}$$

where the excited and ground state Hamiltonians for chromophoric site *a* is

129 $${ĥ}_{a}^{e/g}=\overset{N}{\underset{j=1}{\sum }}\frac{{\mathrm{P̂}}_{a,j}^{2}}{2}+\frac{1}{2}\omega_{a,j}^{2}{\left({\mathrm{R̂}}_{a,j}-\frac{c_{a,j}\sigma_{a}^{e/g}}{\omega_{a,j}^{2}}\right)}^{2}$$

that implies the equilibrium shifts of PES $R_{a,j}^{eq}=c_{a,j}\sigma_{a}^{e/g}/\omega_{a,j}^{2}$ from the reference bath Hamiltonian *Ĥ*_bath_. Geometrically, the distances between any pair of electronic states in the VSB model are equal, which means the PES minima are at the vertices of a regular polyhedron in *M*-dimensional space. For example, *M* = 3 corresponds to an equilateral triangle and *M* = 4 a regular tetrahedron.

The VSB framework is flexible. By choosing the structure of the system vectors {**σ**_*a*_}, one can describe various physical scenarios. For example, setting $\sigma_{a}^{g}=0$ and $\sigma_{a}^{e}=1$ corresponds to choosing the reference bath as the ground state of all sites, and thus reduces the model to the standard SD-IBH case with independent local baths. If the coefficient vector in the system operator couples two or more local bath subspaces, the correlated bath like the EF model would emerge, e.g., **σ**_1_ = κ_1_(1,0,0,···, 0)^*T*^ and $\sigma_{2}=\kappa_{2}{\left(1/\sqrt{2},1/\sqrt{2},0,...,0\right)}^{T}$ . If all coefficient vectors point along the same direction such that the PES minima are collinear, the VSB model reduces to the common bath model.

The MSH model can also be cast into this system-bath vector form, though its physical foundation is different. It is noted that the nuclear DOF of *N*_*n*_ = (*F* – 1)*N* and how the pairwise reorganization energies are obtained and transformed into the heterogeneous PES equilibrium shifts are fundamentally different from the VSB model. The MSH model from eq 23 can be rewritten as

130 $$Ĥ={Ĥ}_{\mathrm{sys}}+{Ĥ}_{\mathrm{bath}}+{Ĥ}_{\mathrm{bs}}$$

$${Ĥ}_{\mathrm{sys}}=\overset{F}{\underset{s=1}{\sum }}\left(\varepsilon_{s}+E_{r}^{\left(1s\right)}+\cdot \cdot \cdot +E_{r}^{\left(s-1,s\right)}\right)\left|s\right\rangle \left\langle s\right|+\overset{F}{\underset{u\neq s}{\sum }}\Gamma_{su}\left|s\right\rangle \left\langle u\right|$$ 131

132 $${Ĥ}_{\mathrm{bath}}=\overset{F-1}{\underset{a=1}{\sum }}\overset{N}{\underset{j=1}{\sum }}\frac{{\mathrm{P̂}}_{a,j}^{2}}{2}+\frac{1}{2}\omega_{j}^{2}{\mathrm{R̂}}_{a,j}^{2}$$

133 $${Ĥ}_{\mathrm{bs}}=-\overset{N}{\underset{j=1}{\sum }}C_{j}{\mathrm{R̂}}_{\left(j\right)}^{T}Ŝ$$

where **R̂**_(*j*)_ = (*R̂*_1,*j*_,*R̂*_2,*j*_,···, *R̂*_*F*−1,*j*_)^*T*^ denotes the nuclear coordinates of the *j*-th physical mode in all (*F* – 1) subspaces, the coefficient for the *j*-th mode $C_{j}=\omega_{j}\sqrt{2/N}\left(j=1,...,N\right)$ , and the coefficient vectors in the system operator **Ŝ** are given by **σ**_1_= **0** and

134 σ2=Er(12)(100⋮0),σ3=Er(13)(cos⁡θ23sin⁡θ230⋮0),σ4=Er(14)(cos⁡θ24sin⁡θ24cos⁡θ34′sin⁡θ24sin⁡θ34′⋮0),···

While both the MSH and VSB models utilize an extended-dimensional nuclear space and a geometric picture of PES minima, their underlying philosophies and primary applications are distinct. First, the nuclear bath bases are different. The VSB model is built upon the local bath modes associated with individual chromophoric sites, similar to the Frenkel and EF models, whereas the MSH model uses the global, collective modes derived from all-atom TCFs of the entire condensed-phase system. Consequently, the MSH spectral densities are inherently rich in the low-frequency intermolecular modes that drive environmental relaxation. Second, the treatment of the environment’s heterogeneity is fundamentally different. The VSB model is principally designed for symmetric systems, such as aggregates of identical monomers, where the assumption of equal pairwise reorganization energies leads to a regular polyhedron geometry. In contrast, the MSH model is a data-driven framework for general heterogeneous environments, where the typically unequal reorganization energies from all-atom simulations result in an irregular polyhedron geometry that quantitatively encodes the realistic system’s specific correlations. Third, the system-bath coupling vector formulation can be applied to both VSB and MSH models, and they differ in the coefficient vectors in the system operator. The VSB formulation might be more straightforward to apply in some theoretical methods, such as the iterative quasi-adiabatic propagator path integral (QuAPI)^333,334^ and the small matrix path integral (SMatPI),^335,336^ whereas the MSH model provides a general description, which is particularly useful to operate in the diagonal and off-diagonal parts of the overall Hamiltonian rather than the system-bath form, such as in the partial linearized density matrix (PLDM) algorithm,^107,108^ whose dynamical result may depend on how to partition the state-dependent and state-independent parts. Although VSB and MSH may look similar mathematically, their actual physical meanings differ; if limited to the same nuclear space, the VSB model could be viewed as a special case of the MSH model. Also, when the two spectral densities are truly uncorrelated, the MSH could reduce to the IBH or VSB case as discussed in the EET of Y6 dimer separated by different distances, as shown in Figure 8.

The preceding discussion has focused on the EF and VSB models as key points of comparison due to their sophisticated treatment of bath correlations. It is important to note that there are other notable frameworks for describing correlated environments, but they are different from the MSH/MRC approach. The EF and VSB models serve as particularly insightful frameworks because their underlying concepts, either through nonlocal couplings or an extended vector space, show a clear lineage of thinking toward solving the same fundamental problem that the MSH/MRC framework addresses. The comparison highlights the unique contribution of the MSH/MRC model. While other models provide a way to define correlated bath, often for specific classes of systems, the MSH framework distinguishes itself as a systematically derivable approach designed to build quantitatively predictive Hamiltonians for general, heterogeneous condensed-phase systems directly from all-atom simulation data.

### 6.2 Current Limitations and Future Extensions of the MSH/MRC Framework

While the MSH/MRC framework provides a robust and physically grounded approach for modeling nonadiabatic dynamics in the condensed phase, it is important to acknowledge its underlying assumptions and limitations, which also point toward exciting avenues for future development. The most significant of these is the harmonic approximation for the nuclear DOF. This approximation is justified for many condensed-phase systems where the collective influence of a large number of weak environmental interactions can be described by Gaussian statistics, as dictated by the central limit theorem. The remarkable agreement between MSH and full all-atom dynamics for systems like the CPC_60_ triad and MPe/TCNE trimer validates this approach for environments dominated by such collective solvent motions. However, this harmonic representation may be insufficient for systems where specific, large-amplitude anharmonic motions play a dominant role. For instance, low-frequency torsional modes in flexible molecules, such as retinal photoisomerization^16,337–340^ or specific protein conformational changes,^89–92^ are not well-described by simple harmonic oscillators. The anharmonicity may become more important in small molecules when the accurate full-dimensional PES is required to resolve the wavepacket dynamics.^339,341–343^ Neglecting this anharmonicity could lead to an inaccurate description of the reaction dynamics and the fluctuations of electronic energies, particularly for processes that are strongly coupled to such specific molecular motions.

Another key aspect of the current MSH/MRC parametrization is its reliance on classical MD simulations to generate the necessary energy-gap time correlation functions. While the resulting MSH model itself can be, and often is, treated with more accurate quantum or semiclassical dynamics, the parameters that define the model are derived from a classical description of the nuclear environment. This approach implicitly neglects any nuclear quantum effects, such as zero-point energy,^344–348^ isotope effect,^349–352^ and tunneling,^353–363^ that might be present in the underlying all-atom system. For systems dominated by high-frequency vibrations or involving light atoms like hydrogen, these effects can be significant.^344,349,350,353–356,361,363^ Furthermore, the standard MSH construction assumes that the vibrational frequencies are the same across all electronic states, which are based on transitions rather than individual states. While this is a reasonable choice for many systems, it neglects potential state-dependent changes in the vibrational structure, such as Duschinsky rotations,^284,364–372^ which could influence the dynamics, especially in cases of significant charge redistribution that alters molecular bonding patterns.

These limitations naturally suggest several promising directions for extending the MSH/MRC framework. A primary frontier is the development of anharmonic extensions. One potential strategy involves a hybrid approach, where the bulk of the environment is still treated as a harmonic bath, but a few specific, physically important anharmonic modes are treated explicitly. This would be particularly relevant for systems where a small number of well-defined intramolecular vibrations, such as key torsional modes, are known to be critical for the nonadiabatic process. Another avenue is to move beyond the linear response approximation used to derive the model parameters from the energy gap TCFs, potentially incorporating higher-order correlation functions that can capture non-Gaussian environmental statistics.

A second major extension is the incorporation of non-Condon effects,^115,286,299,319,341,373^ where the electronic couplings (Γ_*XY*_) are allowed to depend on the nuclear coordinates. In the current framework, these couplings appear as constant, off-diagonal terms. However, it is well-known that for many systems, these couplings are modulated by vibrational motion. The MSH framework could be extended to include such effects by parametrizing the electronic couplings as functions of the nuclear coordinates, for instance, through a linear vibronic coupling scheme where the couplings depend linearly on the MSH normal mode coordinates.^296,330,374^ This would require additional input from electronic structure calculations, where the couplings are computed across a range of nuclear geometries, but would enable the MSH model to describe a wider class of nonadiabatic phenomena, including electronic transition through conical intersections and long-range charge transfer. Finally, inspiration can be drawn from hierarchical effective-mode theories to develop a multilayered representation of the MSH bath,^115,327,330^ Such a hierarchical structure could provide a more systematic way to partition the environment, separating the fast, strongly coupled modes from the slower, dissipative modes, potentially leading to more efficient simulation strategies and a deeper understanding of the different time scales of environmental response.

## 7 Concluding Remarks

This Review has traced the development and application of the multistate harmonic (MSH) and multistate reaction coordinate (MRC) models, a unified framework for constructing effective Hamiltonians for nonadiabatic dynamics in complex condensed-phase systems. We began by highlighting the fundamental challenge posed by the “curse of dimensionality” and the limitations of foundational models like the spin-boson and Frenkel exciton Hamiltonians. These traditional approaches, while powerful, often fail to capture the heterogeneous and correlated nature of the nuclear environment, a critical aspect of realistic chemical and biological processes. The isolated bath assumption, in particular, proves insufficient for describing systems where multiple electronic states are coupled to a common, shared environment, such as in multichromophoric aggregates or single molecules with dense excited-state manifolds.

The MSH model provides a systematic and physically rigorous solution to this challenge. Its central innovation is a geometric construction in an extended nuclear space, which allows the *F*-state model to consistently incorporate the full matrix of *F*(*F* – 1)/2 pairwise reorganization energies derived from all-atom simulations. This approach ensures that the electronic-vibrational couplings for every possible transition are accurately represented, resulting in a model that faithfully reproduces the nonadiabatic dynamics of the underlying all-atom system, as demonstrated for both photoinduced charge transfer and excitation energy transfer processes. The exactly equivalent MRC representation further enhances the framework by providing a clear and intuitive physical picture, distilling the complex, high-dimensional bath dynamics into the motion along a few key reaction coordinates.

The versatility of the MSH/MRC framework extends beyond the simulation of specific realistic systems. Its well-defined and analytically tractable structure makes it an ideal platform for method development and rigorous benchmarking. We have shown its utility in systematically exploring the parameter space of nonadiabatic dynamics, providing a comprehensive map of dynamical behaviors across different physical regimes. Furthermore, by enabling direct comparison with numerically exact tensor-train methods, the MSH model serves as a crucial tool for validating the accuracy of widely used approximate dynamical methods, including various semiclassical approaches and perturbative quantum master equations. Combining with advanced machine-learning time-series techniques, the nonadiabatic dynamical simulation of MSH/MRC models would be more efficient.^375–378^ By bridging the gap between high-fidelity atomistic simulations and tractable theoretical models, the MSH/MRC framework offers a powerful and predictive toolkit for advancing our understanding of quantum dynamics in the complex environments that characterize modern chemistry, biology, and materials science.^379–388^

## Supplementary Material



## Appendix A

### Equilibrium Shifts of PESs in MSH Model

The minima of PESs in a general *F*-state MSH model are located at the origin and the following equilibrium shift components $\left\{S_{j}^{(aX)}\right\}$ in the *D* = *F* - 1 extended dimensions:

A.1
$$\left(\begin{array}{llllll} S_{j}^{(12)} & & \\ S_{j}^{(13)} & S_{j}^{(23)} & \\ S_{j}^{(14)} & S_{j}^{(24)} & S_{j}^{(34)}\\ S_{j}^{(15)} & S_{j}^{(25)} & S_{j}^{(35)} & S_{j}^{(45)}\\ \vdots & \vdots & \vdots & \vdots & \ddots \\ S_{j}^{(1D)} & S_{j}^{(2D)} & S_{j}^{(3D)} & S_{j}^{(4D)} & \cdots & S_{j}^{(D-1,D)} \end{array}\right)=\sqrt{\frac{2}{N}}\frac{1}{\omega_{j}}\left(\begin{array}{llllll} A_{1}^{(12)} & & \\ A_{1}^{(13)} & A_{2}^{(13)} & \\ A_{1}^{(14)} & A_{2}^{(14)} & A_{3}^{(14)}\\ A_{1}^{(15)} & A_{2}^{(15)} & A_{3}^{(15)} & A_{4}^{(15)}\\ \vdots & \vdots & \vdots & \vdots & \ddots \\ A_{1}^{(1D)} & A_{2}^{(1D)} & A_{3}^{(1D)} & A_{4}^{(1D)} & \cdots & A_{D-1}^{(1D)} \end{array}\right)$$

where the positions of these vertices $\left\{A_{a}^{(1X)}\right\}$ are given by

A(12)=Er(12)(1,0,...,0)A(13)=Er(13)(cos⁡θ23,sin⁡θ23,0,...,0)A(14)=Er(14)(cos⁡θ24,sin⁡θ24cos⁡θ34′,sin⁡θ24sin⁡θ34′,0,...,0)A(15)=Er(15)(cos⁡θ25,sin⁡θ25cos⁡θ35′,sin⁡θ25sin⁡θ35′cos⁡θ45′,sin⁡θ25sin⁡θ35′sin⁡θ45′,0,...,0)A(16)=Er(16)(cos⁡θ26,sin⁡θ26cos⁡θ36′,sin⁡θ26sin⁡θ36′cos⁡θ46′,sin⁡θ26sin⁡θ36′sin⁡θ46′cos⁡θ56′,sin⁡θ26sin⁡θ36′sin⁡θ46′sin⁡θ56′,0,...,0)···A(1D)=Er(1D)(cos⁡θ2D,sin⁡θ2Dcos⁡θ3D′,sin⁡θ2Dsin⁡θ3D′cos⁡θ4D′,sin⁡θ2Dsin⁡θ3D′sin⁡θ4D′cos⁡θ5D′,sin⁡θ2Dsin⁡θ3D′sin⁡θ4D′sin⁡θ5D′cos⁡θ6D′,...,sin⁡θ2Dsin⁡θ3D′sin⁡θ4D′sin⁡θ5D′···sin⁡θD−2,D′cos⁡θD−1,D′,sin⁡θ2Dsin⁡θ3D′sin⁡θ4D′sin⁡θ5D′···sin⁡θD−2,D′sin⁡θD−1,D′)
A.2

Here, cos*θ*_*ik*_ is defined in eq 35 and cosines of $\theta_{ik}^{\prime }$ are given by

A.3
$$\cos\theta_{3k}^{\prime }=\frac{\cos\theta_{3k}-\cos\theta_{23}\cos\theta_{2k}}{\sin\theta_{23}\sin\theta_{2k}},\left(k\geq 4\right)$$

A.4
cos⁡θ4k′=cos⁡θ4k−cos⁡θ24cos⁡θ2k−sin⁡θ24sin⁡θ2kcos⁡θ34′cos⁡θ3k′sin⁡θ24sin⁡θ2ksin⁡θ34′sin⁡θ3k′(k≥5)

A.5
cos⁡θ5k′=[cos⁡θ5k−cos⁡θ25cos⁡θ2k−sin⁡θ25sin⁡θ2k(cos⁡θ35′cos⁡θ3k′+sin⁡θ35′sin⁡θ3k′cos⁡θ45′cos⁡θ4k′)]×[sin⁡θ25sin⁡θ2ksin⁡θ35′sin⁡θ3k′sin⁡θ45′sin⁡θ4k′]−1,(k≥6)

cos⁡θik′=[cos⁡θik−cos⁡θ2icos⁡θ2k−sin⁡θ2isin⁡θ2kcos⁡θ3i′cos⁡θ3k′−···−sin⁡θ2isin⁡θ2ksin⁡θ3i′sin⁡θ3k′sin⁡θ4i′sin⁡θ4k′···sin⁡θ(i−2)i′sin⁡θ(i−2)k′cos⁡θ(i−1)i′cos⁡θ(i−1)k′]×[sin⁡θ2isin⁡θ2ksin⁡θ3i′sin⁡θ3k′sin⁡θ4i′sin⁡θ4k′⋯sin⁡θ(i−2)i′sin⁡θ(i−2)k′sin⁡θ(i−1)i′sin⁡θ(i−1)k′]−1(4≤i<k)
A.6

## Notes

List of abbreviation, separate comparison of MSH and all-atom nonadiabatic dynamics, simulation details of MPe/2TCNE trimer in acetonitrile, Y6 dimer in chloroform, and the MSH models in different parameter regions.(PDF)
