# Supplementary material for: Consistent and Generalizable Effective Model Hamiltonian Framework for Studying Nonadiabatic Dynamics in the Condensed Phase
Source: J Chem Theory Comput. 2025 Dec 9;21(24):12393–442. doi: 10.1021/acs.jctc.5c01586 (PMC12746476; doi:10.1021/acs.jctc.5c01586)
Supplement: Supplementary file 1 [file ct5c01586_si_001.pdf]

Supporting Information: Consistent and Generalizable Effective Model Hamiltonian Framework for Studying Nonadiabatic Dynamics in the Condensed Phase

Zengkui Liu,[1,2,3] Hao Zeng,[1,2,4] and Xiang Sun[1,2,4,3,a)]
[1)] *Division of Arts and Sciences, NYU Shanghai, 567 West Yangsi Road, Shanghai 200124, China*
[2)] *NYU-ECNU Center for Computational Chemistry at NYU Shanghai, 3663 Zhongshan Road North, Shanghai 200062, China*
[3)] *Department of Chemistry, New York University, New York, New York 10003, United States*
[4)] *State Key Laboratory of Precision Spectroscopy, East China Normal University, Shanghai 200062, China*

I. LIST OF ABBREVIATIONS

Abbreviation	*Meaning*
AA	all-atom
ACN	acetonitrile
BChl	bacteriochlorophyll
CL	Caldeira-Leggett
CMM	classical mapping model
CPC_{60}	carotenoid-porphyrin-fullerene
CT	charge-transfer
D-A	donor-acceptor
DOF	degrees of freedom
EET	excitation energy transfer
EF	extended Frenkel
EM	effective-mode
EX	excitonic
FCD	fragment charge difference
FED	fragment energy difference
FGR	Fermi's golden rule
FMO	Fenna-Matthews-Olson
FSSH	fewest-switches surface hopping
GB	global bath
GMH	generalized Mulliken-Hush
GOA	Garg-Onuchic-Ambegaokar
GQME	generalized quantum master equation
IBH	isolated bath harmonic
IMT	instantaneous Marcus theory
LB	local bath
LSC	linearized semiclassical
LVC	linear vibronic coupling
MD	molecular dynamics
MF	mean-field
MMST	Meyer-Miller-Stock-Thoss
MPe	methylperylene
MPS	matrix product state
MRC	multistate reaction coordinate
MSH	multistate harmonic
NB	nonlocal bath
NE-FGR	nonequilibrium Fermi's golden rule
NFA	non-fullerene acceptor
NQE	nuclear quantum effects

[a)] Electronic mail: xiang.sun@nyu.edu

NZ-GQME	Nakajima-Zwanzig generalized quantum master equation
PES	potential energy surface
PICT	photoinduced charge transfer
PLDM	partial linearized density matrix
QM/MM	quantum mechanics/molecular mechanics
QME	quantum master equation
QuAPI	quasi-adiabatic propagator path integral
RC	reaction coordinates
RDM	reduced density matrix
RI-LSC	resolution-of-identity linearized semiclassical
SD-IBH	state-dependent isolated bath harmonic
SI-IBH	state-independent isolated bath harmonic
SMatPI	small matrix path integral
SPM	spin-mapping model
SQC	symmetrical quasiclassical
TC	time-convolution
TCF	time correlation function
TCL	time-convolutionless
TCNE	tetracyanoethylene
TFD	thermofield dynamics
THF	tetrahydrofuran
TT	tensor-train
TT-KSL	tensor-train KSL
TT-TFD	tensor-train thermofield dynamics
VSB	vector system-bath

II. COMPLIMENTARY COMPARISON BETWEEN MSH AND ALL-ATOM NONADIABATIC DYNAMICS

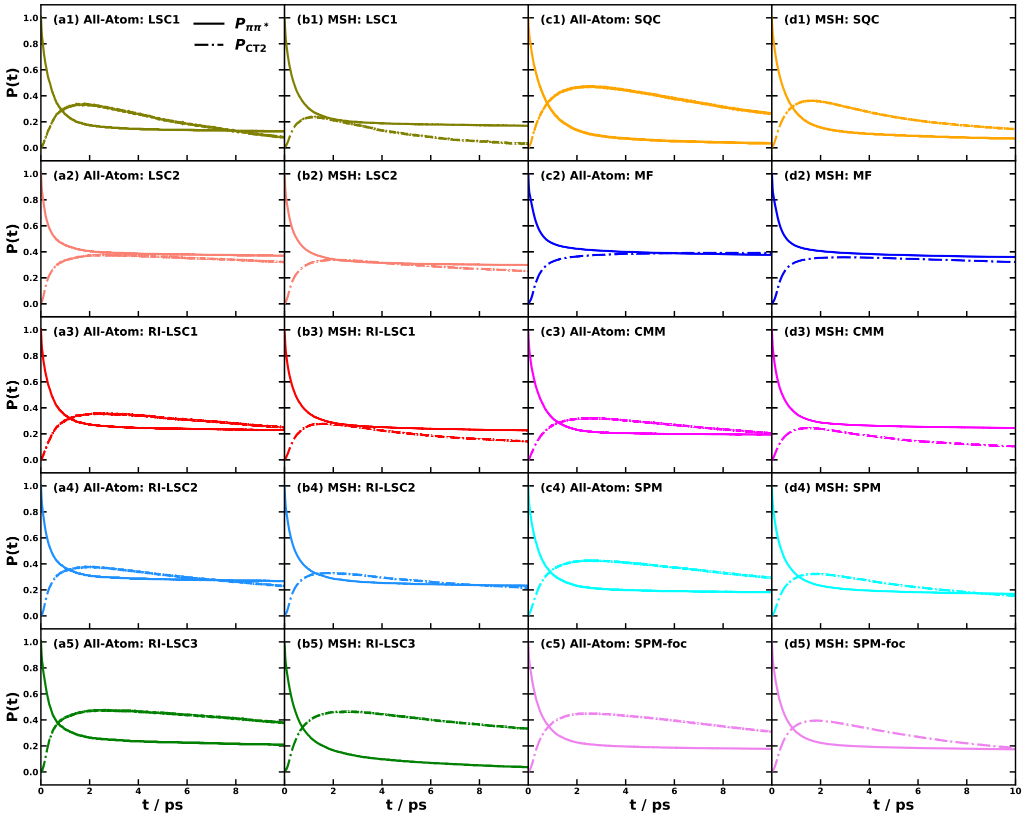

FIG. S1. Comparison of the population dynamics of $\pi\pi^*$ (solid line) and CT2 (dash-dot line) states for the CPC_{60} triad conformation 3 in THF at 300 K, simulated with various Hamiltonians and dynamical methods. The columns (a,c) are all-atom results, whereas the columns (b,d) are MSH results. This figure contains the same result as reported in Fig.4 in the main text and is adapted from Ref. 1.

III. SIMULATION DETAILS OF MPE/2TCNE TRIMER IN ACETONITRILE

A. Quantum Chemistry Calculation

The geometry of the trimer, consisting of one methylperylene (MPe) and two tetracyanoethylene (TCNE) molecules, was optimized at the PM6 level of theory using Gaussian16.[2,3] For the solvent molecule, acetonitrile (ACN), the optimized geometry and atomic partial charges were obtained at the CCSD/aug-cc-pVDZ level with Gaussian16. Following the geometry optimizations, excited-state properties were evaluated with Q-Chem6[4] at the PBE0/6-31+G(d,p) level under the Tamm–Dancoff approximation.[5,6] These calculations provided excitation energies, fragment charge difference diabatic couplings, and Merz–Kollman (MK) atomic partial charges.[5,7,8]

Four electronic states are of interest in this system: the ground state (GS), the brightest excited state (EX), and two distinct charge-transfer states (CT1 and CT2). The EX state is defined as the state with the largest oscillator strength among the first 25 singlet excited states. The CT1 state involves an electron transfer from the MPe donor to the first TCNE acceptor, while the CT2 state corresponds to an electron transfer from the MPe donor to the second TCNE acceptor.

B. All-Atom Molecular Dynamics Simulation and MSH Model Construction

The simulations are based on an all-atom multistate Hamiltonian constructed using the general AMBER force field (GAFF).[9] The model incorporates state-specific atomic partial charges obtained from the quantum chemistry calculations described above. The simulation system consists of a DAA trimer, formed by one MPe donor and two TCNE acceptors, dissolved in a box with 1440 acetonitrile (ACN) solvent molecules. The potential energy, V_X, for each state in this Hamiltonian are defined as:

$$V_X(\mathbf{R}) = V_X^{\mathrm{GAFF}}(\mathbf{R}) + W_X(\mathbf{r}). \tag{S1}$$

Here, X represents one of the four states of interest $\{\mathrm{GS},\ \mathrm{EX},\ \mathrm{CT1},\ \mathrm{CT2}\}$, $\mathbf{R}$ is the nuclear conformation of the entire system, and $\mathbf{r}$ is the nuclear conformation of the solute trimer. The state-specific energy correction is given by:

$$W_X(\mathbf{r}) = E_X(\mathbf{r}) - V_X^{\mathrm{GAFF}}(\mathbf{r}). \tag{S2}$$

All molecular dynamics (MD) simulations were propagated on the ground state potential energy surface (PES) using the QCDyn software, a modified version of OpenMM 7.5.0.[10] A nuclear time step of $\delta t = 1$ fs was used throughout the simulations. Electrostatic interactions were calculated using the Particle Mesh Ewald (PME) method,[11] and a unified non-bonding force cut-off radius of 9 Å was applied to both van der Waals interactions and the real-space part of the PME calculation. Regarding constraints, all covalent bonds, including those with hydrogen atoms, were constrained with the SHAKE algorithm,[12] while the solute molecules were additionally restrained with a harmonic force constant of 100 kcal/(mol Å^2). For temperature control in all relevant steps, a Langevin thermostat with a friction coefficient of 1 ps^{-1} was employed.

The preparation of the MSH model followed a sequential MD procedure performed with the EX force field:

1. Energy minimization: Performed until the tolerance reached 10 kJ/mol or for a maximum of 10^4 optimization cycles.

2. Heating: The system was heated from 0 K to 300 K over a period of 100 ps with Langevin thermostat.

3. NPT Equilibration: The system was equilibrated at a temperature of 300 K and a pressure of 1 bar. A Monte-Carlo barostat was applied every 25 steps. The averaged simulation box size was $60.9 \times 60.9 \times 60.9$ Å^3.

4. NVT Equilibration: A 2 ns equilibration was performed under NVT conditions.

5. NVT Sampling: Initial conditions were sampled from this trajectory, saving snapshots every 100 ps for subsequent NVE runs and every 1 ps for nonadiabatic molecular dynamics (NAMD) runs.

6. NVE Dynamics: Following a 50 ps NVE relaxation, the equilibrium NVE trajectories of 100 ps were used to obtain all the four states' potential energy trajectories.

7. The MSH bath parameters was determined from the pairwise energy gap time correlation functions, which were averaged over 10^7 snapshots.

C. Nonadiabatic Molecular Dynamics Simulation

The all-atom NAMD simulations included the same energy minimization, heating, NPT, and NVT relaxation steps as mentioned the MSH protocol. Initial phase space points were drawn from a canonical sampling in equilibrium with the ground state PES. For the dynamics, nuclear variables were propagated with a time interval of 0.5 fs using the velocity-Verlet algorithm, while electronic variables were propagated using the 8th-order Runge-Kutta (RK8) method. The final results are an average over 10^5 trajectories of 2 ps.

For the NAMD simulations of the MSH model for the MPe/2TCNE trimer in ACN was configured with $N = 100$ physical modes. Nuclear variables were evaluated every 1 fs using the velocity-Verlet algorithm. Within each nuclear step, the electronic variables were propagated 20 times using the 4th-order Runge-Kutta (RK4) method. The NAMD results for the MSH model are an average over 10^6 trajectories of 10 ps.

TABLE S2. MPe/2TCNE trimer properties including excited state indexes, excitation energies E_X (in eV), intermolecular charge transfer amount change upon excitation ΔQ_{DA_1} (in e), vertical correction energies W_X (in eV), and vertical shift energies (in eV) of the MSH model.

State X	Excited State index	E_X / eV	ΔQ_{DA_1} / e	W_X / eV	ε_X
EX	11	3.078	0.003	3.343	3.080
CT1	7	2.164	1.920	1.534	1.508
CT2	10	2.377	0.974	-0.464	0.903
GR	NA	0	0	0	0

TABLE S3. Diabatic couplings and reorganization energies between states of the MPe/2TCNE trimer. The diabatic coupling between any excited state and the ground state is set to 0.

Transition XY	Γ_{XY} / eV	$E_r^{(XY)}$ / eV
EX ↔ CT1	-0.074	0.823
EX ↔ CT2	0.020	1.648
CT1 ↔ CT2	-0.010	1.974
GR ↔ EX	0	0.006
GR ↔ CT1	0	0.829
GR ↔ CT2	0	1.629

IV. SIMULATION DETAILS OF Y6 DIMER IN CHLOROFORM

A. Quantum Chemistry Calculation

The system under study is a face-to-face Y6 dimer, composed of a Y6 monomer and its translational replica displaced along the X-axis. The quantum chemistry procedure for the Y6 monomer is identical to that described in Ref. 13.

The excited-state properties of the dimer were calculated using Time-Dependent Density Functional Theory (TDDFT). These calculations were performed at the ω^*B97X-D/6-31G(d,p) level of theory with an optimally tuned range separation parameter of $\omega = 0.11$. A polarizable continuum model (PCM) with a dielectric constant of $\varepsilon_0 = 4.8$ was used to represent the chloroform solvent environment. All TDDFT calculations were carried out with Q-Chem 6, yielding excitation energies, transition dipoles, and MK atomic partial charges.[4,14] The primary locally excited state, S_1, was identified as the brightest state, with an excitation energy of 1.72 eV, an oscillator strength of 2.54, and a transition dipole of $(-7.76,0,0)$ in atomic units.

TABLE S4. Y6 dimer properties including excited state indexes, excitation energies E_X (in eV), intramolecular charge transfer amount change due to excitation ΔQ (in e), vertical correction energies W_X (in eV), and vertical energies ε_X (in eV) in the MSH model.

State X	Excited State index	E_X / eV	ΔQ / e	W_X / eV
EX	1	1.720	0.308	-1.14
GR	–	0	0	0

TABLE S5. Diabatic couplings and reorganization energies $E_r^{(XY)}$ of the transitions between states of the Y6 dimer with different Y6 distances. The diabatic coupling between any excited state and the ground state is set to 0.

Distance / Å	Transition XY	Γ_{XY} / eV	$E_r^{(XY)}$ / eV
5	GR ↔ EX1	0	0.060
	GR ↔ EX2	0	0.060
	EX1 ↔ EX2	0.0505	0.062
10	GR ↔ EX1	0	0.073
	GR ↔ EX2	0	0.072
	EX1 ↔ EX2	0.0505	0.120
20	GR ↔ EX1	0	0.084
	GR ↔ EX2	0	0.084
	EX1 ↔ EX2	0.0505	0.165

B. All-Atom Molecular Dynamics Simulation and MSH Model Construction

The construction of the all-atom multistate Hamiltonian follows the same methodology as the reference system. The diabatic coupling between the locally excited states on the two Y6 monomers was evaluated as the classical interaction between their transition dipoles, assuming a separation distance of 10 Å, which is 50.5 meV. The simulation box contained one Y6 dimer solvated by 1880 chloroform (TCM) molecules. The general molecular dynamics parameters are identical to those used for the previously described DAA system, with the only exception being the NPT equilibrated box size, which is $10.2 \times 5.1 \times 5.1 \text{Å}^3$ at a pressure of 1 bar. The MSH model construction was the same as the above MPe/2TCNE trimer case. Furthermore, the same MD procedure was utilized for both the MSH model construction and for generating the canonical distribution of initial nuclear phase space points for NAMD.

C. Nonadiabatic Molecular Dynamics Simulation

All-Atom NAMD. The final results were averaged over 10^5 independent trajectories of 1 ps. Both nuclear and electronic variables were propagated with a time step of 1 fs, using the velocity-Verlet and 8th-order Runge-Kutta (RK8) algorithms, respectively.

The NAMD simulations of MSH and isolated bath harmonic (IBH) models were averaged over 10^6 trajectories of 10 ps. Nuclear phase space points were evaluated every 1 fs using the velocity-Verlet algorithm. Within each nuclear step, the electronic mapping variables were updated 20 times using the 4th-order Runge-Kutta (RK4) algorithm.

V. PARAMETERS OF MSH MODELS WITHIN DIFFERENT PARAMETER REGIONS

A. Model Parameters

We report the detailed parameters of MSH S1–S9 (a-e) in Fig. 11, MSH rotating shift vector (RSV) models with different angle θ_{23} in Fig. 12 and reduced three-state MSH model for Y6 dimer dissolved in chloroform at distance of 10 Å in Fig. 14 of the main text. All of these MSH models follow the three-state MSH Hamiltonian:

$$\begin{aligned}\hat{H} &= \sum_{i=1}^{3} \varepsilon_i |i\rangle\langle i| + \sum_{i>j}^{3} \Gamma_{ij} (|i\rangle\langle j| + |j\rangle\langle i|) \\ &+ \sum_{i=1}^{3}\sum_{a=1}^{2}\sum_{k=1}^{N} \left[\frac{\hat{P}_{a,k}^2}{2} + \frac{1}{2}\omega_k^2 \left(\hat{R}_{a,k} - S_k^{(ai)} \right)^2 \right] \otimes |i\rangle\langle i|.\end{aligned} \tag{S3}$$

Here, reorganization energy is evenly distributed to all normal modes $E_r^{(XY)} = \frac{N}{2}\sum_{a=1}^{2} \omega_k^2 (S_k^{(aX)} - S_k^{(aY)})^2$, and we can introduce PES shift matrix $A_b^{(ai)} = \sqrt{\frac{N}{2}}\omega_k S_k^{(ai)}$ such that $E_r^{(XY)} = \sum_{a=1}^{2} (A_b^{(aX)} - A_b^{(aY)})^2$.

Except for Y6 dimer MSH model that uses realistic spectral density from all-atom simulation, the vibrational normal modes of MSH models S1–S9 and RSV are defined by the Ohmic spectral density:

$$J(\omega) = \frac{\pi}{2}\hbar\xi\,\omega\, e^{-\omega/\omega_c}, \tag{S4}$$

where ξ is the Kondo parameter and ω_c is the cutoff frequency. The reorganization energy for the Ohmic spectral density is $E_r = 2\hbar\xi\omega_c$. The Ohmic spectral density can be discretized as follows

$$\omega_k = \omega_c \ln\left(\frac{N}{N-k+\frac{1}{2}}\right),\ k = 1,2,\cdots,N. \tag{S5}$$

For MSH S1–S9 (a-e) models, the number of physical modes $N = 60$, electronic energy matrix $\hat{H}_e = \sum_{i=1}^{3} \varepsilon_i |i\rangle\langle i| + \sum_{i>j}^{3} \Gamma_{ij}(|i\rangle\langle j| + |j\rangle\langle i|)$ is

$$\hat{H}_e = \begin{pmatrix} \varepsilon & \Gamma_a & \Gamma_b \\ \Gamma_a & -\varepsilon & \Gamma_a \\ \Gamma_b & \Gamma_a & 0 \end{pmatrix} \tag{S6}$$

and the PES shift matrix is defined as

$$\mathbf{A}_b = \begin{pmatrix} A_b^{(11)} & A_b^{(21)} \\ A_b^{(12)} & A_b^{(22)} \\ A_b^{(13)} & A_b^{(23)} \end{pmatrix} = \begin{pmatrix} 0 & \sqrt{2}s \\ s & \sqrt[4]{3}s \\ 0 & 0 \end{pmatrix}. \tag{S7}$$

The initial nuclear sampling is on $|3\rangle$ at 300 K. The detailed parameter values of MSH models S1–S9 are shown in Table S6.

TABLE S6. Model parameters of MSH models S1–S9(c), with energy unit in cm^{-1}. MSH models S1–S9(a, b, d, e) sets are tuned from (c) set: ω_c, Γ_a and Γ_b are half for (a) set; ω_c, Γ_a and Γ_b are three times for (e) set; s^2 and ε are three times for (b) set; s^2 and ε are half for (d) set.

Model	ω_c	s^2	ε	Γ_a	Γ_b
S1(c)	10	50	20	8	3
S2(c)	10	10	20	8	3
S3(c)	10	5	20	8	3
S4(c)	10	50	20	20	6
S5(c)	10	10	20	20	6
S6(c)	10	5	20	20	6
S7(c)	10	50	20	40	15
S8(c)	10	10	20	40	15
S9(c)	10	5	20	40	15

For RSV models, $N = 60$, their electronic energy matrix is

$$\hat{H}_e = \begin{pmatrix} 0 & \Gamma_a & \Gamma_a \\ \Gamma_a & \varepsilon & \Gamma_b \\ \Gamma_a & \Gamma_b & \varepsilon \end{pmatrix}, \tag{S8}$$

and the PES shift matrix is defined as

$$\mathbf{A}_b = \begin{pmatrix} 0 & 0 \\ s & 0 \\ s\cdot\cos\theta_{23} & s\cdot\sin\theta_{23} \end{pmatrix}, \tag{S9}$$

where $\theta_{23} = 45^\circ, 65^\circ, 80^\circ, 90^\circ, 100^\circ, 115^\circ$ and 135°. The initial nuclear sampling is performed on $|1\rangle$ at 300 K. The detailed parameter values of the RSV cases are shown in Table S7.

TABLE S7. Model parameters of MSH rotating shift vector (RSV) models, with energy unit in cm^{-1}.

Model Set	ω_c	ε	Γ_a	Γ_b	s^2
Strong coupling	10	100	10	100	100
Weak coupling	10	100	100	10	100

For Y6 dimer at a distance of 10 Å, the electronic energy matrix is

$$\hat{H}_e = \begin{pmatrix} 0 & 0 & 0 \\ 0 & 1.64 & 0.0505 \\ 0 & 0.0505 & 1.64 \end{pmatrix} \text{eV}, \tag{S10}$$

and the PES shift matrix is defined as

$$\mathbf{A}_b = \begin{pmatrix} 0 & 0 \\ 0.2701 & 0 \\ 0.0460 & 0.2638 \end{pmatrix} \mathrm{eV}^{-\frac{1}{2}}. \tag{S11}$$

The initial nuclear sampling is performed on $|1\rangle$ at 300 K. The discretized nuclear mode frequencies for $N = 20, 40$ and 60 are listed below in atomic unit:

1. $N = 20$:
4.2699896031976847 4908e-07, 1.2870687157332629 1699e-06, 2.1669749086621639 8037e-06, 3.0806024407748311 7120e-06, 4.0500704793070703 4705e-06, 5.0992107950686889 8246e-06, 6.2707611720336919 3330e-06, 7.6299765817450326 7726e-06, 9.2967596051730178 9253e-06, 1.1520909545202979 8979e-05, 1.4680096890153650 7227e-05, 1.8823784218008598 4591e-05, 2.3899772153715339 1853e-05, 3.1604144124536236 9843e-05, 4.2307965470790988 1203e-05, 5.9030185421669293 0069e-05, 8.4842534564686487 1802e-05, 1.3170123575250000 5714e-04, 3.1927127516340002 6793e-04, 5.6492104789470998 5743e-03

2. $N = 40$:
2.1348836360000000 2375e-07, 6.4090244589999998 5532e-07, 1.0707260549000000 7398e-06, 1.5046493611000000 4968e-06, 1.9443854640000001 7780e-06, 2.3916702662999998 3982e-06, 2.8482617134000001 2172e-06, 3.3159381937000001 3924e-06, 3.8015624568999999 5320e-06, 4.3035563270000002 5927e-06, 4.8275401033999997 0700e-06, 5.3782187333999999 8337e-06, 5.9613372113999998 5856e-06, 6.5894480910999998 9337e-06, 7.2672750609000000 5778e-06, 8.0114177096000007 0023e-06, 8.8414751319999999 6727e-06, 9.7909535264999994 3993e-06, 1.0894426537900000 7279e-05, 1.2206870808600000 8220e-05, 1.3785327779100000 7521e-05, 1.5640206271199999 9061e-05, 1.7718860244599999 2586e-05, 1.9974920215399999 5803e-05, 2.2481974287599999 6703e-05, 2.5488776901699999 6496e-05, 2.9366144097000000 9714e-05, 3.3940753509499997 7940e-05, 3.9106026045500000 1465e-05, 4.6005283101199998 2568e-05, 5.3971627380999997 6560e-05, 6.4612735694300006 1848e-05, 7.7678391300099999 0577e-05, 9.3875559347200005 2051e-05, 1.1638472752660000 1554e-04, 1.5147173345110001 2274e-04, 2.1952399775920000 0619e-04, 2.3904553980091001 6720e-03, 5.5078223428831002 7642e-03, 6.6275313895577002 0740e-03

3. $N = 60$:
1.4236952769999999 3702e-07, 4.2699896029999999 6468e-07, 7.1232702319999996 4274e-07, 9.9885001359999993 8860e-07, 1.2870687156999999 3315e-06, 1.5774884450999999 1230e-06, 1.8706190572000000 6773e-06, 2.1669749085999998 0885e-06, 2.4670747603999998 5812e-06, 2.7714416098000000 7314e-06, 3.0806024407000000 4547e-06, 3.3950878784999999 2364e-06, 3.7154317387000000 6739e-06, 4.0500704793000001 6304e-06, 4.3892440151000002 2707e-06, 4.7384942923999999 5377e-06, 5.0992107949999996 9263e-06, 5.4729796588000002 3155e-06, 5.8616169799000002 4079e-06, 6.2707611720000003 5079e-06, 6.6984756547000001 9957e-06, 7.1501415222999995 9751e-06, 7.6299765816999993 2358e-06, 8.1431707777999993 2432e-06, 8.6961321640999997 0134e-06, 9.2967596051000002 6435e-06, 9.9627337848999996 8805e-06, 1.0697552676600000 5919e-05, 1.1520909545200000 0360e-05, 1.2449930974999999 4000e-05, 1.3499298602299999 8885e-05, 1.4680096890099999 6558e-05, 1.5973176917099999 6833e-05, 1.7359882218099999 9565e-05, 1.8823784217999999 3806e-05, 2.0370981630200000 5020e-05, 2.2038562076899999 0486e-05, 2.3899772153700001 1127e-05, 2.6070372870300000 7897e-05, 2.8655526712899999 4347e-05, 3.1604144124499997 5267e-05, 3.4740533424700003 0664e-05, 3.8158890707200002 3857e-05, 4.2307965470700003 2293e-05, 4.7279965827500000 4573e-05, 5.2525709211599999 7452e-05, 5.9030185421599998 9356e-05, 6.6480589144200005 3242e-05, 7.5272941326500004 3371e-05, 8.4842534564599994 9519e-05, 9.7091805833499994 7104e-05, 1.1219400105300000 3056e-04, 1.3170123575250000 5714e-04, 1.5902429497509999 7123e-04, 2.0342951233440000 9041e-04, 3.1927127516340002 6793e-04, 2.9802066169466001 4482e-03, 5.1516887658056001 1064e-03, 5.6492104789470001 1083e-03, 7.9083177021425001 6904e-03

B. Numerical Simulation Parameters

For numerically exact calculations of the MSH models using tensor-train-based methods, the numerical parameters are included in Table S8. For the approximate calculations, the number of trajectories averaged for nonadiabatic semiclassical

mapping and mixed quantum-classical dynamics of RSV and Y6 dimer MSH models are 10^5, time step $\Delta t = 0.001$ ps. The numerical parameters for MSH models S1–S9 are listed as in Table S9, which was reported in Ref. 15.

TABLE S8. Numerical simulation parameters for numerically exact tensor-train-based dynamics of the MSH models S5(d) and Y6 dimer of different N, including temperature T, occupation number N_{occ}, and the time step τ and maximum TT-rank r_{max} when propagated to time t_{max}.

Model	T / K	N_{occ}	τ / a.u.	r_{max}	t_{max} / ps
S5(d)	0	20	25	25	5
S5(d)	77	20	12.5	50	2
S5(d)	300	20	10	30	1.1
Y6 dimer (N=20)	300	40	0.625	155	0.5
Y6 dimer (N=40)	300	20	0.3125	145	0.5
Y6 dimer (N=60)	300	25	10	70	0.23

TABLE S9. Numerical parameters for nonadiabatic semiclassical mapping and mixed quantum-classical dynamics of MSH models S1–S9.

Method	Δt / ps	N_{traj}	t_{max} / ps
FSSH	0.001	1×10^5	10
MF	0.001	2×10^5	10
LSC	0.001	6×10^5	10
SQC	0.001	3×10^5	10
CMM	0.001	4×10^5	10

REFERENCES

[1] Z. Hu and X. Sun, “All-Atom Nonadiabatic Semiclassical Mapping Dynamics for Photoinduced Charge Transfer of Organic Photovoltaic Molecules in Explicit Solvents,” J. Chem. Theory Comput. **18**, 5819–5836 (2022).

[2] M. J. Frisch, G. W. Trucks, H. B. Schlegel, G. E. Scuseria, M. A. Robb, J. R. Cheeseman, G. Scalmani, V. Barone, G. A. Petersson, H. Nakatsuji, *et al.*, “Gaussian16 Revision C.01,” Gaussian Inc. Wallingford CT (2016).

[3] J. J. P. Stewart, “Optimization of Parameters for Semiempirical Methods V: Modification of Nddo Approximations and Application to 70 Elements,” J. Mol. Model. **13**, 1173–1213 (2007).

[4] E. Epifanovsky, A. T. B. Gilbert, X. Feng, J. Lee, Y. Mao, N. Mardirossian, P. Pokhilko, A. F. White, M. P. Coons, *et al.*, “Software for the Frontiers of Quantum Chemistry: An Overview of Developments in the Q-Chem 5 Package,” J. Chem. Phys. **155**, 084801 (2021).

[5] C. Adamo and V. Barone, “Toward Reliable Density Functional Methods Without Adjustable Parameters: The PBE0 Model,” J. Chem. Phys. **110**, 6158–6170 (1999).

[6] S. Hirata and M. Head-Gordon, “Time-Dependent Density Functional Theory within the Tamm–Dancoff Approximation,” Chem. Phys. Lett. **314**, 291–299 (1999).

[7] A. A. Voityuk and N. Rösch, “Fragment Charge Difference Method for Estimating Donor–Acceptor Electronic Coupling: Application to DNA π-Stacks,” J. Chem. Phys. **117**, 5607–5616 (2002).

[8] W. D. Cornell, P. Cieplak, C. I. Bayly, I. R. Gould, K. M. Merz, D. M. Ferguson, D. C. Spellmeyer, T. Fox, J. W. Caldwell, and P. A. Kollman, “A second generation force field for the simulation of proteins, nucleic acids, and organic molecules,” J. Am. Chem. Soc. **117**, 5179–5197 (1995).

[9] J. Wang, R. M. Wolf, J. W. Caldwell, P. A. Kollman, and D. A. Case, “Development and Testing of a General Amber Force Field,” J. Comput. Chem. **25**, 1157–1174 (2004).

[10] P. Eastman, J. Swails, J. D. Chodera, R. T. McGibbon, Y. Zhao, K. A. Beauchamp, L.-P. Wang, A. C. Simmonett, M. P. Harrigan, C. D. Stern, R. P. Wiewiora, B. R. Brooks, and V. S. Pande, “OpenMM 7: Rapid Development of High Performance Algorithms for Molecular Dynamics,” PLOS Comput. Biol. **13**, 1–17 (2017).

[11] T. Darden, D. York, and L. Pedersen, “Particle Mesh Ewald: An Nlog(N) Method for Ewald Sums in Large Systems,” J. Chem. Phys. **98**, 10089–10092 (1993).

[12] G. Ciccotti and J. P. Ryckaert, “Molecular Dynamics Simulation of Rigid Molecules,” Comput. Phys. Rep. **4**, 346–392 (1986).

[13] Z. Liu and X. Sun, “Direct All-Atom Nonadiabatic Semiclassical Simulations for Electronic Absorption Spectroscopy of Organic Photovoltaic Non-Fullerene Acceptor in Solution,” J. Phys. Chem. Lett. **16**, 4463–4473 (2025).

[14] Y. Guo, G. Han, and Y. Yi, “The Intrinsic Role of the Fusion Mode and Electron-Deficient Core in Fused-Ring Electron Acceptors for Organic Photovoltaics,” Angew. Chem. Int. Ed. **61**, e202205975 (2022).

[15] H. Zeng and X. Sun, “Quantum Dynamics in Multistate Harmonic Models using Tensor-Train Thermofield Dynamics and Semiclassical Mapping Dynamics,” J. Chem. Phys. **163**, 024131 (2025).
